# Computing Multivariate Effect Sizes and Their Sampling Covariance Matrices With Structural Equation Modeling: Theory, Examples, and Computer Simulations

**DOI:** 10.3389/fpsyg.2018.01387

**Published:** 2018-08-17

**Authors:** Mike W.-L. Cheung

**Affiliations:** Department of Psychology, National University of Singapore, Singapore, Singapore

**Keywords:** effect size, multivariate effect size, sampling covariance matrix, meta-analysis, structural equation model

## Abstract

In the social and behavioral sciences, it is recommended that effect sizes and their sampling variances be reported. Formulas for common effect sizes such as standardized and raw mean differences, correlation coefficients, and odds ratios are well known and have been well studied. However, the statistical properties of multivariate effect sizes have received less attention in the literature. This study shows how structural equation modeling (SEM) can be used to compute multivariate effect sizes and their sampling covariance matrices. We focus on the standardized mean difference (multiple-treatment and multiple-endpoint studies) with or without the assumption of the homogeneity of variances (or covariance matrices) in this study. Empirical examples were used to illustrate the procedures in R. Two computer simulation studies were used to evaluate the empirical performance of the SEM approach. The findings suggest that in multiple-treatment and multiple-endpoint studies, when the assumption of the homogeneity of variances (or covariance matrices) is questionable, it is preferable not to impose this assumption when estimating the effect sizes. Implications and further directions are discussed.

In the social and behavioral sciences, it is recommended that effect sizes and their sampling variances be reported (e.g., Cohen, 1994; Wilkinson and Task Force on Statistical Inference, 1999; Cumming, 2014). When there are a sufficient number of studies, the meta-analysis is the standard method used to synthesize the research findings. The results of the meta-analysis may inform us what the average effect is and how the effect sizes vary across the studies.

There are two key ingredients for a meta-analysis. The first one is the effect size that quantifies the strength of the effect in the studies. Effect sizes can be either unstandardized or standardized (e.g., Kelley and Preacher, 2012). Unstandardized effect sizes are used when the effect sizes are comparable across studies, e.g., blood pressure or physical measures (Bond et al., 2003). When the scales of the measures are unclear or non-comparable across studies, standardized effect sizes are preferred (e.g., Hunter and Hamilton, 2002).

Besides the effect sizes, we also need the standard error (*SE*) of the effect sizes to quantify the precision of the estimated effect sizes. Formulas for common effect sizes such as the standardized and raw mean differences, correlation coefficients, and odds ratios are well known and have been well studied (Borenstein et al., 2009; Card, 2012; Cheung, 2015a; Schmidt and Hunter, 2015).

In applied research, however, more than one effect size may be involved. For example, there may be more than one treatment group compared to a control group. The use of multiple treatment groups allows researchers to address the phenomenon under different levels of manipulation. By using the same control group in the comparisons, researchers minimize the cost of collecting multiple control groups (Kim and Becker, 2010). Another example is when there is more than one outcome variable in the control and treatment groups. The use of multiple outcomes permits researchers to study different related outcomes under the same manipulations (Thompson and Becker, 2014). Studies that measure these two types of effect sizes are known as multiple-treatment and multiple-endpoint studies.

Since the effect sizes are not independent, researchers have to calculate the sampling covariances among the effect sizes. Gleser and Olkin (1994, 2009) have provided the most comprehensive treatment of this subject to date. They derived formulas to compute the effect sizes and their sampling variances and covariances. Once the effect sizes and their sampling covariance matrices are available, a multivariate meta-analysis (Nam et al., 2003; Jackson et al., 2011; Cheung, 2013) can be performed on all effect sizes.

Although Gleser and Olkin (1994, 2009) have provided standard formulas to compute the effect sizes and their sampling covariance matrices for multiple-treatment and multiple-endpoint studies, there are a few limitations in their approach. First, it is not easy for users, especially those without a strong statistical background, to comprehend the logic in calculating the variances and covariances. Second, these formulas rely on the assumption of the homogeneity of variances or covariance matrices. Although it is possible to drop these assumptions, the derivations are not apparent for most users. Most users would just adopt these assumptions without considering the alternatives. Third, it is difficult to extend their formulas to more complicated cases. One such example is the combination of multiple-treatment with multiple-endpoint studies in the same publication. Many researchers simplify the effect sizes to either the multiple-treatment study or the multiple-endpoint study, which is not ideal because of the loss of information.

Structural equation modeling (SEM) is a favorite tool to use in analyzing multivariate data. It has been used to calculate *SE*s and confidence intervals for various effect sizes and indices (Raykov, 2001; Cheung and Chan, 2004; Preacher, 2006). Recently, Cheung (2015a, Chapter 3) showed how common effect sizes, including those in multiple-treatment and multiple-endpoint studies, and their sampling variances and covariances, can be computed using the SEM framework.

The SEM approach provides a graphical model of means, standard deviations, and correlations. The effect sizes are defined as functions of these parameters. Readers can get a better understanding of what these effect sizes mean. Second, assumptions of the homogeneity of variances, covariances, or correlations can be imposed or relaxed by the use of equality constraints on the parameters. By using the delta method built into the SEM packages, appropriate sampling covariance matrices can be automatically derived. Third, it is feasible to extend the SEM approach to more complicated situations. For example, the SEM approach can be used to calculate the effect sizes and their sampling covariance matrix for a combination of multiple-treatment and multiple-endpoint studies^1^ The key advantage of this is that researchers only need to focus on the conceptual “definition” of the effect sizes; the sampling covariance matrix of the effect sizes is numerically calculated by the SEM packages.

The rest of this article is structured as follows. The next section contains a brief introduction on how to compute the effect sizes and their sampling covariance matrices for the multiple-treatment and multiple-endpoint designs in SEM. Two empirical examples are used to illustrate how to conduct the analyses using the metaSEM package (Cheung, 2015b) implemented in the R statistical platform (R Development Core Team, 2018). Two computer simulations are then presented to evaluate the empirical performance of the SEM approach under several conditions. Based on the findings of the simulation, this paper concludes that it is preferable not to impose the assumption of the homogeneity of variances (or covariances) when calculating the effect sizes for multiple treatment and multiple-endpoint studies when this assumption is questionable. Finally, further directions for further research are discussed.

## A SEM approach to estimating effect size

Cheung (2015a, Chapter 3) presents a SEM approach to estimating various effect sizes, including those in multiple-treatment and multiple-endpoint studies. There are three steps in the analysis. In the first step, a structural equation model with means, standard deviations, and correlations is proposed to fit the data. When the data are from independent groups (e.g., control vs. intervention groups in calculating the standardized or raw mean differences) a multiple-group structural equation model is used. Second, appropriate equality constraints on the homogeneity of covariance (or correlation) matrices are imposed. If there are reasons to believe that the assumption of the homogeneity of covariance (or correlation) matrices is not appropriate, researchers may test the hypothesis statistically. They may then choose to drop these assumptions when calculating the effect sizes.

Finally, the effect sizes are defined as functions of the means and standard deviations (*SD*s). The effect sizes with their sampling covariance matrices are estimated by the SEM packages using maximum likelihood (ML) estimation. This approach releases users from the need to manually derive the sampling covariance matrix, a process that is prone to human error. Let us consider examples of multiple-treatment and multiple-endpoint studies.

### Multiple-treatment studies

**Figure 1:**
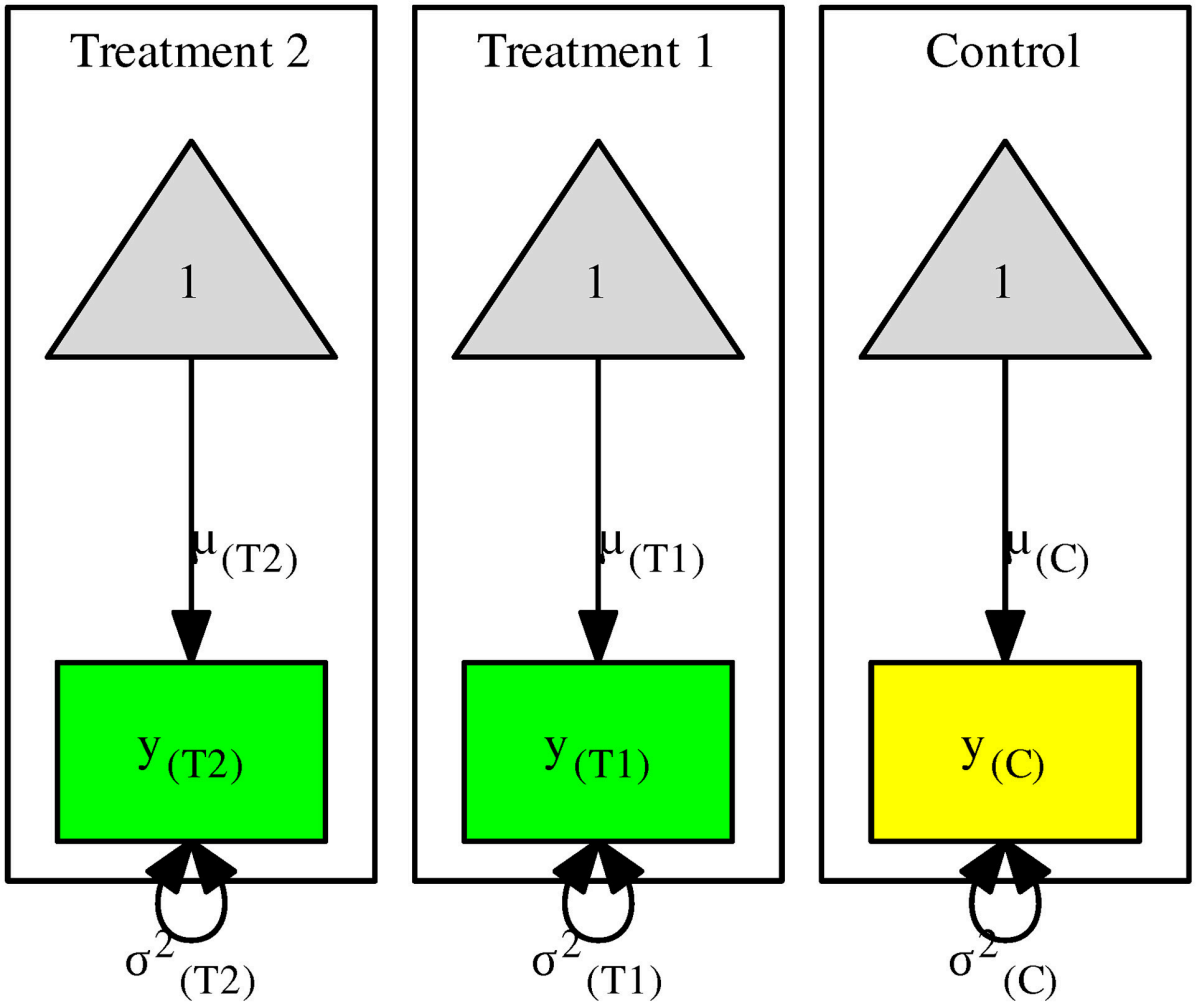
The structural equation model for the multiple-treatment studies.

Suppose that we measure the mathematics score in a control group and two treatment groups (*y*_(*C*)_,*y*_(*T*1)_, and *y*_(*T*2)_). Figure 1 shows a structural equation model with one control and two treatment groups. For ease of discussion, we use the population parameters in the figures. It is understood that sample estimates are employed in the analyses. The rectangles and the triangles represent the observed variables and columns of ones, respectively. The arrows from the triangles to the observed variables represent the means of the variables in the control μ_(*C*)_, treatment 1 μ_(*T*1)_, and treatment 2 μ_(*T*2)_, respectively. The variances of the variables in the control and treatments 1 and 2 are represented by $\sigma_{\left(C\right)}^{2}$, $\sigma_{\left(T1\right)}^{2}$, and $\sigma_{\left(T2\right)}^{2}$, respectively.

When no constraint is imposed, the above means and variances are the same as those of the sample statistics. Under the assumption of the homogeneity of variances, we may impose the constraint $H_{0}:\sigma_{Common}^{2}=\sigma_{\left(C\right)}^{2}=\sigma_{\left(T1\right)}^{2}=\sigma_{\left(T2\right)}^{2}$. This null hypothesis is tested by comparing the likelihood ratio (*LR*) statistics of the models with and without the constraint. If the null hypothesis is correct, the difference between the *LR* statistics follows a chi-square distribution with 2 degrees of freedom (*df* s). We may now define the standardized mean differences (SMDs) between the treatment groups and the control by using the common *SD* σ_*Common*_ as the denominator:

(1) $$\begin{array}{l} SMD_{\text{MTS}1}=\frac{\mu_{\left(T1\right)}-\mu_{\left(C\right)}}{\sigma_{\text{Common}}}\;and\;SMD_{MTS2}=\frac{\mu_{\left(T2\right)}-\mu_{\left(C\right)}}{\sigma_{\text{Common}}}. \end{array}$$

One unit of SMD indicates that the mean of the treatment group is one common *SD* above that of the control group. Since *SMD*_MTS1_ and *SMD*_MTS2_ share the same parameters μ_(*C*)_ and σ_Common_, they are correlated. Instead of using the analytic solutions provided by Gleser and Olkin (1994, 2009), we may estimate the sampling variances and the covariance by the numerical approach in SEM.

When the assumption of the homogeneity of variances is questionable, it may not be appropriate to use σ_Common_ in the denominator. This is because σ_Common_ is not estimating any of the population *SD*s. A better alternative is to use the control group σ_(*C*)_ as the standardizer in calculating the effect sizes (Glass et al., 1981). The standardized mean differences of the treatment groups against the control group are now described as:

(2) $$\begin{array}{l} SMD_{\text{MTS}1}=\frac{\mu_{\left(T1\right)}-\mu_{\left(C\right)}}{\sigma_{\left(C\right)}}\;and\;SMD_{\text{MTS}2}=\frac{\mu_{\left(T2\right)}-\mu_{\left(C\right)}}{\sigma_{\left(C\right)}}, \end{array}$$

which does not rely on the assumption of the homogeneity of variances. Now, one unit of SMD indicates that the mean of the treatment group is one *SD* of the control group above that of the control group.

### Multiple-endpoint studies

**Figure 2:**
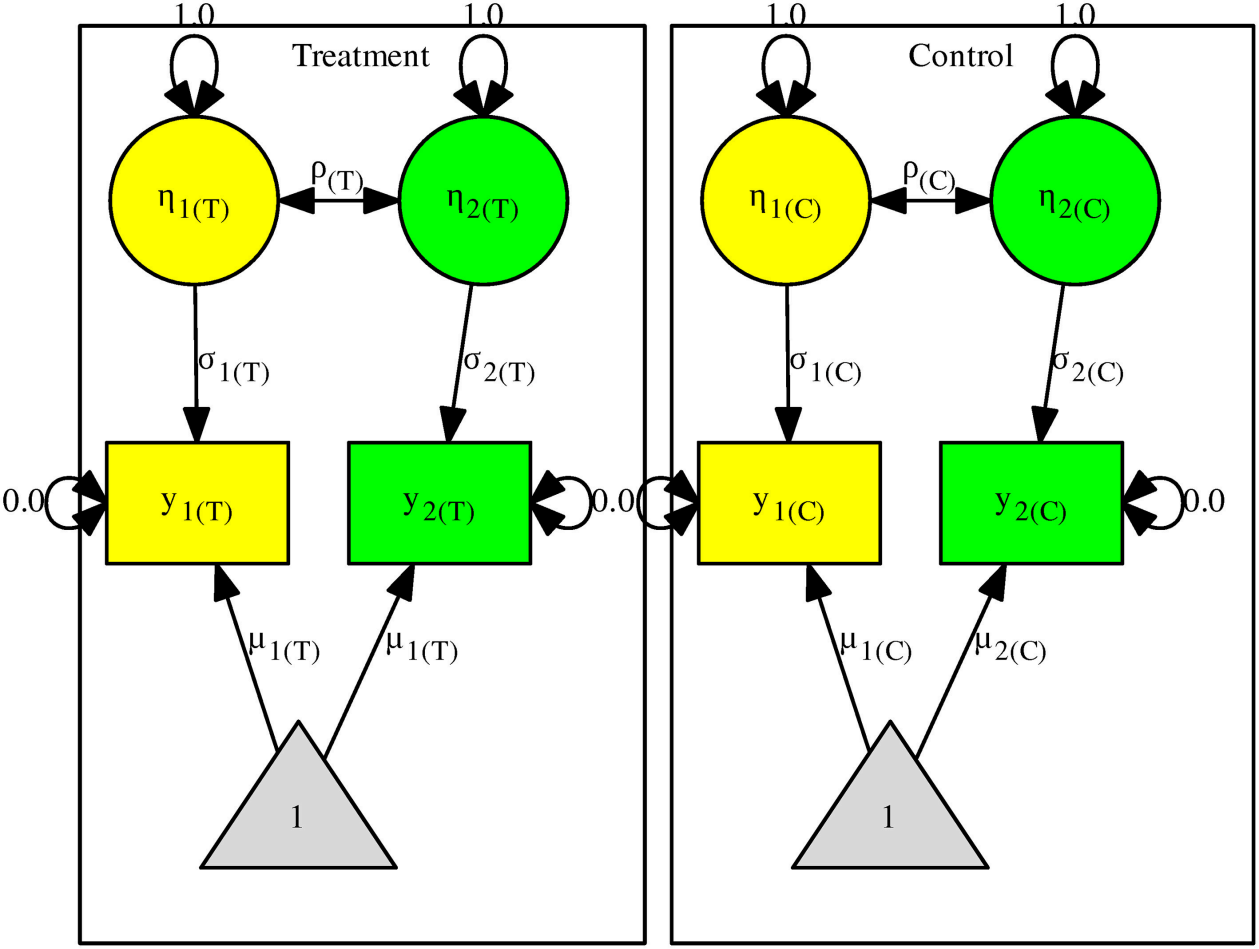
The structural equation model for the multiple-endpoint studies.

Now suppose that there are two effect sizes on the mathematics and language scores *y*_1_ and *y*_2_. Figure 2 shows the model with two independent groups (the control and treatment groups). We use η_1_ and η_2_, with their variances fixed at one, to represent the standardized scores of *y*_1_ and *y*_2_. σ_1_ and σ_2_ now represent the *SD*s of *y*_1_ and *y*_2_. The same model representation is often used to standardize the variables in SEM (e.g., Cheung and Chan, 2004, 2005; Cheung, 2015a).

We may assume that the correlations are homogeneous by imposing the constraint *H*_0_:ρ_Common_ = ρ_(*C*)_ = ρ_(*T*)_. An *LR* test can be used to test this null hypothesis by comparing the models with and without this constraint. Under the null hypothesis, the test statistic has a chi-square distribution with 1 *df*. If we further assume that the covariance matrices are homogeneous, we may impose the constraints of *H*_0_:ρ_Common_ = ρ_(*C*)_ = ρ_(*T*)_, *H*_0_:σ_1Common_ = σ_1(*C*)_ = σ_1(*T*)_, and *H*_0_:σ_2Common_ = σ_2(*C*)_ = σ_2(*T*)_. Under the null hypothesis, the test statistic on comparing the models with and without the constraints follows a chi-square distribution with 3 *df* s. We may drop all of the constraints if these assumptions are questionable.

Regardless of whether we have imposed the above constraints, the effect sizes for the multiple-endpoint study are defined as:

(3) $$\begin{array}{l} SMD_{\text{MES}1}=\frac{\mu_{1\left(T\right)}-\mu_{1\left(C\right)}}{\sigma_{1}}\;and\;SMD_{\text{MES}2}=\frac{\mu_{2\left(T\right)}-\mu_{2\left(C\right)}}{\sigma_{2}}, \end{array}$$

where σ_1_ and σ_2_ are the standard deviations for *y*_1_ and *y*_2_. We do not put the subscript in the formulas because what σ_1_ and σ_2_ actually are depends on whether constraints have been imposed on them. If we impose the equality constraints on the *SD*s, σ_1Common_ and σ_2Common_ are used as the standardizers in Equation (3). If we do not assume that the covariance matrices are homogeneous, the *SD*s in the control groups (σ_1(*C*)_ and σ_2(*C*)_) are used as the standardizers. Once we have defined the appropriate effect sizes, the sampling covariance matrix between *SMD*_MES1_ and *SMD*_MES2_ can be obtained from the SEM packages with numerical methods.

### Illustrations with R

Gleser and Olkin (1994) presented some sample data on the multiple-treatment and multiple-endpoint studies. These datasets are stored in the metaSEM package (Cheung, 2015b). The metaSEM package also provides smdMTS() and smdMES() to calculate the effect sizes for a multiple-treatment study and a multiple-endpoint study with or without the assumptions of homogeneity. Supplementary Materials 5 shows the sample R code. Readers may refer to the package manual for details.

Table 22.2 in Gleser and Olkin (1994) displays simulated data from six studies on five modes of exercise with a control group of no regular exercise. The dependent variable is systolic blood pressure. Therefore, a negative effect size between the treatment and control groups suggests that those in the treatment groups are in better health than those in the control group. As an illustration, we show the calculations from the first study, which includes three treatment groups and one control group. When we assume that the variances are homogeneous, the *SMD*_MTS_ of the three treatment groups compared to the control group are −1.17, −1.90, and −2.00, respectively. The sampling covariance matrix is $\left(\begin{array}{lll} 0.09 & \; & \;\\ 0.05 & 0.10 & \;\\ 0.05 & 0.06 & 0.10 \end{array}\right)$. If we do not assume that the variances are homogeneous and use the *SD* of the control group as the standardizer, the *SMD*_MTS_ are −0.79, −1.29, and −1.36, respectively. The sampling covariance matrix is $\left(\begin{array}{lll} 0.06 & \; & \;\\ 0.06 & 0.09 & \;\\ 0.06 & 0.07 & 0.08 \end{array}\right)$. In this example, the effect sizes that were calculated with the assumption that the variances are homogeneous and are about 50% larger than those that were calculated without this assumption. When testing the assumption that the variances are homogeneous, the statistic is $\chi_{\left(3\right)}^{2}$ = 21.30, *p* < 0.001, which suggests that this assumption is not tenable. It is questionable whether the use of effect sizes with the assumption of the homogeneity of variances is appropriate in this example.

Table 22.4 in Gleser and Olkin (1994) shows seven published studies on the SAT-Math and SAT-Verbal scores of groups that had been coached on the tests compared to the scores of uncoached control groups. A positive effect size means that the coached groups performed better than the uncoached groups. As an illustration, we select the first study for demonstration. The *SMD*_MES_ on Math and Verbal are 1.19 and 0.61 with $V_{\text{MES}}=\left(\begin{array}{ll} 0.09 & \;\\ 0.05 & 0.08 \end{array}\right)$. If we do not assume that the covariance matrices are homogeneous, the *SMD*_MES_ on Math and Verbal are 1.30 and 0.56 with $V_{\text{MES}}=\left(\begin{array}{ll} 0.12 & \;\\ 0.05 & 0.06 \end{array}\right)$. The test statistic on the homogeneity of covariance matrices is $\chi_{\left(3\right)}^{2}$ = 4.92, *p* = 0.18, which is not statistically significant. It should be noted that the sample sizes in these studies are quite small (at 34 and 21).

The above illustrations show that the effect sizes with and without the assumption of homogeneity may be very different depending on whether the homogeneity assumption holds. It remains unclear how these effect size estimates would work empirically in simulated data. The following computer simulation clarifies the empirical performance of these estimators.

## Two simulation studies

Two computer simulation studies were conducted to evaluate the empirical performance of the SEM approach. All of the simulations were performed with the metaSEM package (Cheung, 2015b) in the R statistical platform (R Development Core Team, 2018).

Before moving on to details of the simulation studies, it is essential to clarify the meanings of “with and without the homogeneity of variances (or covariance matrices)” in the simulation studies. The data are generated from either equal or unequal population variances (see the conditions of the Population Variances). Regardless of whether or not the population variances are equal, two sets of effect sizes are calculated from the same set of data—one assumes the homogeneity of variances, and the other does not.

When the data are generated from populations with equal variances, the effect sizes both with and without the homogeneity assumption should be correct. By assuming that the variances are homogeneous, which is correct in the generated data, the sampling variances of the effect sizes with the homogeneity assumption are usually smaller than those effect sizes without the homogeneity assumption. When the data are generated from unequal population variances, the effect sizes without the homogeneity assumption should still be correct. However, the effect sizes with the homogeneity assumption are likely to be biased because the model is misspecified. The present simulation studies evaluated the empirical performance of the computed effect sizes with and without the homogeneity assumption.

### Study 1: multiple-treatment studies

For the multiple-treatment studies, multivariate normal data were generated from the known data structures with or without the assumption of the homogeneity of variances.

#### Methods

In this simulation study, there was a control group with two treatment groups. Several factors were manipulated in the simulation study:

##### Population means

The population mean of the control group was fixed at 0 for reference. Six levels were used for the simulation study. The population means for the two treatment groups were (0.2, 0.2), (0.2, 0.5), (0.2, 0.8), (0.5, 0.5), (0.5, 0.8), and (0.8, 0.8).

##### Population variances

The population variance of the control group was fixed at 1 for reference. Three levels were selected for the simulation. The population variances for the two treatment groups were (1, 1), (0.75, 1.25), and (0.5, 1.5). When the population variance was (1, 1) in the two treatment groups, the homogeneity of variances was assumed. In the other levels, the population variances were heterogeneous. As the population variance of the control group was fixed at 1, the population effect size was calculated by the difference in means between the treatment groups and the control group divided by 1. Thus, the effect sizes were 0.2, 0.5, and 0.8, which represent the typical values observed in the social and behavioral sciences.

##### Sample sizes

The design was assumed to be balanced. Three levels of sample sizes were selected, namely, 30, 50, and 100. These levels should be representative of typical research settings.

Thus, there were a total of 6 × 3 × 3 = 54 conditions. One thousand replications were repeated for each condition.

##### Assessment of the empirical performance

Since the population mean and variance of the control were set at 0 and 1, respectively, the population effect sizes were defined as the mean differences between the treatment 1 (or 2) to the control group. The relative percentage bias of each effect size was computed as

(4) $$\begin{array}{l} B\left(\hat{\theta }\right)=\frac{\bar{\hat{\theta }}-\theta }{\theta }\times 100\%, \end{array}$$

where θ is the population effect size and $\bar{\hat{\theta }}$ is the mean of the estimates of the effect size $\hat{\theta }$ across the 1,000 replications. Proper estimation methods should have a relative bias of less than 5% (Hoogland and Boomsma, 1998). Since there were two effect sizes for two treatment groups, we reported the average of their absolute biases $B(\hat{\theta })=\left(\left|B{\left(\hat{\theta }\right)}_{\text{T1}}\right|+\left|B{\left(\hat{\theta }\right)}_{\text{T2}}\right|\right)/2,\text{ where }\left|B{\left(\hat{\theta }\right)}_{\text{T1}}\right|$ and $\left|B{\left(\hat{\theta }\right)}_{\text{T}2}\right|$ are the absolute biases for treatments 1 and 2, for ease of presentation.

When there is only one effect size, we may quantify the accuracy of its uncertainty by the use of the relative bias of the *SE*. Since there were two sampling variances and one sampling covariance, we used the sampling variances (*SE*^2^) and covariance as the measure of uncertainty,

(5) $$\begin{array}{l} B\left(\bar{Var}\left(\hat{\theta }\right)\right)=\frac{\bar{SE^{2}}\left(\hat{\theta }\right)-Var\left(\hat{\theta }\right)}{Var\left(\hat{\theta }\right)}, \end{array}$$

where $Var\left(\hat{\theta }\right)$ is the empirical variance (or covariance) of $\hat{\theta }$ and $\bar{SE^{2}}\left(\hat{\theta }\right)$ is the mean of the *SE*^2^ or sampling covariance across 1,000 replications. Since there were three biases for the two effect sizes and their covariance, we reported the average of their absolute biases.

(6) $$\begin{array}{l} B\left(\bar{Var}\left(\hat{\theta }\right)\right)=\left(\left|B\left(\bar{Var}\left({\hat{\theta }}_{1}\right)\right)|+|B\left(\bar{Var}\left({\hat{\theta }}_{2}\right)\right)\right|\right)\\ \;+\left(\left|B\left(\bar{Cov}\left({\hat{\theta }}_{1},{\hat{\theta }}_{2}\right)\right)\right|\right)/3, \end{array}$$

where $\left|B\left(\bar{Var}\left({\hat{\theta }}_{1}\right)\right)\right|$, $\left|B\left(\bar{Var}\left({\hat{\theta }}_{2}\right)\right)\right|$ and $\left|B\left(\bar{Cov}\left({\hat{\theta }}_{1},{\hat{\theta }}_{2}\right)\right)\right|$ are the absolute biases for the outcomes 1 and 2 and their covariance. Hoogland and Boomsma (1998) suggested that a proper estimation method should have a relative percentage bias of 10% on the *SE*. That is, the estimated *SE* should be within 0.90–1.1 of the empirical *SD* of $\hat{\theta }$. As we were using the sampling variance (*SE*^2^, not *SE*), we used (1.1^2^ – 1)≈20% as an indicator of good performance in estimating the sampling covariance matrix.

In the review process, one reviewer suggested displaying the individual parameter estimates ${\hat{\theta }}_{1}$ and ${\hat{\theta }}_{2}$. Due to space constraints, we put these results of the multiple-treatment studies in Supplementary Materials 1. Moreover, the same reviewer also suggested checking the performance under unbalanced sample sizes. We reran the simulation studies by introducing unbalanced sample sizes. The levels of sample sizes for the control, treatment 1, and treatment 2 groups were (100, 30, 50), (100, 50, 30), (30, 100, 50), (30, 50, 100), (50, 100, 30), and (50, 30, 100). The other factors were identical to the previous simulations. The results of the multiple-treatment and multiple-endpoint studies are shown in Supplementary Materials 2.

#### Results

The results were summarized in the heat maps, which provide an easy way to visualize the performance of the statistics. The x- and y-axes represent the population means and population variances separated by the sample sizes. A lighter color indicates a smaller bias than values with a darker color. When the bias is larger than the cut-off point (5% for the mean and 20% for the sampling variances or covariances), the color becomes gray.

**Figure 3:**
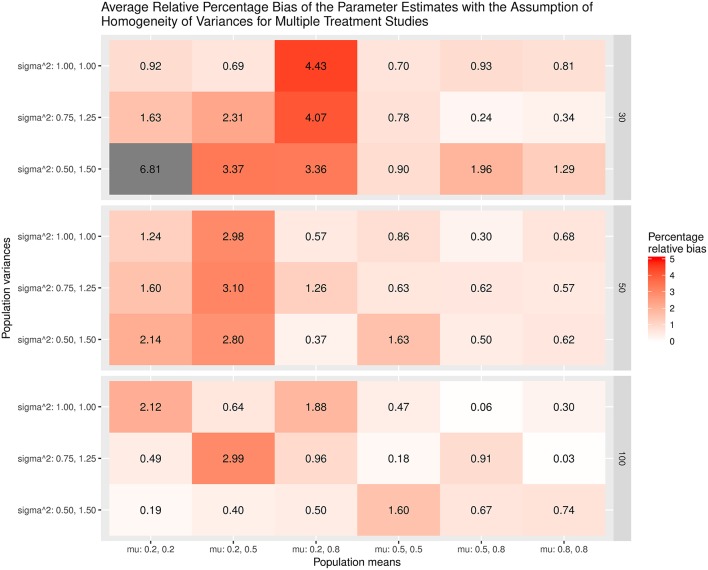
Relative bias of the average of the parameter estimates for the multiple treatment studies with the assumption of homogeneity of variances.

**Figure 4:**
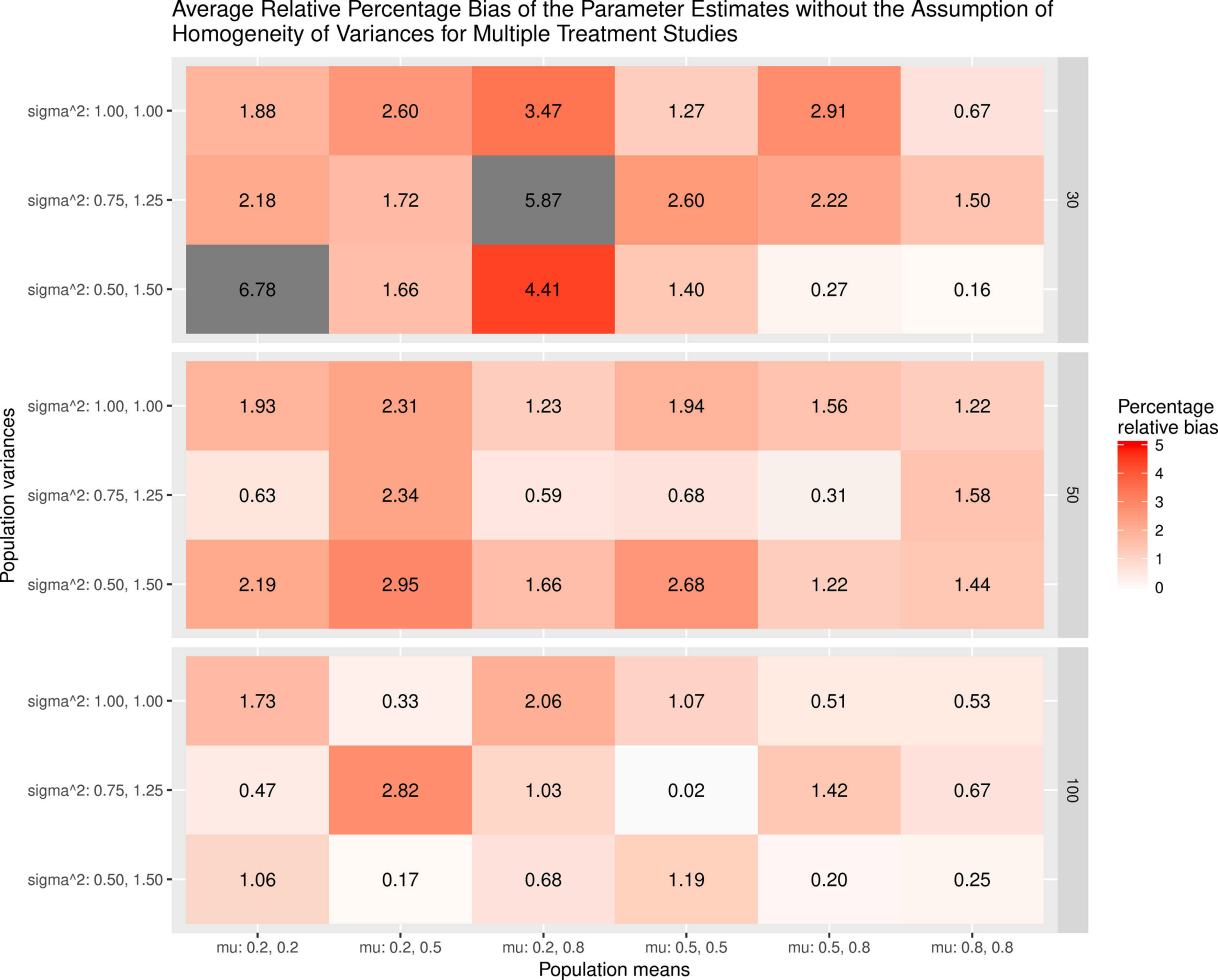
Relative bias of the average of parameter estimates for the multiple treatment studies without the assumption of homogeneity of variances.

Figures 3,4 show the relative bias of the effect sizes with and without the assumption of homogeneity of variances in calculating the effect sizes, respectively. One interesting finding was that the estimated effect size was generally unbiased regardless of whether or not the homogeneity of variances was assumed in the calculations. One speculation is that the average variances of the control group, which are always 1, and those of the treatment groups, at (0.75, 1.25) and (0.5, 1.5), are very close to 1. When these common *SD*s are used as the standardizers, the calculated effect sizes are still unbiased. The bias shrinks when the sample size gets bigger.

**Figure 5:**
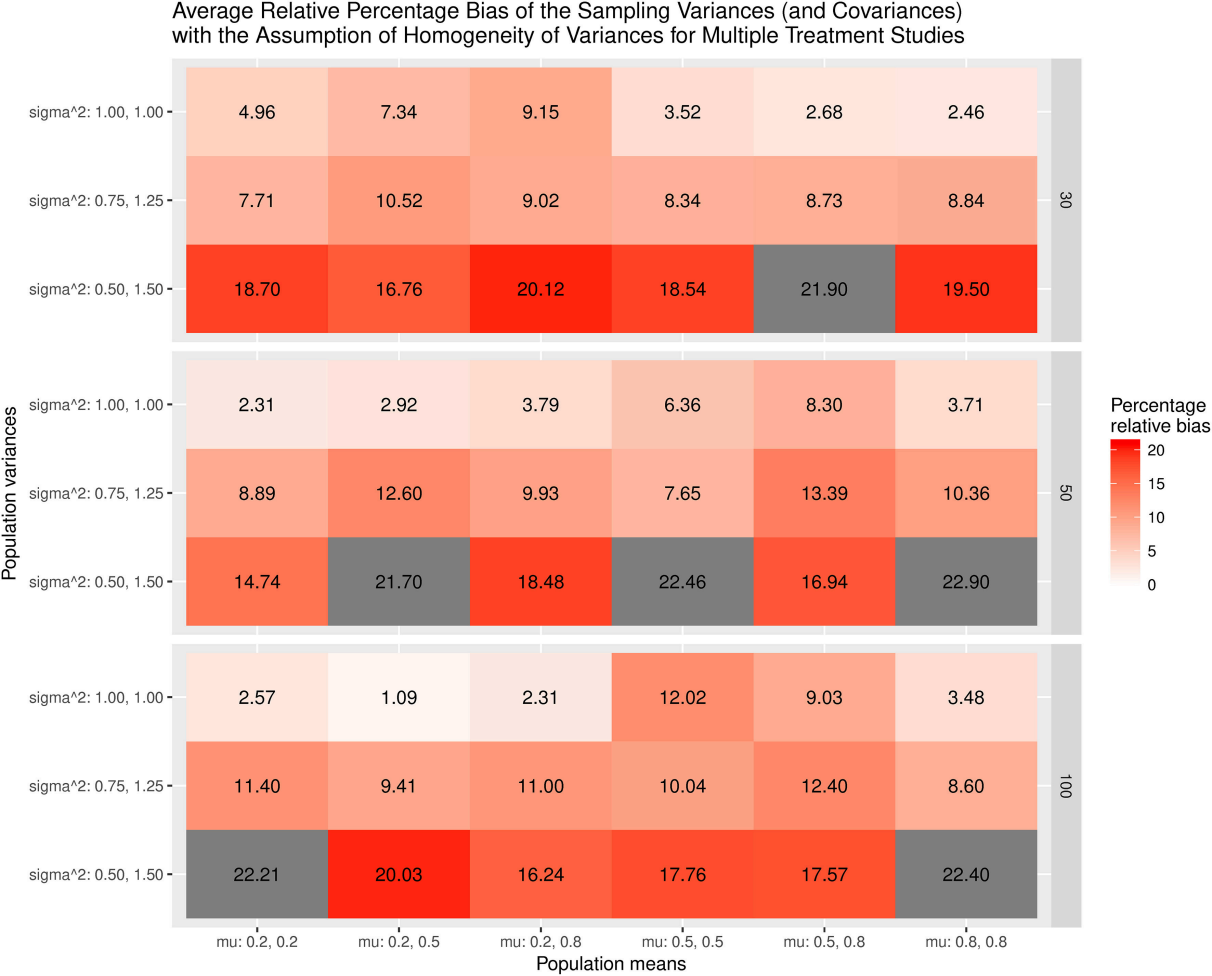
Relative bias of the average of the sampling variances and covariance for the multiple treatment studies with the assumption of homogeneity of variances.

**Figure 6:**
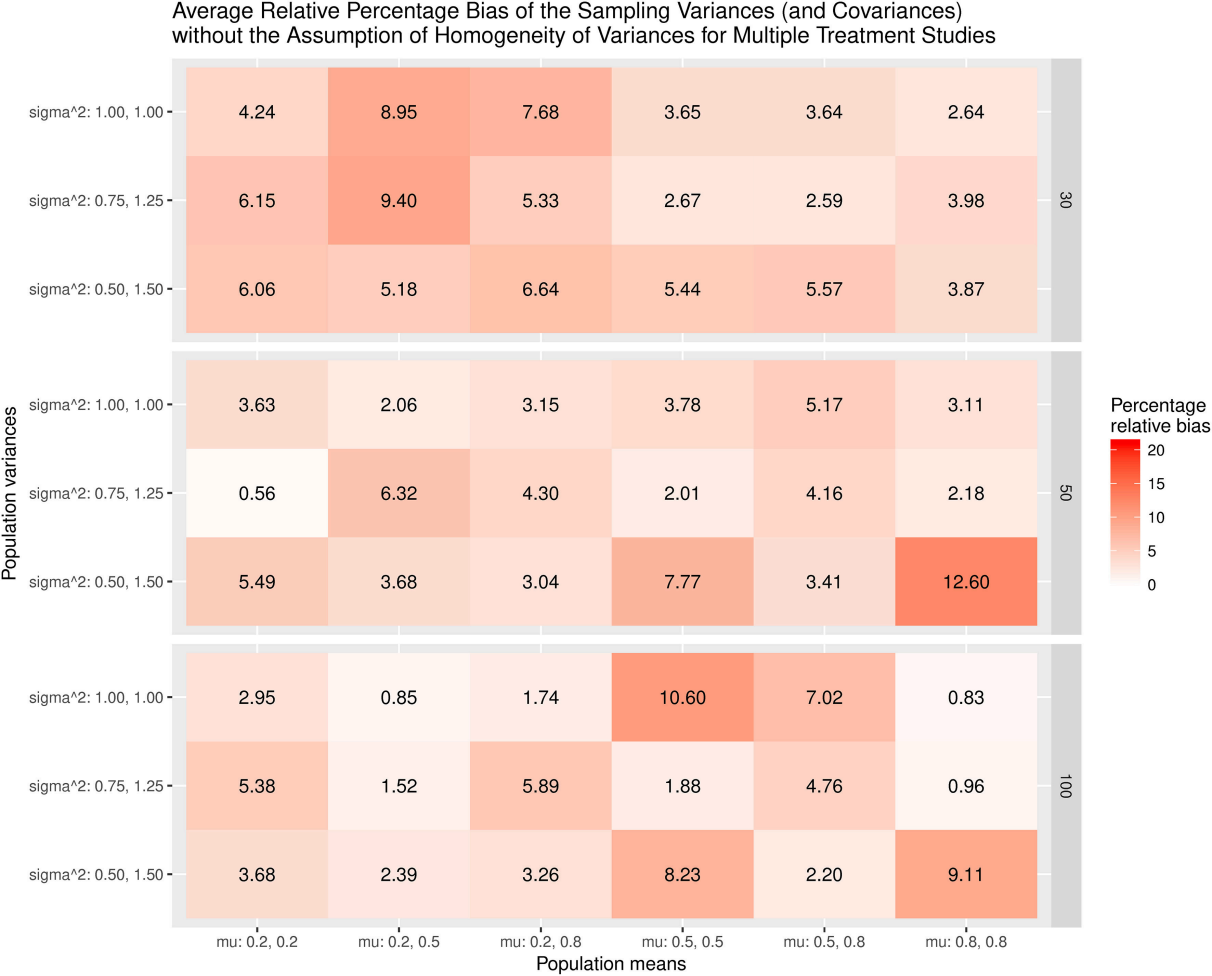
Relative bias of the average of the sampling variances and covariance for the multiple treatment studies without the assumption of homogeneity of variances.

Figure 5 displays the relative bias of the sampling variances and covariances when the variances are assumed to be homogeneous when the effect sizes are estimated. The findings show that the sampling variances and covariances are unbiased only when the variances are actually homogeneous. When the population variances are heterogeneous, the sampling variances and covariances are biased. The most substantial bias occurs when the population variances have the largest differences (sigma∧2: 0.5, 1.5). Figure 6 shows the relative bias of the sampling variances and covariances when the variances are not assumed to be homogeneous when estimating the effect sizes. In general, the bias is minimal, with the largest being only 12.6.

As a whole, the findings indicate that the effect sizes for the multiple treatment studies are estimated to be unbiased regardless of whether or not the homogeneity of variances is assumed in the calculations, given that the average of the treatment group variances are similar to that of the control group variance. However, the sampling variances and covariances are likely biased when the population variances are heterogeneous.

The patterns for the individual parameters in Supplementary Material 1 are similar to those of the average parameters. Therefore, we will not reproduce them here. Regarding the simulation results of the unbalanced sample sizes in Supplementary Material 2, the estimated effect sizes with the homogeneity assumption are unbiased when the sample sizes in the control group are large (100, 30, 50) and (100, 50, 30). However, the bias of the estimated effect sizes with the homogeneity assumption becomes larger when the sample size of the control group is small, and the sample sizes in the treatment groups are unbalanced. The bias of the estimated effect sizes without the homogeneity assumption is generally small. Regarding the sampling variances and covariance, they are generally biased with the assumption of homogeneity, whereas they are generally unbiased without the assumption of homogeneity.

### Study 2: multiple-endpoint studies

The design was similar to those in the multiple-treatment studies. Two effect sizes were used in the simulation study, with one control group and one intervention group.

#### Methods

The population means and variances of the control group were fixed at 0 and 1, respectively, for reference. The population correlation between these two outcomes was set at 0.3, which is considered moderate in psychological research.

##### Population means

Six levels were used in the simulation study. The means for the two outcome variables in the intervention group were (0.2, 0.2), (0.2, 0.5), (0.2, 0.8), (0.5, 0.5), (0.5, 0.8), and (0.8, 0.8).

##### Population variances

Five levels for the intervention group were selected for the simulation. They were (1, 1), (0.5, 0.5), (0.75, 0.75), (1.25, 1.25), and (1.5, 1.5). When the population variance of the intervention group is (1, 1), the homogeneity of covariance matrices between studies is assumed; the assumption of the homogeneity of variances does not hold in the population.

##### Sample sizes

The design was assumed to be balanced. Three levels of sample sizes were selected, namely, 30, 50, and 100.

Therefore, there were a total of 6 × 5 × 3 = 90 conditions. One thousand replications were repeated for each condition.

##### Assessment of the empirical performance

The assessment was the same as those used in multiple-treatment studies. The average of the relative percentage bias of the effect size was used to evaluate the bias of the effect size. The average of the relative percentage bias of the sampling variances and covariances was used to assess the bias of the sampling covariance matrices. In the heat maps, 5 and 20% were used as the cutoff points.

Similar to the simulation studies in the multiple-treatment studies, we followed the advice of one reviewer by displaying the results of the individual effect sizes. The results are shown in Supplementary Materials 3. We also reran the simulation by introducing unbalanced sample sizes. The levels of the sample sizes in the control and treatment groups were (100, 30), (100, 50), (30, 100), (30, 50), (50, 100), and (50, 100). The results are displayed in Supplementary Materials 4.

#### Results

**Figure 7:**
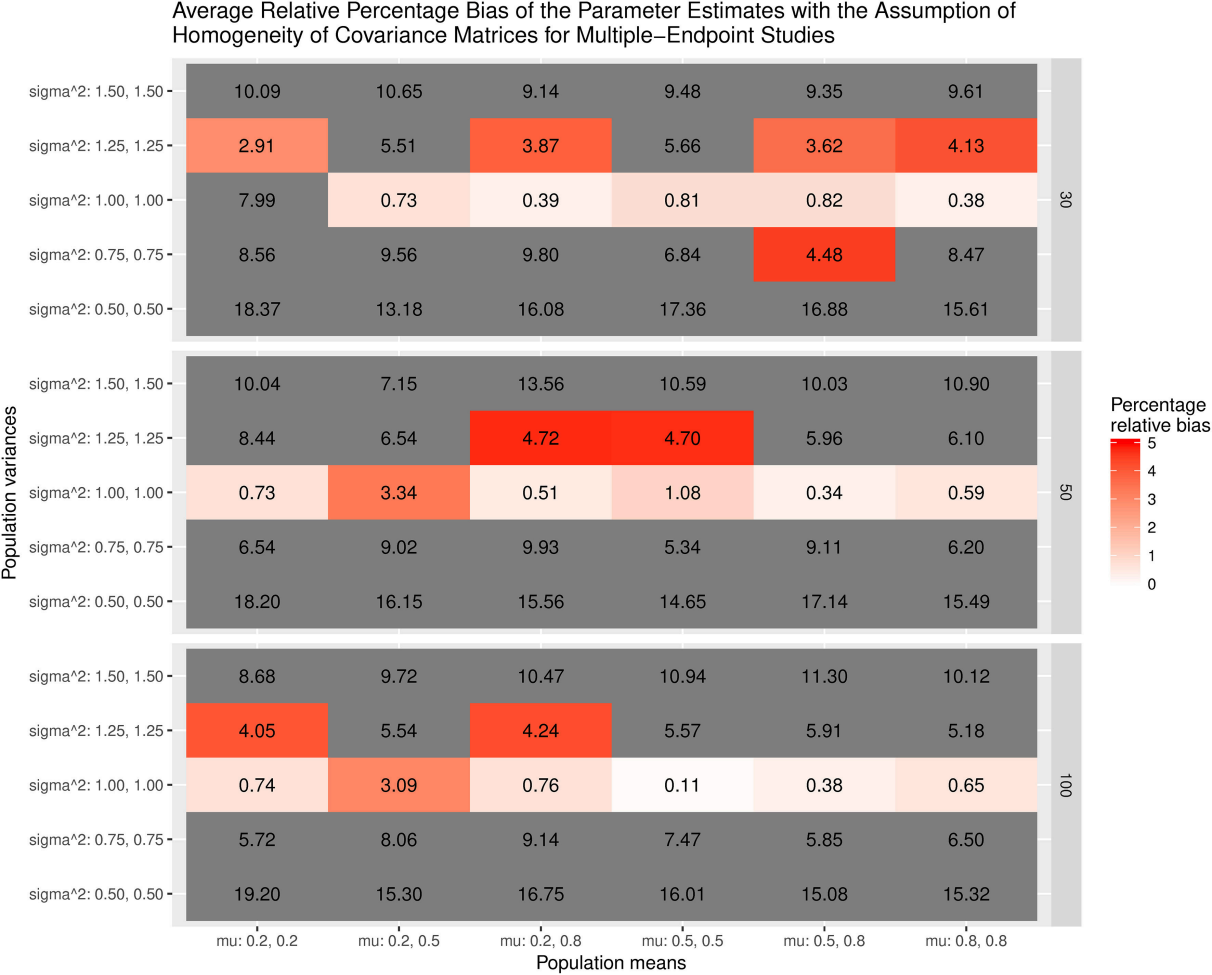
Relative bias of the average of the parameter estimates for the multiple-endpoint studies with the assumption of homogeneity of covariance matrices.

**Figure 8:**
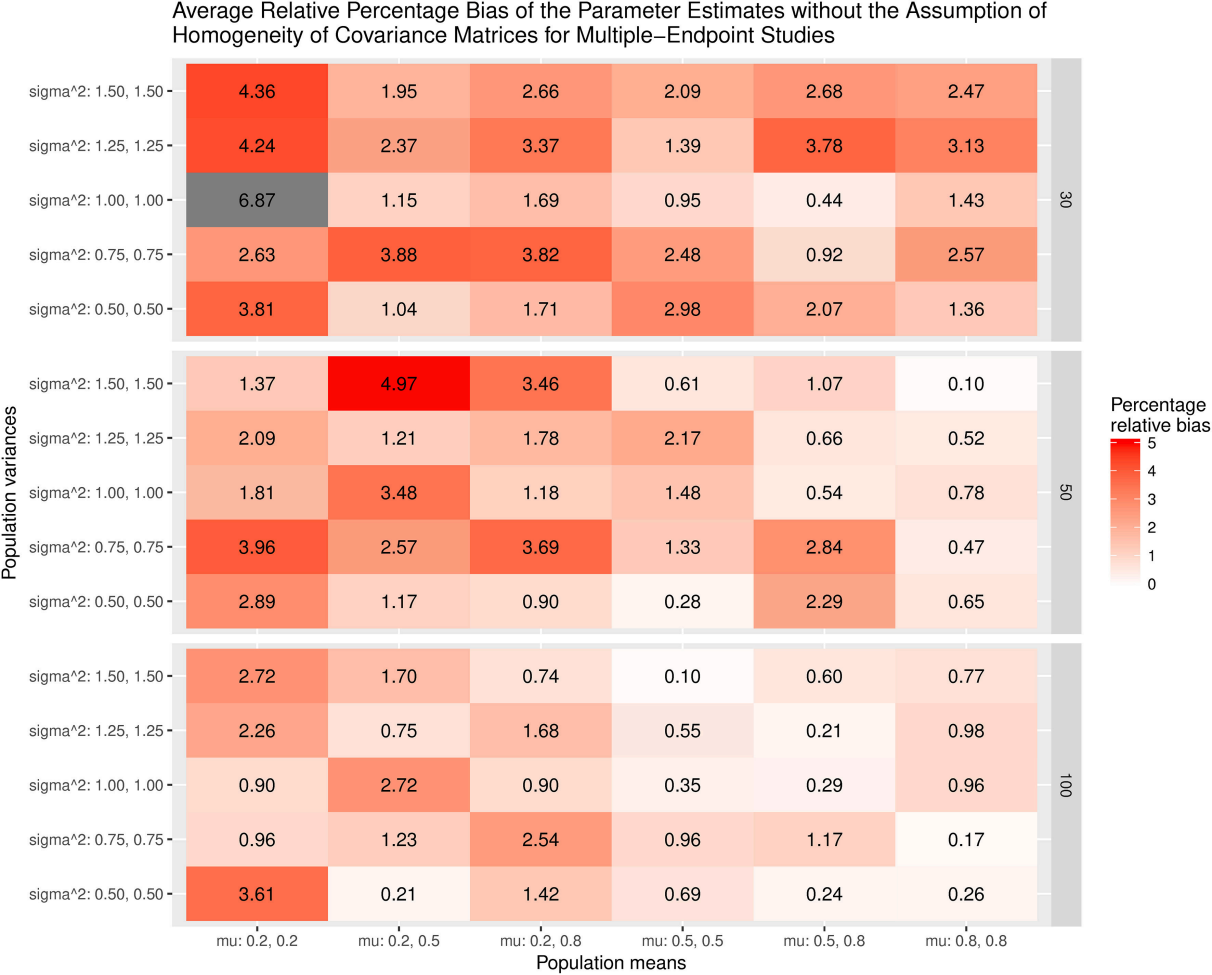
Relative bias of the average of parameter estimates for the multiple-endpoint studies without the assumption of homogeneity of covariance matrices.

Figure 7 displays the average bias of the effect sizes when we assume the homogeneity of covariance matrices in calculating the effect sizes. When the covariance matrices are homogeneous (sigma∧2 = 1.00, 1.00), the effect sizes are generally unbiased except when mu = 0.2, 0.2 and the sample size = 30. However, the effect sizes are always biased when the covariance matrices are not homogeneous. The most substantial relative bias can be up to 19%. This is expected because the variances of the treatment groups are very different from those of the control groups. Figure 8 shows the average bias of the effect sizes when we do not assume the homogeneity of covariance matrices when calculating the effect sizes. The effect sizes are generally unbiased except when mu = 0.2, 0.2 and the sample size = 30.

**Figure 9:**
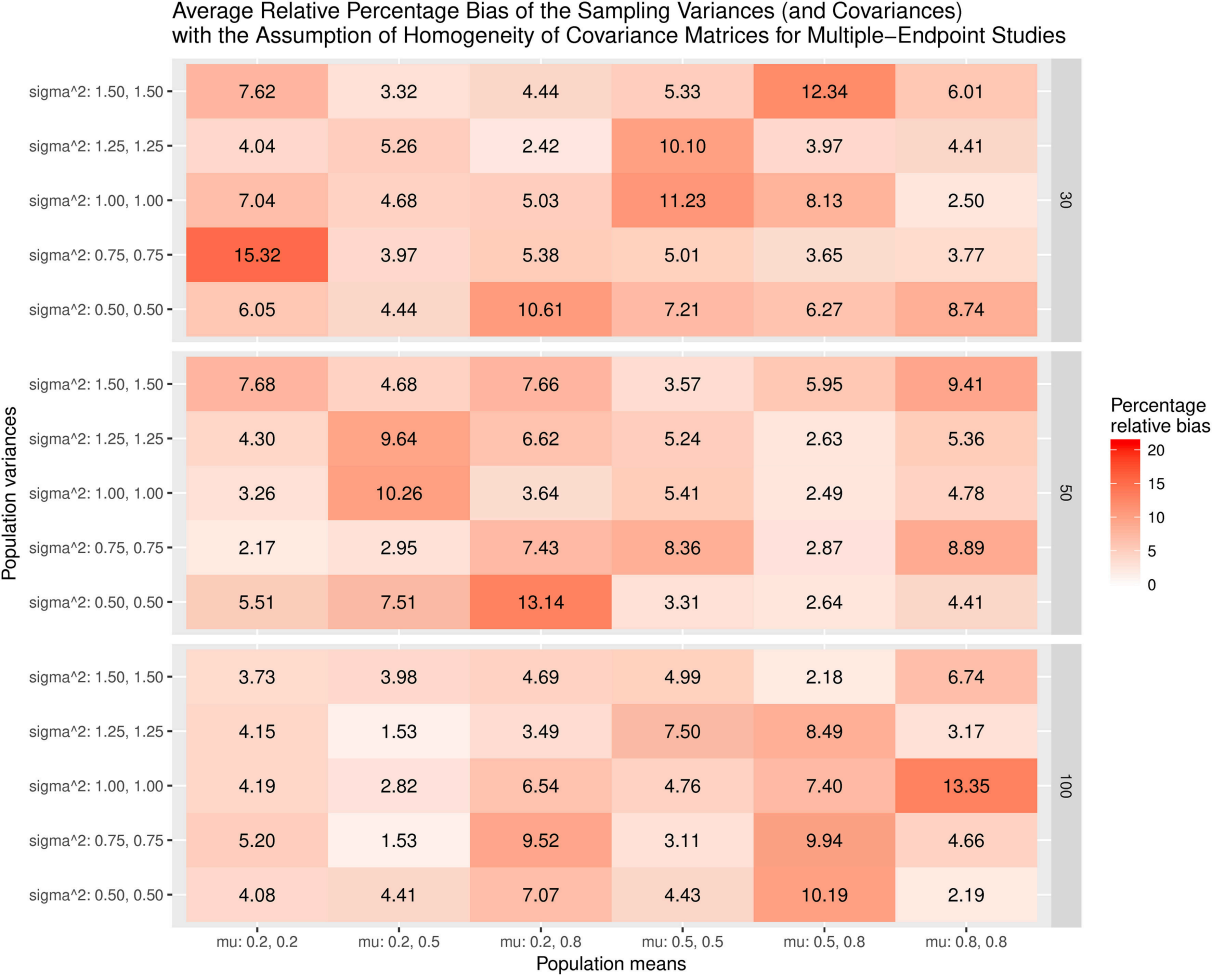
Relative bias of the average of the sampling variances and covariance for the multiple-endpoint studies with the assumption of homogeneity of covariance matrices.

**Figure 10:**
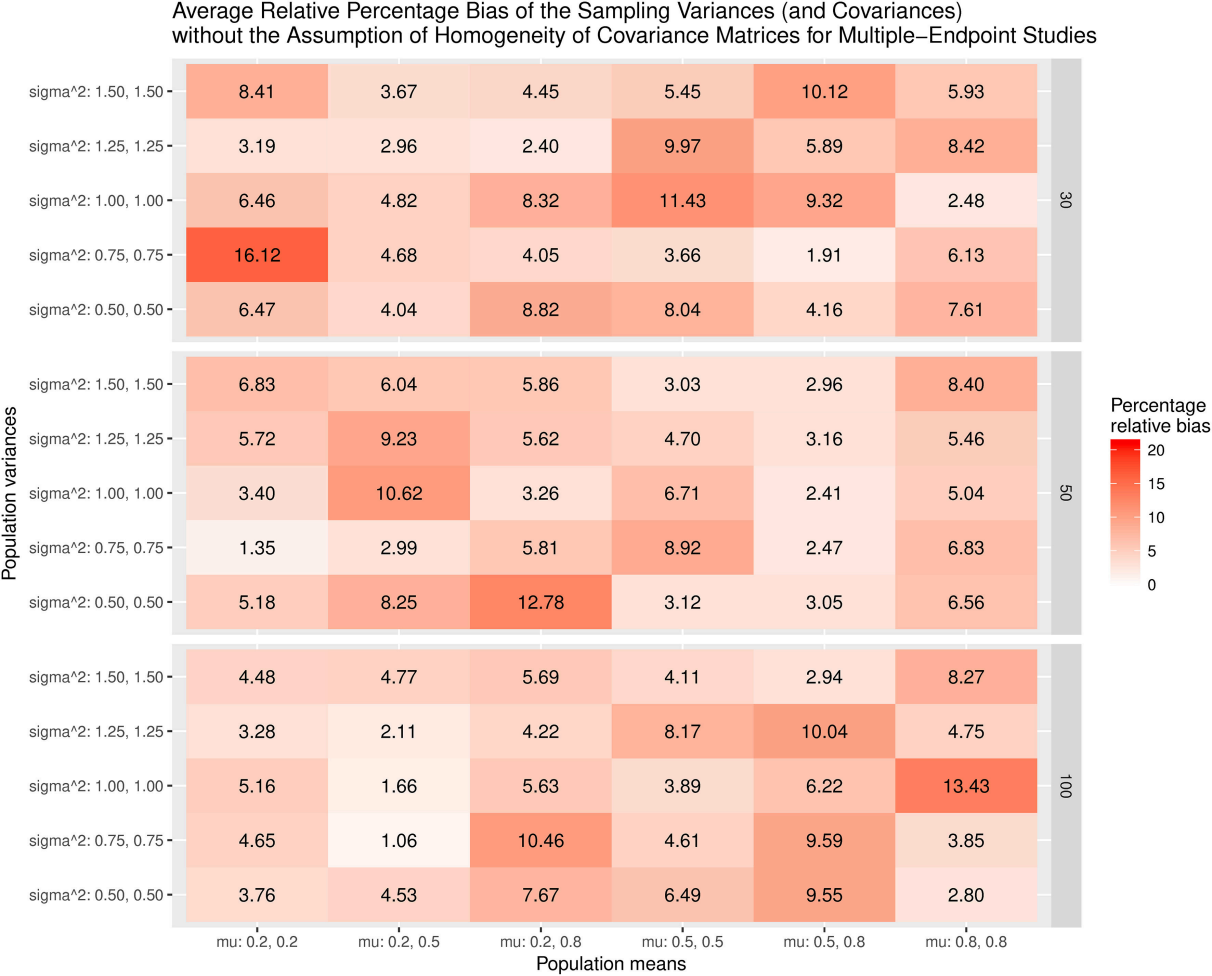
Relative bias of the average of the sampling variances and covariance for the multiple-endpoint studies without the assumption of homogeneity of covariance matrices.

Figure 9 displays the relative bias of the sampling variances and covariances when the effect sizes are estimated with the assumption of the homogeneity of covariance matrices. The bias is all below 20%. However, it should be noted that the effect sizes are biased. Thus, the results are still misleading. Figure 10 shows the relative bias of the sampling variances and covariances when the effect sizes are estimated without the assumption of the homogeneity of covariance matrices. As can be seen, they are generally unbiased.

The patterns of the individual parameters displayed in Supplementary Materials 3 are similar to those of the average parameters; therefore, we do not reproduce them here. Regarding the unbalanced data, the patterns are similar to those in multiple-treatment studies. The bias of the estimated effect sizes with the homogeneity assumption is much larger than that for the balanced data. On the other hand, the impact of the unbalanced sample sizes on the estimated effect sizes without the assumption of homogeneity is minimal.

To summarize, the estimated effect sizes are quite sensitive to the assumption of the homogeneity of covariance matrices. If the data are not homogeneous in covariance matrices and we incorrectly assume that they are, the estimated effect sizes are likely to be biased. On the other hand, the sampling covariance matrices are generally similar regardless of whether or not we have imposed the assumption of the homogeneity of covariance matrices.

## Conclusion

This study shows that multivariate effect sizes for multiple-treatment and multiple-endpoint studies can easily be obtained using the SEM approach. Researchers may impose equality constraints on the variances and covariances, and the SEM packages will report the effect sizes and their sampling covariance matrices.

For multiple-treatment studies, the estimated effect sizes are unbiased regardless of whether or not we assume that the variances are homogeneous when calculating the effect sizes when the common *SD*s are close to the *SD*s of the control group. We may expect that there will be substantial bias when the common *SD*s are different from the *SD*s of the control group. Moreover, the estimated sampling covariance matrices are biased when the variances are heterogeneous, but we incorrectly assume that the variances are homogeneous.

For multiple-endpoint studies, the estimated effect sizes are biased when the covariance matrices are different, but we mistakenly assume that the covariance matrices are homogeneous. On the other hand, the sample covariance matrices are similar regardless of whether or not we have imposed the assumption of the homogeneity of covariance matrices when estimating the effect sizes.

The findings indicate that researchers should always check the assumptions before calculating the effect sizes. Researchers may also check the robustness of the findings by dropping these assumptions. By comparing the results with and without the assumption of the homogeneity of variances or covariance matrices, researchers may have a better idea of whether their substantive findings depend on these assumptions. Based on the simulation studies, it can be seen that the results are similar for the approaches with and without the assumption of the homogeneity of variances (or covariance matrices) when the data actually have the same variances (or covariance matrices). Therefore, the loss of efficiency from dropping the assumption of the homogeneity of variances (or covariance matrices) is small.

It should be noted that only a few factors were studied in the simulation studies. Further simulation studies may address the question of whether the findings are consistent in other conditions such as in those of unbalanced data and data with non-normal distributions. Another possible direction of research is to study how the assumption of the homogeneity of variances or covariance matrices impacts the actual parameter estimates in a meta-analysis. Such a study may provide stronger evidence to guide researchers on the issue of whether or not to report effect sizes with the assumption of homogeneity.

To conclude, it seems reasonable not to assume the homogeneity of variances (or covariance matrices) when calculating effect sizes for multiple-treatment and multiple-endpoint studies. The SEM approach provides a convenient device to calculate these effect sizes.

## Author contributions

The author confirms being the sole contributor of this work and approved it for publication.

### Conflict of interest statement

The author declares that the research was conducted in the absence of any commercial or financial relationships that could be construed as a potential conflict of interest. The reviewer, JP, declared a past co-authorship with the author, MC, to the handling Editor.

## Footnotes

^1^https://stats.stackexchange.com/questions/108248/calculating-effect-sizes-and-standard-errors-for-the-difference-between-two-stan/130512.
