# Supplementary material for: Computing Multivariate Effect Sizes and Their Sampling Covariance Matrices With Structural Equation Modeling: Theory, Examples, and Computer Simulations
Source: Front Psychol. 2018 Aug 17;9:1387. doi: 10.3389/fpsyg.2018.01387 (PMC6107852; doi:10.3389/fpsyg.2018.01387)

Relative Percentage Bias of the First Parameter Estimates with the Assumption of Homogeneity of Variances for Multiple Treatment Studies

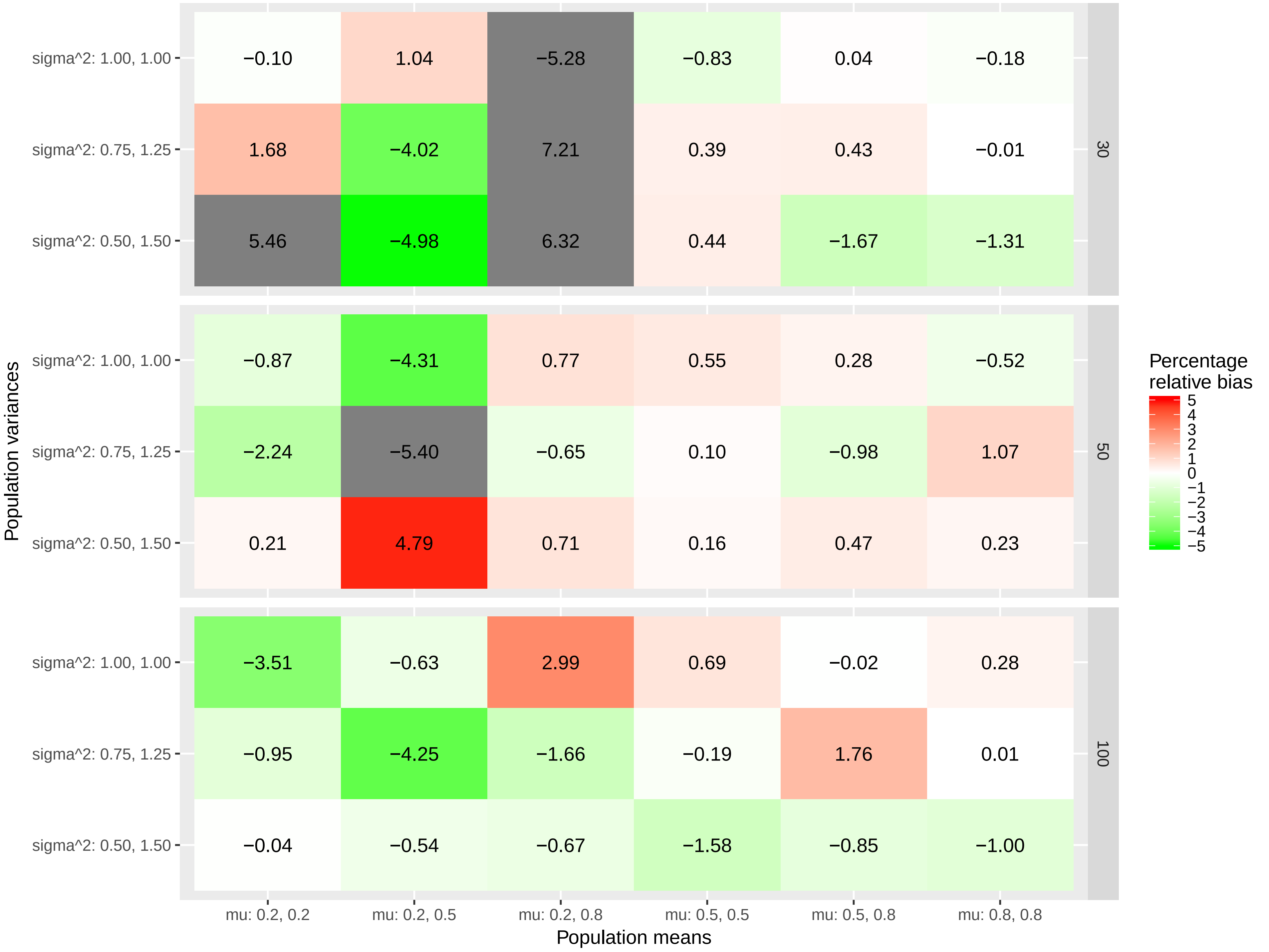

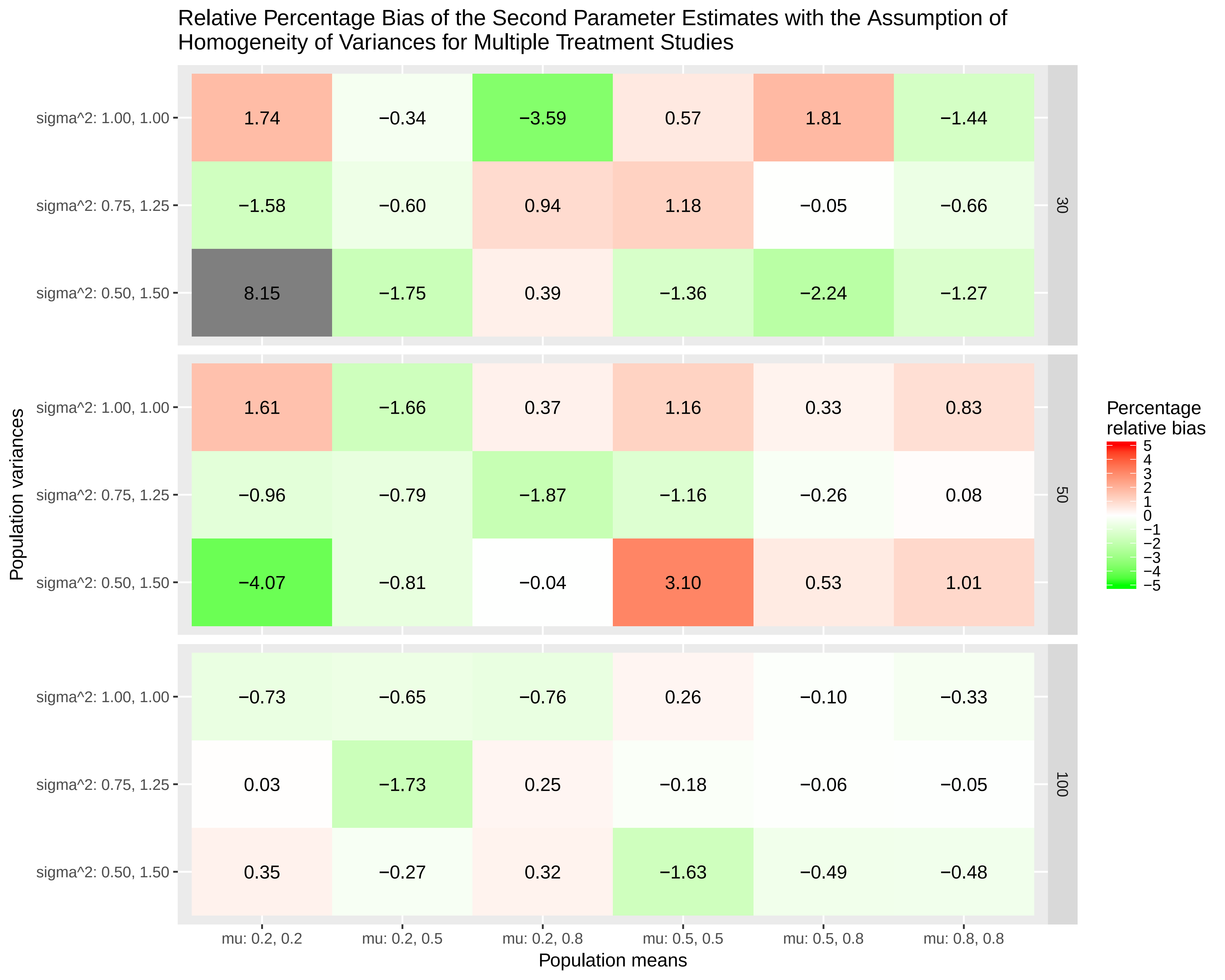
Relative Percentage Bias of the Second Parameter Estimates with the Assumption of Homogeneity of Variances for Multiple Treatment Studies
Population variances
sigma^2: 1.00, 1.00
sigma^2: 0.75, 1.25
sigma^2: 0.50, 1.50
30
1.74 −0.34 −3.59 0.57 1.81 −1.44
−1.58 −0.60 0.94 1.18 −0.05 −0.66
8.15 −1.75 0.39 −1.36 −2.24 −1.27
50
1.61 −1.66 0.37 1.16 0.33 0.83
−0.96 −0.79 −1.87 −1.16 −0.26 0.08
−4.07 −0.81 −0.04 3.10 0.53 1.01
100
−0.73 −0.65 −0.76 0.26 −0.10 −0.33
0.03 −1.73 0.25 −0.18 −0.06 −0.05
0.35 −0.27 0.32 −1.63 −0.49 −0.48
mu: 0.2, 0.2
mu: 0.2, 0.5
mu: 0.2, 0.8
mu: 0.5, 0.5
mu: 0.5, 0.8
mu: 0.8, 0.8
Population means
Percentage relative bias
5 4 3 2 1 0 −1 −2 −3 −4 −5

Relative Percentage Bias of the First Parameter Estimates without the Assumption of Homogeneity of Variances for Multiple Treatment Studies

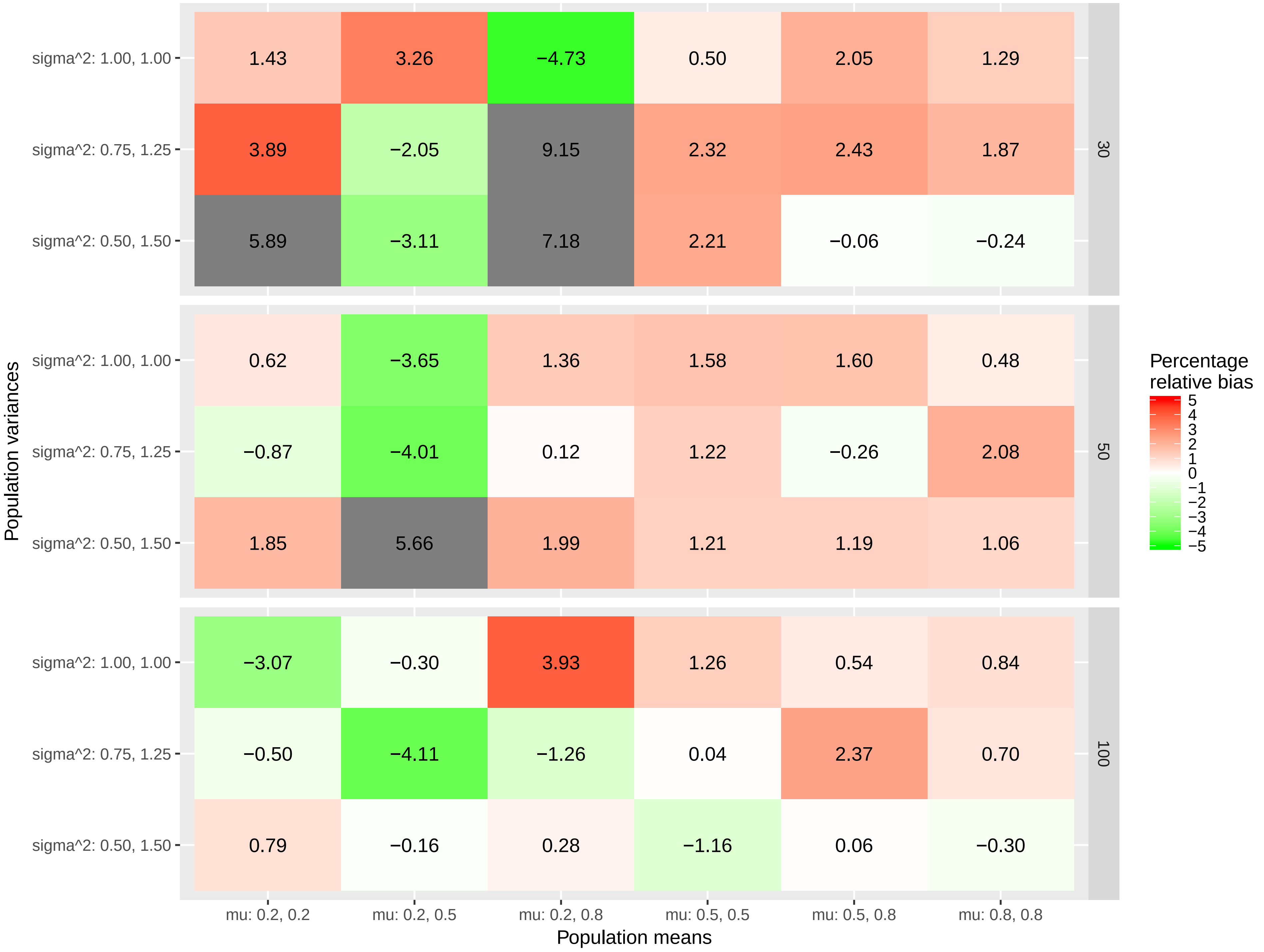

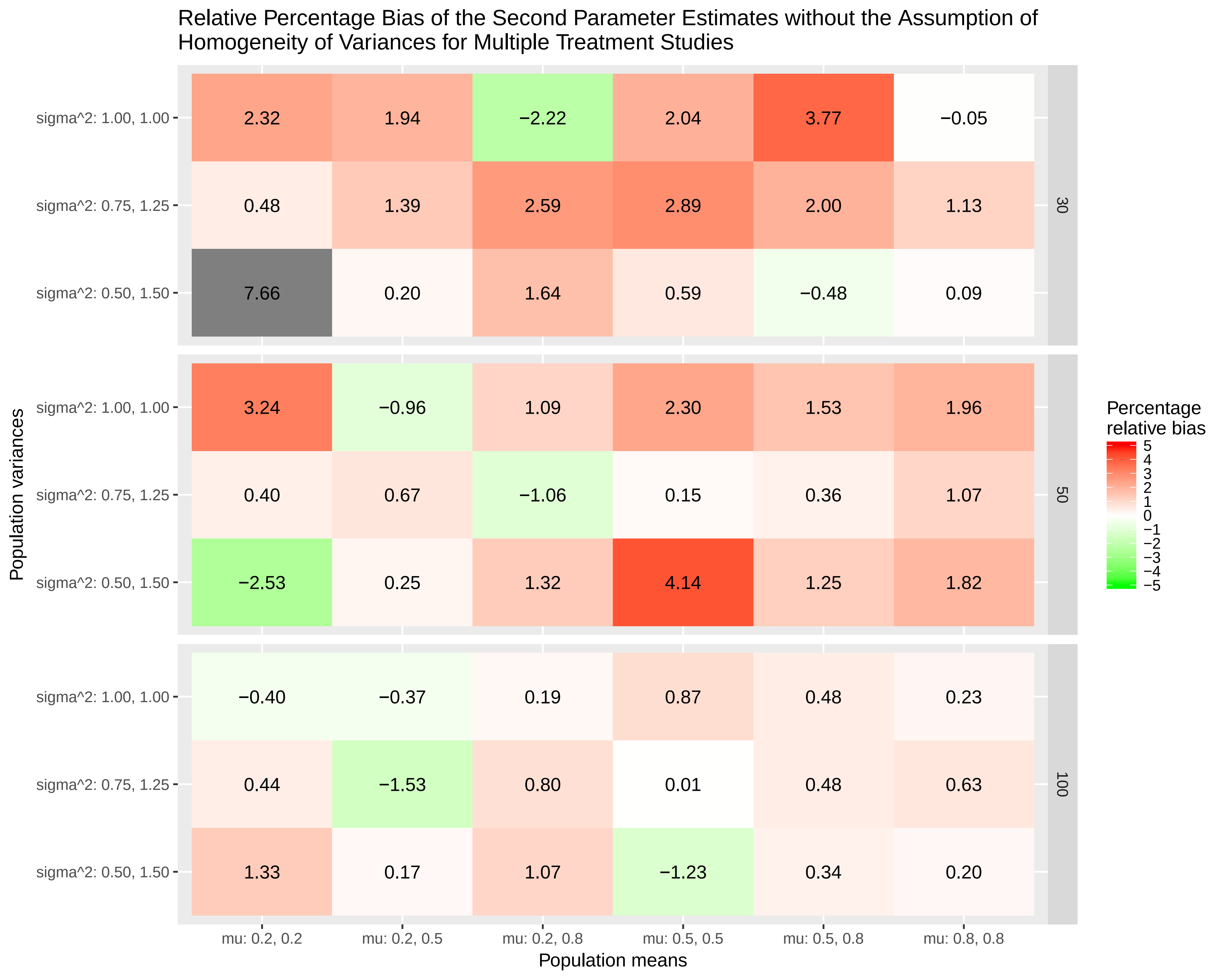

Relative Percentage Bias of the Second Parameter Estimates without the Assumption of Homogeneity of Variances for Multiple Treatment Studies
Population variances
Population means
sigma^2: 1.00, 1.00
sigma^2: 0.75, 1.25
sigma^2: 0.50, 1.50
mu: 0.2, 0.2
mu: 0.2, 0.5
mu: 0.2, 0.8
mu: 0.5, 0.5
mu: 0.5, 0.8
mu: 0.8, 0.8
30
50
100
2.32
1.94
−2.22
2.04
3.77
−0.05
0.48
1.39
2.59
2.89
2.00
1.13
7.66
0.20
1.64
0.59
−0.48
0.09
3.24
−0.96
1.09
2.30
1.53
1.96
0.40
0.67
−1.06
0.15
0.36
1.07
−2.53
0.25
1.32
4.14
1.25
1.82
−0.40
−0.37
0.19
0.87
0.48
0.23
0.44
−1.53
0.80
0.01
0.48
0.63
1.33
0.17
1.07
−1.23
0.34
0.20
Percentage relative bias
5
4
3
2
1
0
−1
−2
−3
−4
−5

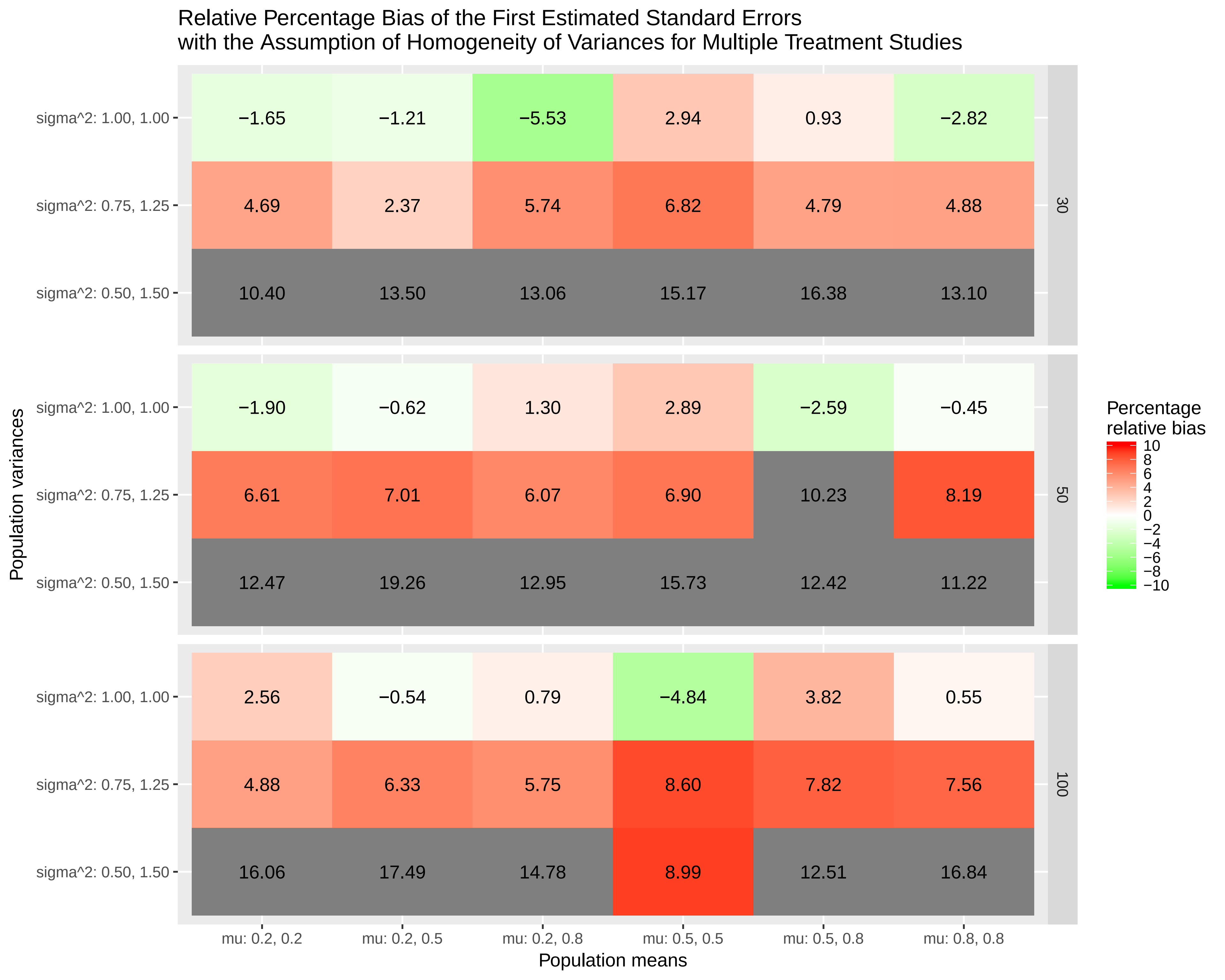

Relative Percentage Bias of the First Estimated Standard Errors
with the Assumption of Homogeneity of Variances for Multiple Treatment Studies
Population variances
Population means
sigma^2: 1.00, 1.00
sigma^2: 0.75, 1.25
sigma^2: 0.50, 1.50
mu: 0.2, 0.2
mu: 0.2, 0.5
mu: 0.2, 0.8
mu: 0.5, 0.5
mu: 0.5, 0.8
mu: 0.8, 0.8
30
50
100
−1.65 −1.21 −5.53 2.94 0.93 −2.82
4.69 2.37 5.74 6.82 4.79 4.88
10.40 13.50 13.06 15.17 16.38 13.10
−1.90 −0.62 1.30 2.89 −2.59 −0.45
6.61 7.01 6.07 6.90 10.23 8.19
12.47 19.26 12.95 15.73 12.42 11.22
2.56 −0.54 0.79 −4.84 3.82 0.55
4.88 6.33 5.75 8.60 7.82 7.56
16.06 17.49 14.78 8.99 12.51 16.84
Percentage
relative bias
10
8
6
4
2
0
−2
−4
−6
−8
−10

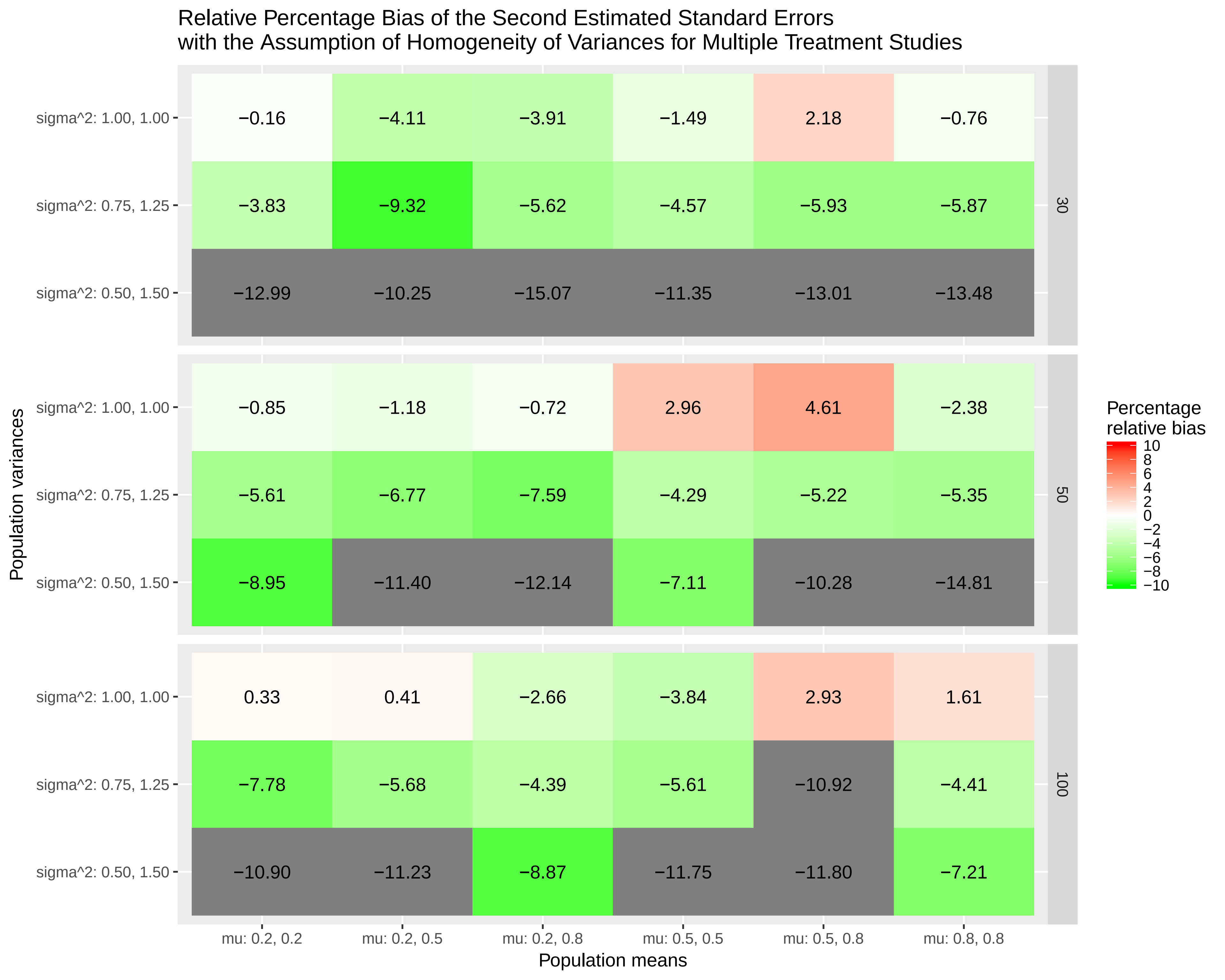
Relative Percentage Bias of the Second Estimated Standard Errors
with the Assumption of Homogeneity of Variances for Multiple Treatment Studies
Population variances
Population means
sigma^2: 1.00, 1.00
sigma^2: 0.75, 1.25
sigma^2: 0.50, 1.50
mu: 0.2, 0.2
mu: 0.2, 0.5
mu: 0.2, 0.8
mu: 0.5, 0.5
mu: 0.5, 0.8
mu: 0.8, 0.8
30
50
100
−0.16 −4.11 −3.91 −1.49 2.18 −0.76
−3.83 −9.32 −5.62 −4.57 −5.93 −5.87
−12.99 −10.25 −15.07 −11.35 −13.01 −13.48
−0.85 −1.18 −0.72 2.96 4.61 −2.38
−5.61 −6.77 −7.59 −4.29 −5.22 −5.35
−8.95 −11.40 −12.14 −7.11 −10.28 −14.81
0.33 0.41 −2.66 −3.84 2.93 1.61
−7.78 −5.68 −4.39 −5.61 −10.92 −4.41
−10.90 −11.23 −8.87 −11.75 −11.80 −7.21
Percentage
relative bias
10
8
6
4
2
0
−2
−4
−6
−8
−10

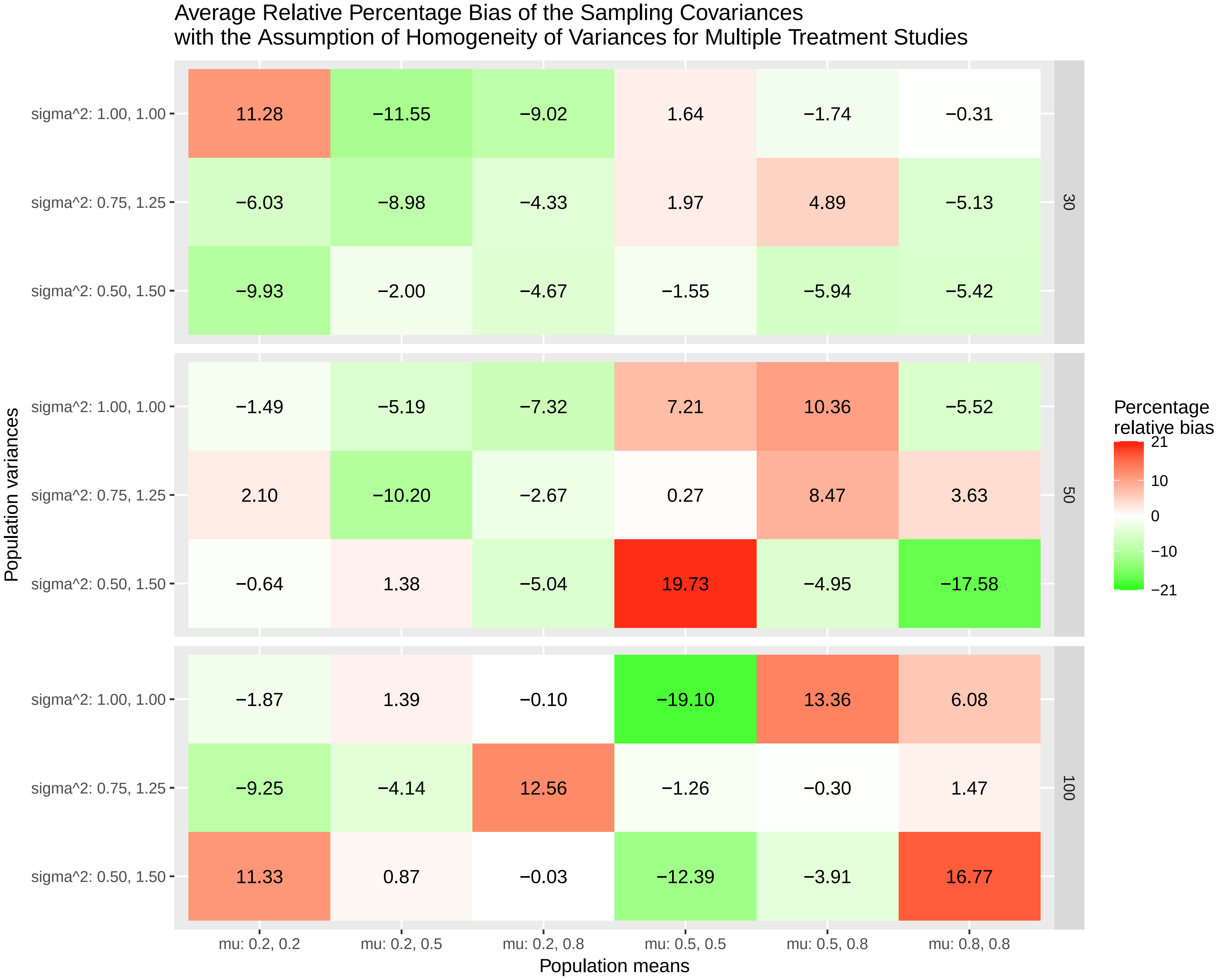
Average Relative Percentage Bias of the Sampling Covariances
with the Assumption of Homogeneity of Variances for Multiple Treatment Studies
Population variances
sigma^2: 1.00, 1.00
sigma^2: 0.75, 1.25
sigma^2: 0.50, 1.50
30
11.28
−11.55
−9.02
1.64
−1.74
−0.31
−6.03
−8.98
−4.33
1.97
4.89
−5.13
−9.93
−2.00
−4.67
−1.55
−5.94
−5.42
50
−1.49
−5.19
−7.32
7.21
10.36
−5.52
2.10
−10.20
−2.67
0.27
8.47
3.63
−0.64
1.38
−5.04
19.73
−4.95
−17.58
100
−1.87
1.39
−0.10
−19.10
13.36
6.08
−9.25
−4.14
12.56
−1.26
−0.30
1.47
11.33
0.87
−0.03
−12.39
−3.91
16.77
mu: 0.2, 0.2
mu: 0.2, 0.5
mu: 0.2, 0.8
mu: 0.5, 0.5
mu: 0.5, 0.8
mu: 0.8, 0.8
Population means
Percentage
relative bias
21
10
0
−10
−21

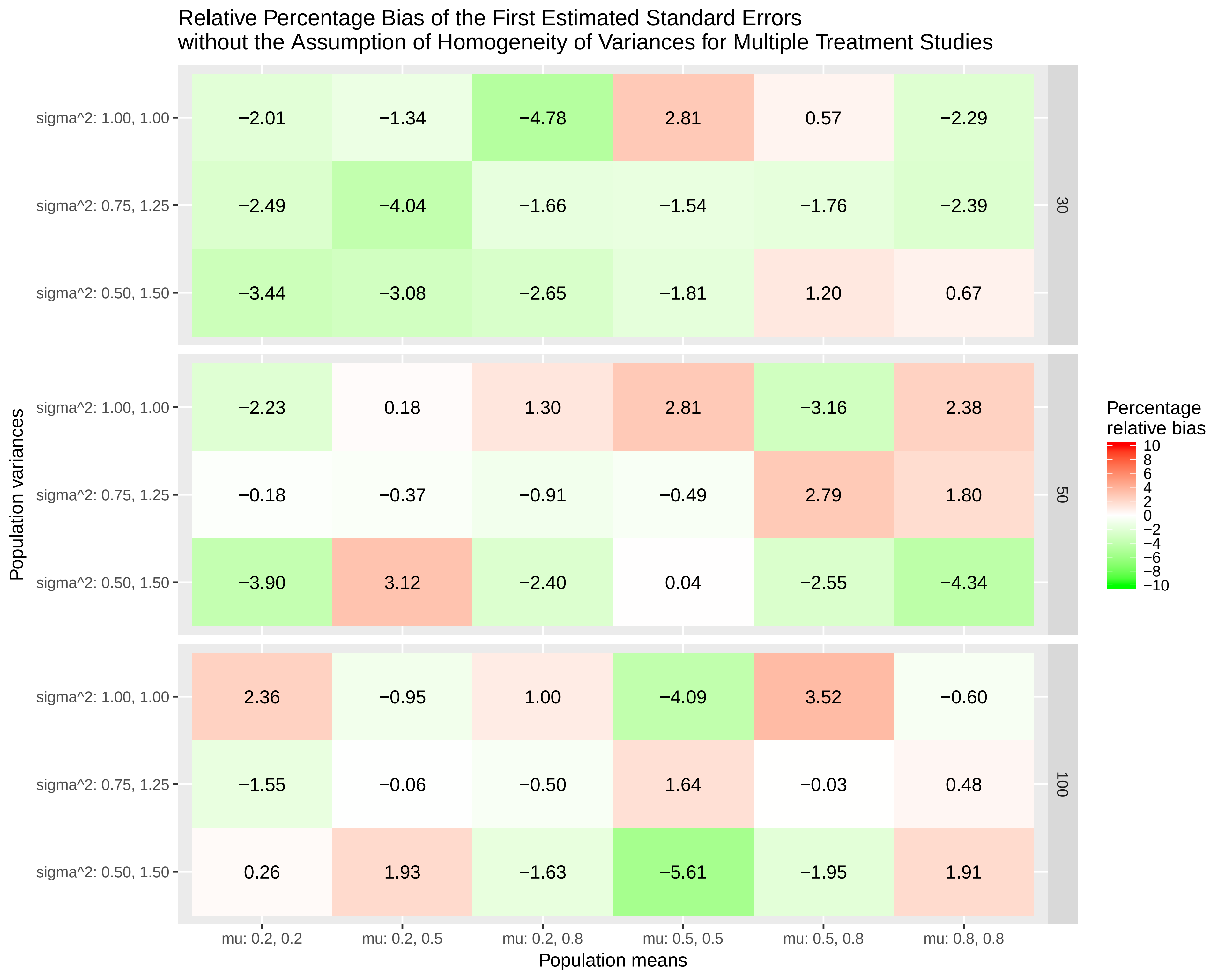

Relative Percentage Bias of the First Estimated Standard Errors
without the Assumption of Homogeneity of Variances for Multiple Treatment Studies
Population variances
Population means
sigma^2: 1.00, 1.00
sigma^2: 0.75, 1.25
sigma^2: 0.50, 1.50
mu: 0.2, 0.2
mu: 0.2, 0.5
mu: 0.2, 0.8
mu: 0.5, 0.5
mu: 0.5, 0.8
mu: 0.8, 0.8
30
50
100
Percentage relative bias
−2.01 −1.34 −4.78 2.81 0.57 −2.29
−2.49 −4.04 −1.66 −1.54 −1.76 −2.39
−3.44 −3.08 −2.65 −1.81 1.20 0.67
−2.23 0.18 1.30 2.81 −3.16 2.38
−0.18 −0.37 −0.91 −0.49 2.79 1.80
−3.90 3.12 −2.40 0.04 −2.55 −4.34
2.36 −0.95 1.00 −4.09 3.52 −0.60
−1.55 −0.06 −0.50 1.64 −0.03 0.48
0.26 1.93 −1.63 −5.61 −1.95 1.91

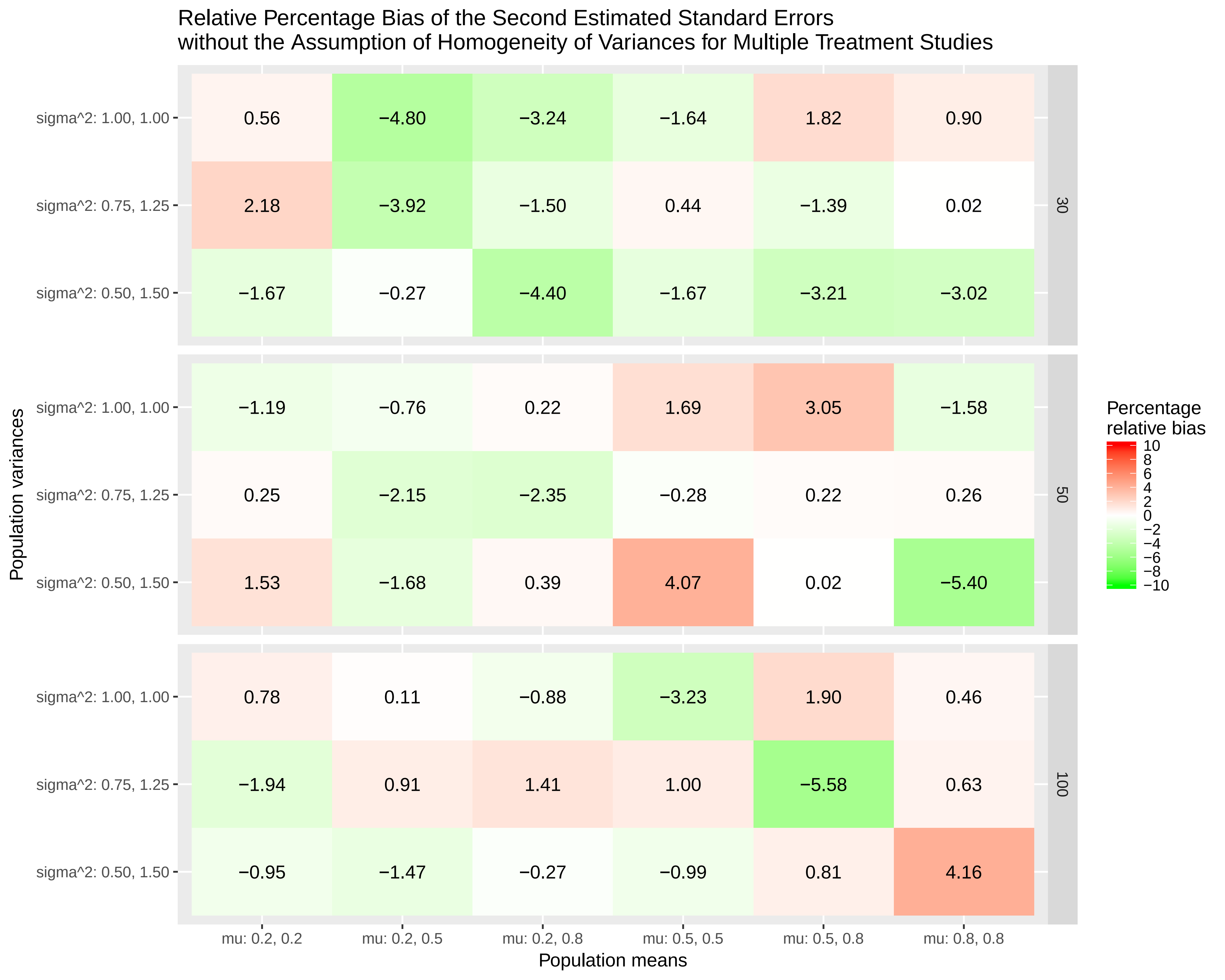
Relative Percentage Bias of the Second Estimated Standard Errors
without the Assumption of Homogeneity of Variances for Multiple Treatment Studies
Population variances
Population means
sigma^2: 1.00, 1.00
sigma^2: 0.75, 1.25
sigma^2: 0.50, 1.50
mu: 0.2, 0.2
mu: 0.2, 0.5
mu: 0.2, 0.8
mu: 0.5, 0.5
mu: 0.5, 0.8
mu: 0.8, 0.8
30
0.56 −4.80 −3.24 −1.64 1.82 0.90
2.18 −3.92 −1.50 0.44 −1.39 0.02
−1.67 −0.27 −4.40 −1.67 −3.21 −3.02
50
−1.19 −0.76 0.22 1.69 3.05 −1.58
0.25 −2.15 −2.35 −0.28 0.22 0.26
1.53 −1.68 0.39 4.07 0.02 −5.40
100
0.78 0.11 −0.88 −3.23 1.90 0.46
−1.94 0.91 1.41 1.00 −5.58 0.63
−0.95 −1.47 −0.27 −0.99 0.81 4.16
Percentage
relative bias
10
8
6
4
2
0
−2
−4
−6
−8
−10

Average Relative Percentage Bias of the Sampling Covariances
without the Assumption of Homogeneity of Variances for Multiple Treatment Studies

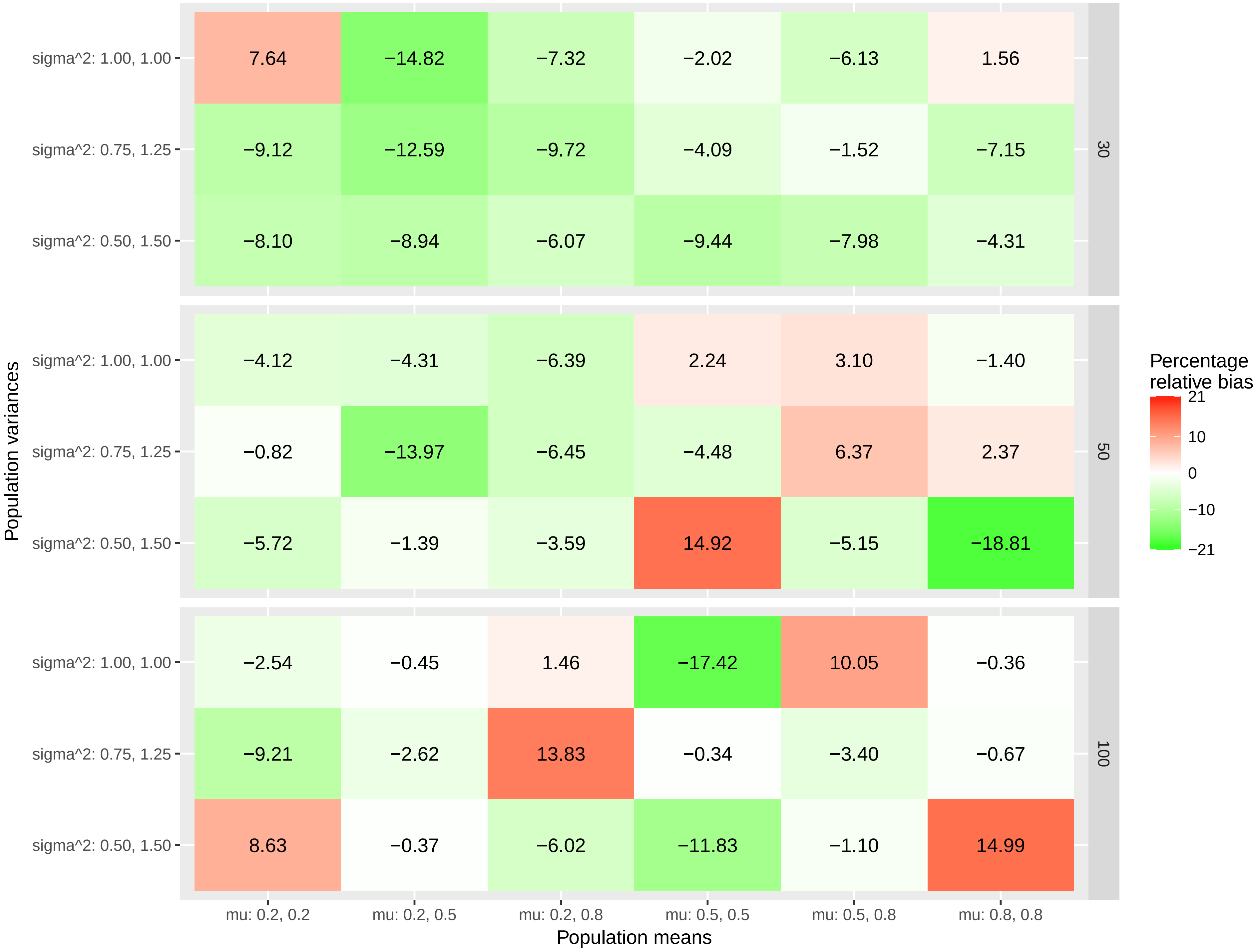

Supplement: Supplementary file 1 [file Data_Sheet_1.PDF]
