# Supplementary material for: Computing Multivariate Effect Sizes and Their Sampling Covariance Matrices With Structural Equation Modeling: Theory, Examples, and Computer Simulations
Source: Front Psychol. 2018 Aug 17;9:1387. doi: 10.3389/fpsyg.2018.01387 (PMC6107852; doi:10.3389/fpsyg.2018.01387)

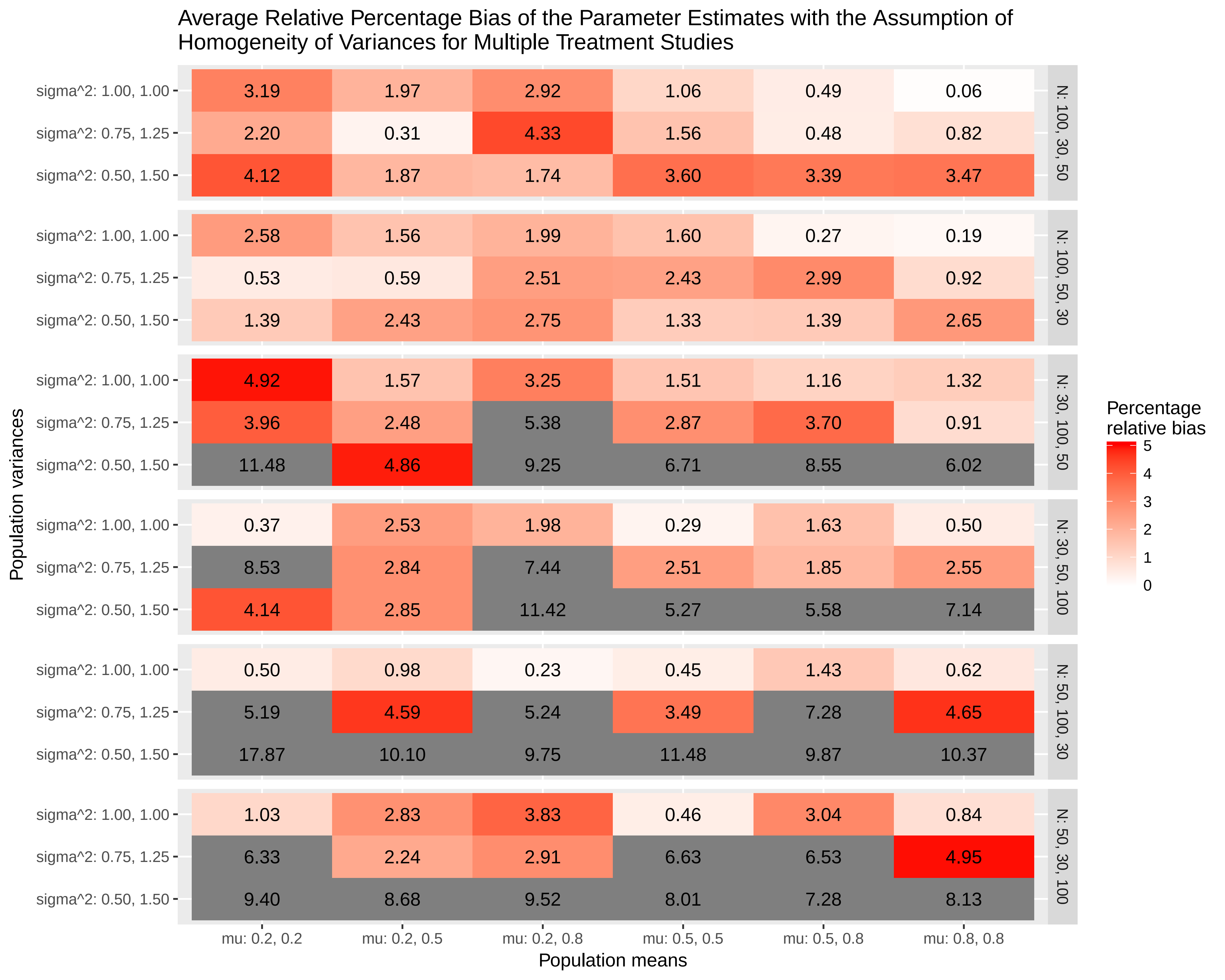
Average Relative Percentage Bias of the Parameter Estimates with the Assumption of
Homogeneity of Variances for Multiple Treatment Studies
Population variances
Population means
mu: 0.2, 0.2
mu: 0.2, 0.5
mu: 0.2, 0.8
mu: 0.5, 0.5
mu: 0.5, 0.8
mu: 0.8, 0.8
sigma^2: 1.00, 1.00
sigma^2: 0.75, 1.25
sigma^2: 0.50, 1.50
N: 100, 30, 50
3.19 1.97 2.92 1.06 0.49 0.06
2.20 0.31 4.33 1.56 0.48 0.82
4.12 1.87 1.74 3.60 3.39 3.47
N: 100, 50, 30
2.58 1.56 1.99 1.60 0.27 0.19
0.53 0.59 2.51 2.43 2.99 0.92
1.39 2.43 2.75 1.33 1.39 2.65
N: 30, 100, 50
4.92 1.57 3.25 1.51 1.16 1.32
3.96 2.48 5.38 2.87 3.70 0.91
11.48 4.86 9.25 6.71 8.55 6.02
N: 30, 50, 100
0.37 2.53 1.98 0.29 1.63 0.50
8.53 2.84 7.44 2.51 1.85 2.55
4.14 2.85 11.42 5.27 5.58 7.14
N: 50, 100, 30
0.50 0.98 0.23 0.45 1.43 0.62
5.19 4.59 5.24 3.49 7.28 4.65
17.87 10.10 9.75 11.48 9.87 10.37
N: 50, 30, 100
1.03 2.83 3.83 0.46 3.04 0.84
6.33 2.24 2.91 6.63 6.53 4.95
9.40 8.68 9.52 8.01 7.28 8.13
Percentage
relative bias
5
4
3
2
1
0

Average Relative Percentage Bias of the Parameter Estimates without the Assumption of Homogeneity of Variances for Multiple Treatment Studies

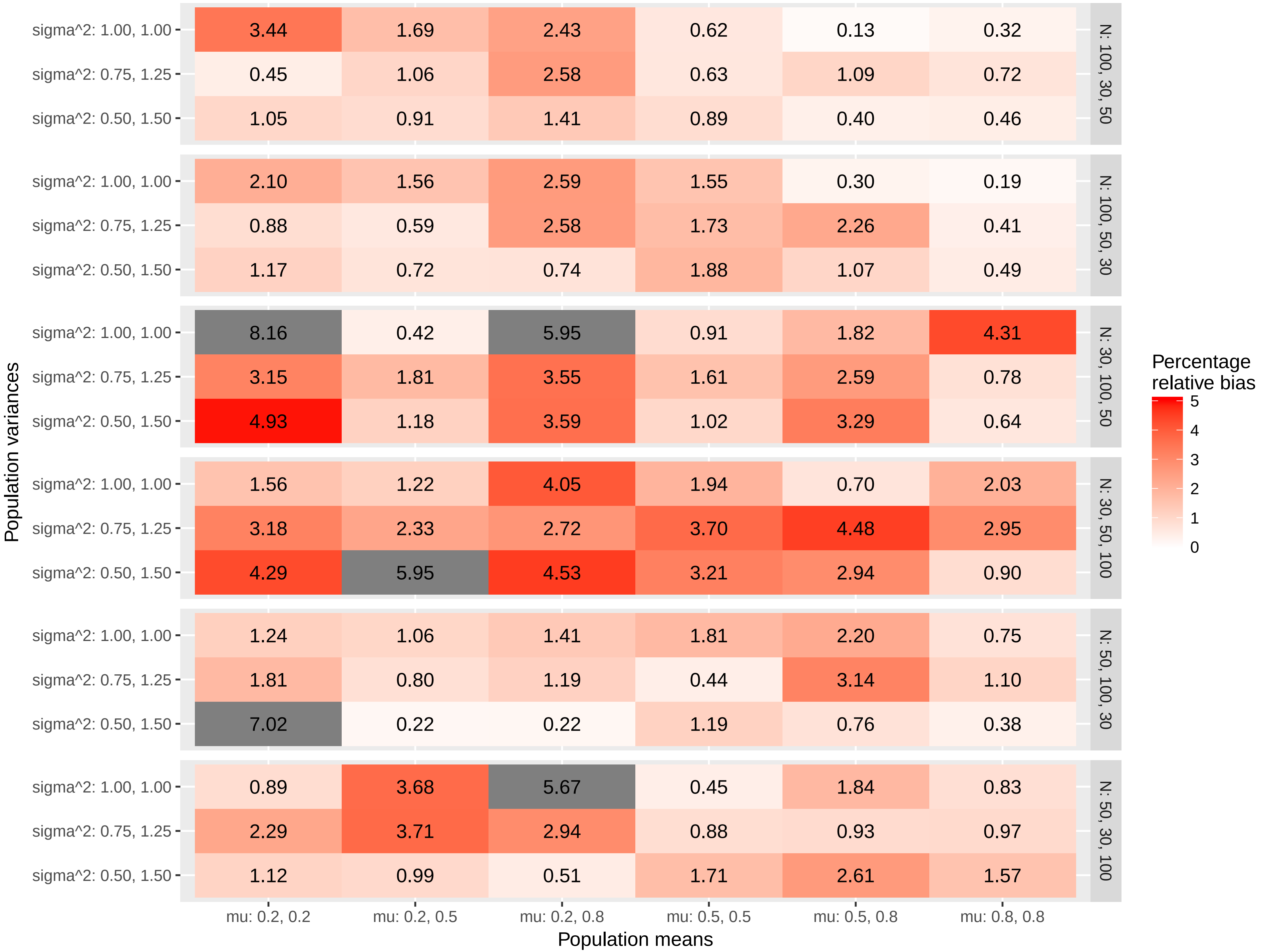

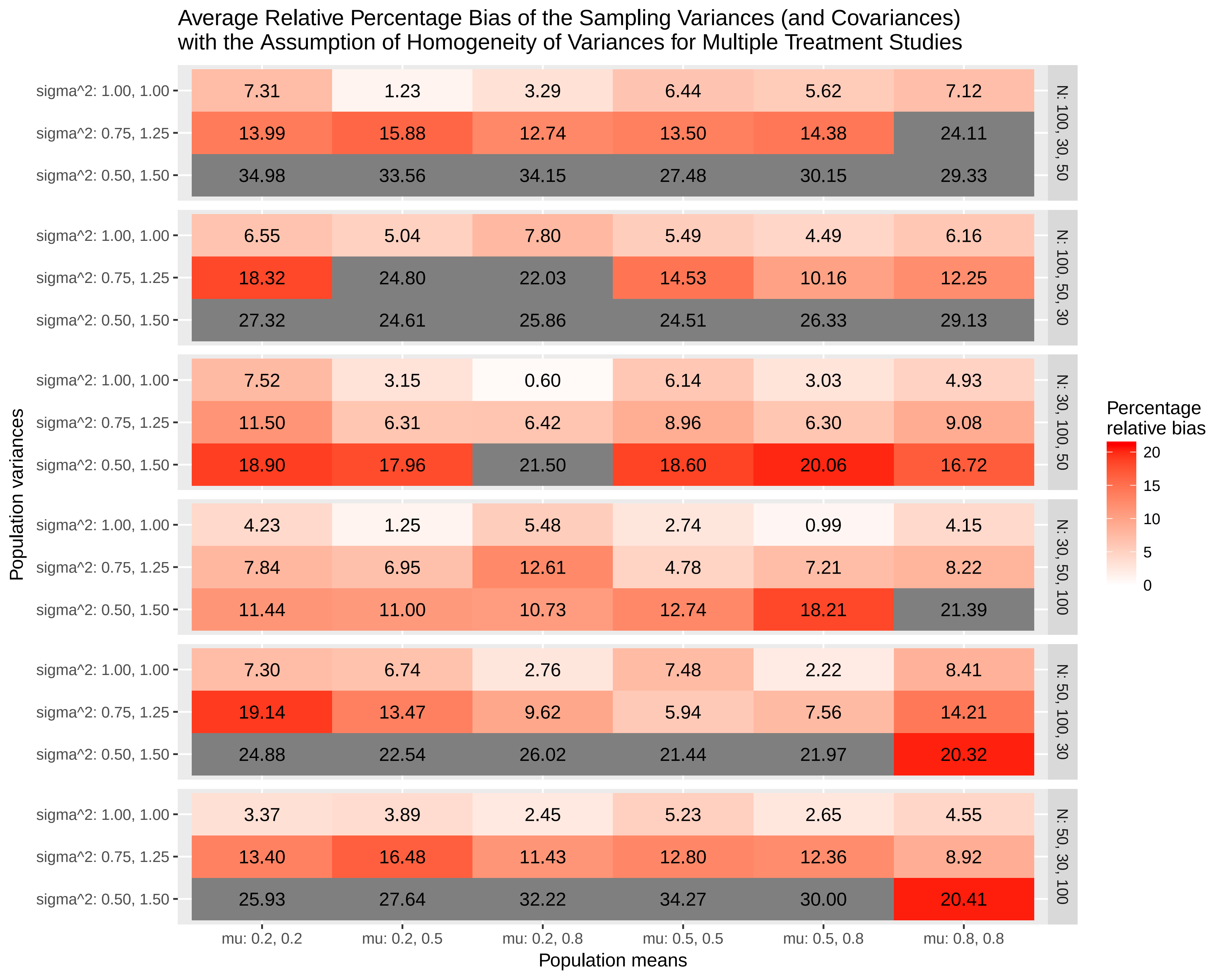
Average Relative Percentage Bias of the Sampling Variances (and Covariances)
with the Assumption of Homogeneity of Variances for Multiple Treatment Studies
Population variances
Population means
sigma^2: 1.00, 1.00
sigma^2: 0.75, 1.25
sigma^2: 0.50, 1.50
mu: 0.2, 0.2
mu: 0.2, 0.5
mu: 0.2, 0.8
mu: 0.5, 0.5
mu: 0.5, 0.8
mu: 0.8, 0.8
N: 100, 30, 50
7.31 1.23 3.29 6.44 5.62 7.12
13.99 15.88 12.74 13.50 14.38 24.11
34.98 33.56 34.15 27.48 30.15 29.33
N: 100, 50, 30
6.55 5.04 7.80 5.49 4.49 6.16
18.32 24.80 22.03 14.53 10.16 12.25
27.32 24.61 25.86 24.51 26.33 29.13
N: 30, 100, 50
7.52 3.15 0.60 6.14 3.03 4.93
11.50 6.31 6.42 8.96 6.30 9.08
18.90 17.96 21.50 18.60 20.06 16.72
N: 30, 50, 100
4.23 1.25 5.48 2.74 0.99 4.15
7.84 6.95 12.61 4.78 7.21 8.22
11.44 11.00 10.73 12.74 18.21 21.39
N: 50, 100, 30
7.30 6.74 2.76 7.48 2.22 8.41
19.14 13.47 9.62 5.94 7.56 14.21
24.88 22.54 26.02 21.44 21.97 20.32
N: 50, 30, 100
3.37 3.89 2.45 5.23 2.65 4.55
13.40 16.48 11.43 12.80 12.36 8.92
25.93 27.64 32.22 34.27 30.00 20.41
Percentage
relative bias
20
15
10
5
0

Average Relative Percentage Bias of the Sampling Variances (and Covariances)
without the Assumption of Homogeneity of Variances for Multiple Treatment Studies

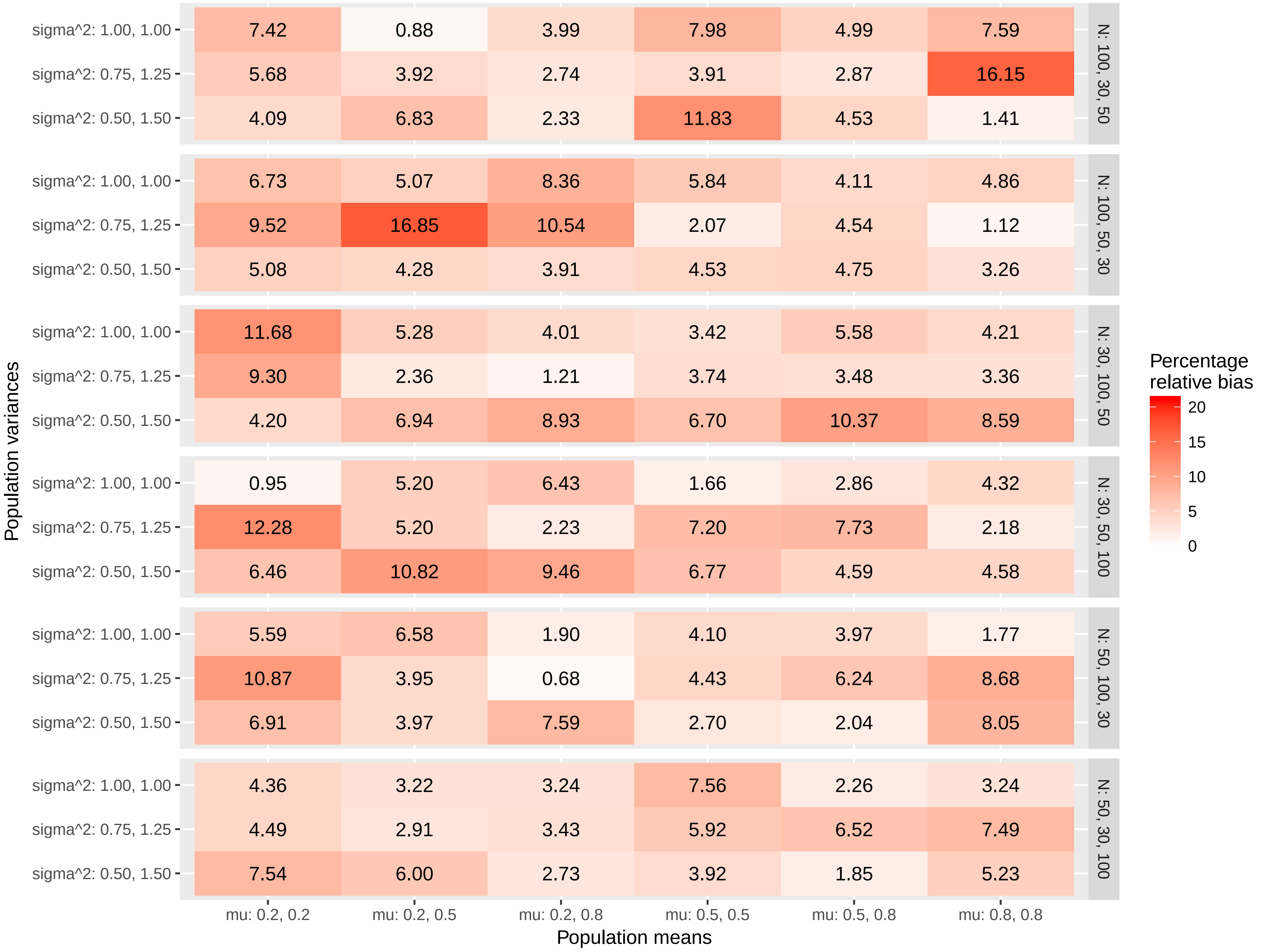

Supplement: Supplementary file 2 [file Data_Sheet_2.PDF]
