# Supplementary material for: Computing Multivariate Effect Sizes and Their Sampling Covariance Matrices With Structural Equation Modeling: Theory, Examples, and Computer Simulations
Source: Front Psychol. 2018 Aug 17;9:1387. doi: 10.3389/fpsyg.2018.01387 (PMC6107852; doi:10.3389/fpsyg.2018.01387)
Supplement: Supplementary file 3 [file Data_Sheet_3.PDF]

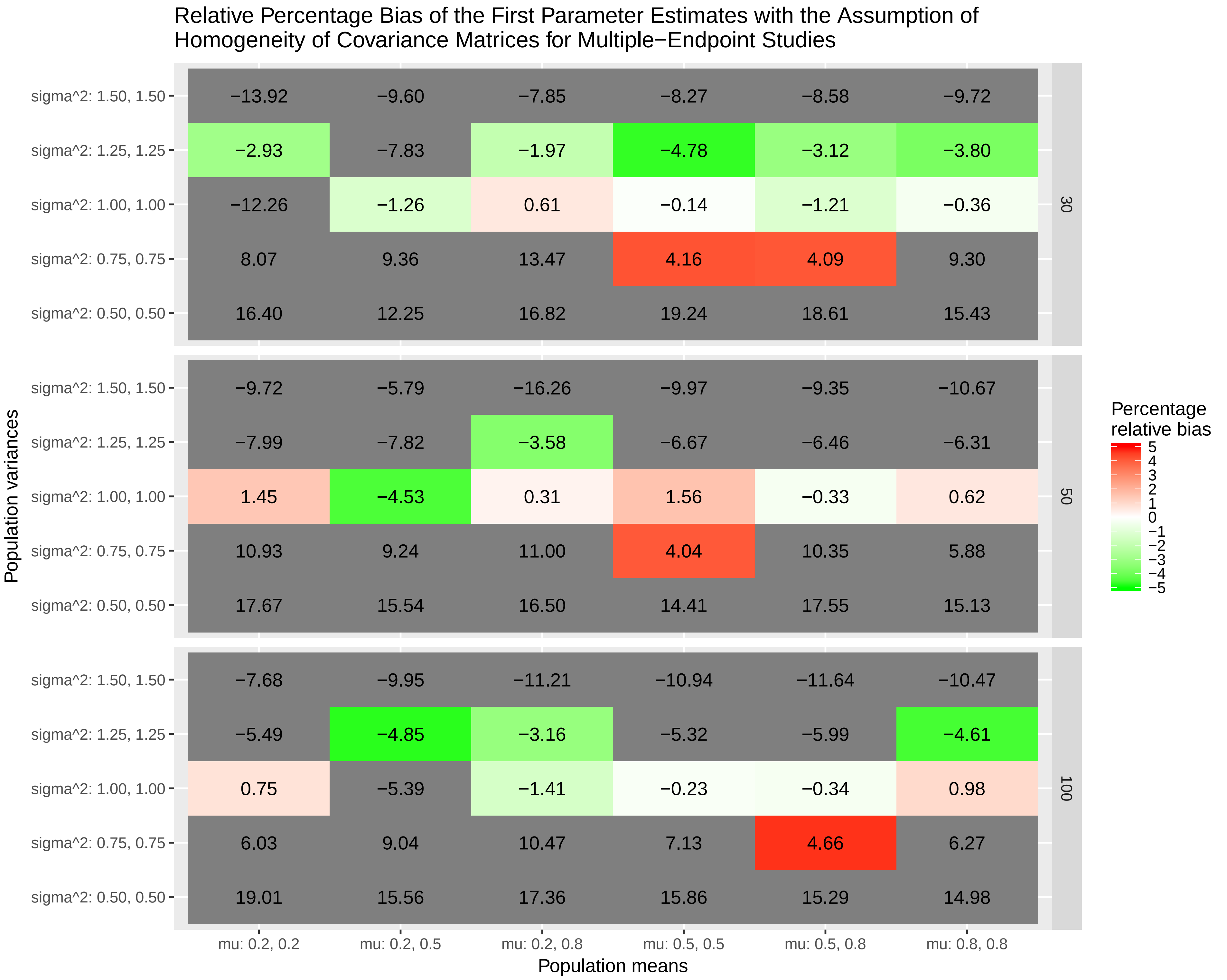
Relative Percentage Bias of the First Parameter Estimates with the Assumption of Homogeneity of Covariance Matrices for Multiple−Endpoint Studies
Population variances
Population means
Percentage relative bias
sigma^2: 1.50, 1.50
sigma^2: 1.25, 1.25
sigma^2: 1.00, 1.00
sigma^2: 0.75, 0.75
sigma^2: 0.50, 0.50
mu: 0.2, 0.2
mu: 0.2, 0.5
mu: 0.2, 0.8
mu: 0.5, 0.5
mu: 0.5, 0.8
mu: 0.8, 0.8
30
50
100
−13.92 −9.60 −7.85 −8.27 −8.58 −9.72
−2.93 −7.83 −1.97 −4.78 −3.12 −3.80
−12.26 −1.26 0.61 −0.14 −1.21 −0.36
8.07 9.36 13.47 4.16 4.09 9.30
16.40 12.25 16.82 19.24 18.61 15.43
−9.72 −5.79 −16.26 −9.97 −9.35 −10.67
−7.99 −7.82 −3.58 −6.67 −6.46 −6.31
1.45 −4.53 0.31 1.56 −0.33 0.62
10.93 9.24 11.00 4.04 10.35 5.88
17.67 15.54 16.50 14.41 17.55 15.13
−7.68 −9.95 −11.21 −10.94 −11.64 −10.47
−5.49 −4.85 −3.16 −5.32 −5.99 −4.61
0.75 −5.39 −1.41 −0.23 −0.34 0.98
6.03 9.04 10.47 7.13 4.66 6.27
19.01 15.56 17.36 15.86 15.29 14.98

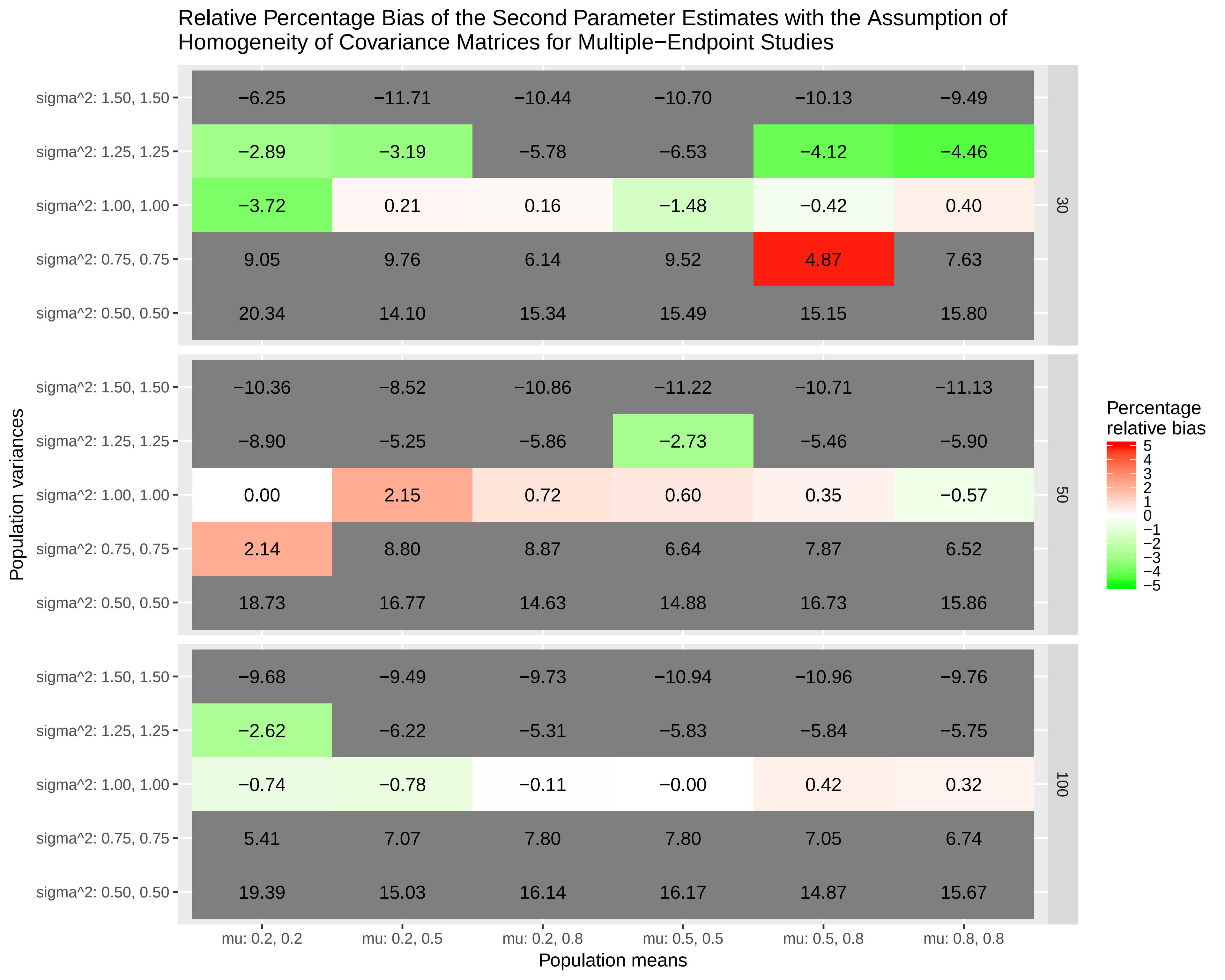
Relative Percentage Bias of the Second Parameter Estimates with the Assumption of Homogeneity of Covariance Matrices for Multiple−Endpoint Studies
Population variances
Population means
sigma^2: 1.50, 1.50
sigma^2: 1.25, 1.25
sigma^2: 1.00, 1.00
sigma^2: 0.75, 0.75
sigma^2: 0.50, 0.50
mu: 0.2, 0.2
mu: 0.2, 0.5
mu: 0.2, 0.8
mu: 0.5, 0.5
mu: 0.5, 0.8
mu: 0.8, 0.8
30
50
100
Percentage relative bias
−6.25 −11.71 −10.44 −10.70 −10.13 −9.49
−2.89 −3.19 −5.78 −6.53 −4.12 −4.46
−3.72 0.21 0.16 −1.48 −0.42 0.40
9.05 9.76 6.14 9.52 4.87 7.63
20.34 14.10 15.34 15.49 15.15 15.80
−10.36 −8.52 −10.86 −11.22 −10.71 −11.13
−8.90 −5.25 −5.86 −2.73 −5.46 −5.90
0.00 2.15 0.72 0.60 0.35 −0.57
2.14 8.80 8.87 6.64 7.87 6.52
18.73 16.77 14.63 14.88 16.73 15.86
−9.68 −9.49 −9.73 −10.94 −10.96 −9.76
−2.62 −6.22 −5.31 −5.83 −5.84 −5.75
−0.74 −0.78 −0.11 −0.00 0.42 0.32
5.41 7.07 7.80 7.80 7.05 6.74
19.39 15.03 16.14 16.17 14.87 15.67

Relative Percentage Bias of the First Parameter Estimates without the Assumption of Homogeneity of Covariance Matrices for Multiple−Endpoint Studies

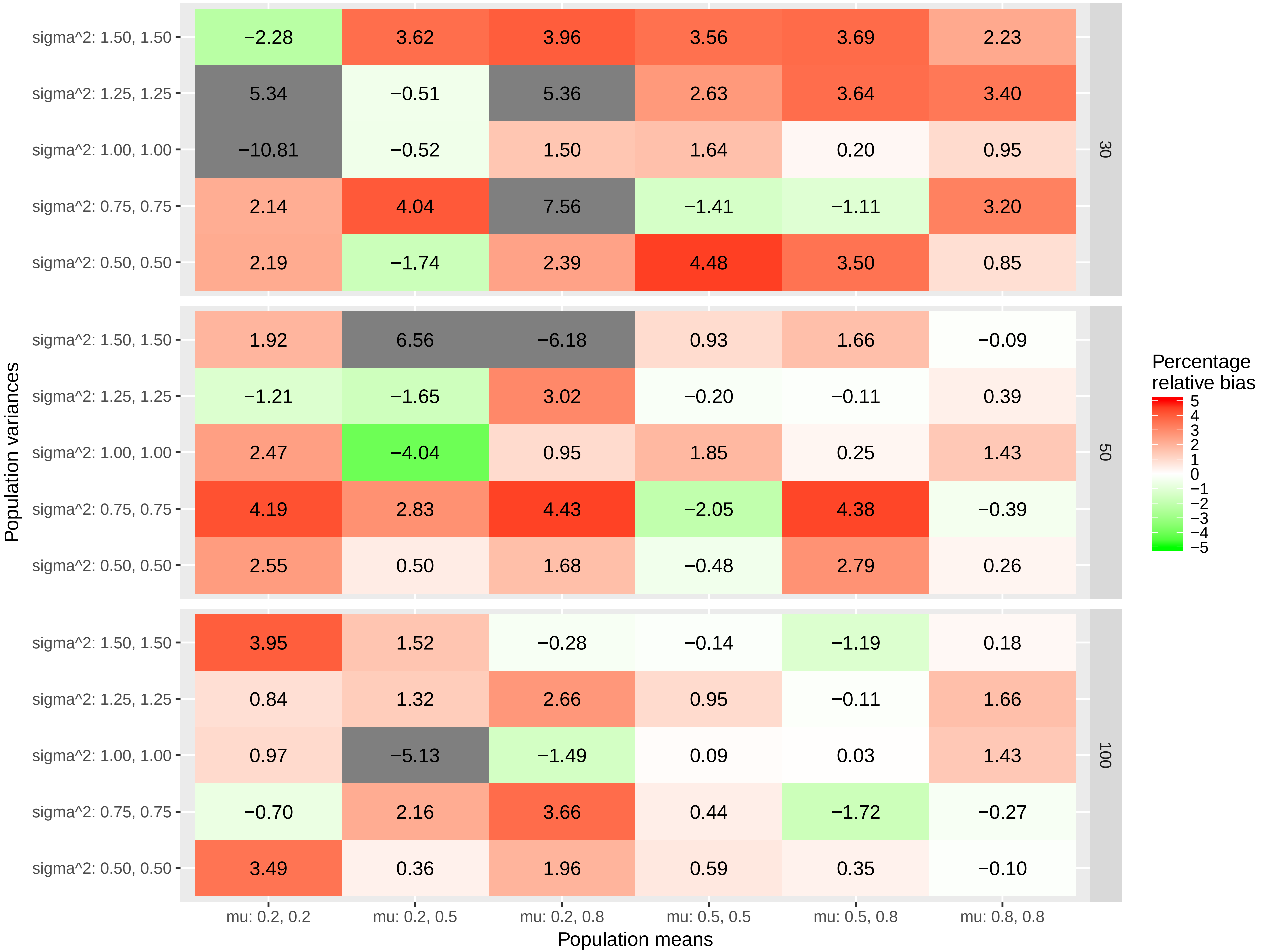

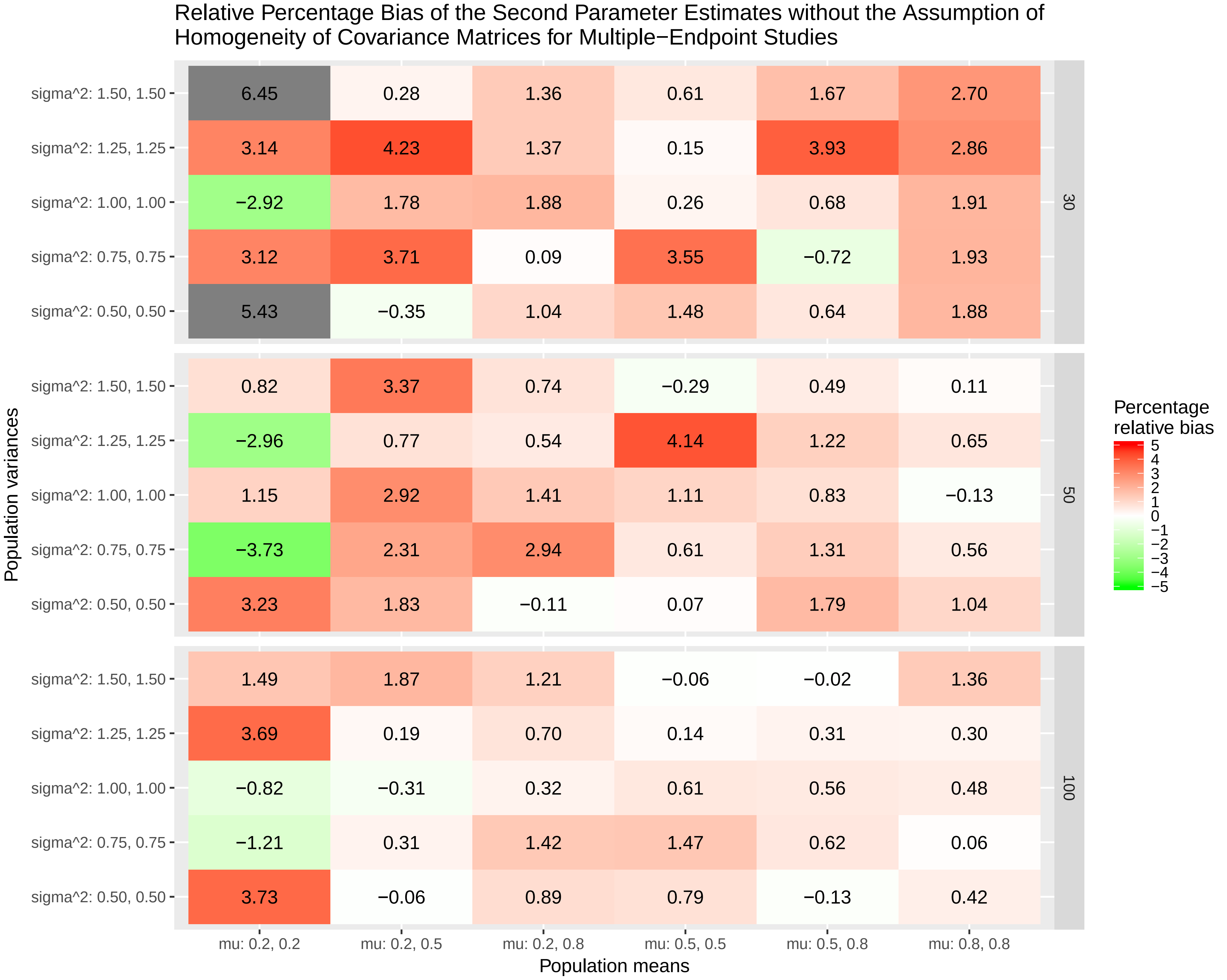
Relative Percentage Bias of the Second Parameter Estimates without the Assumption of Homogeneity of Covariance Matrices for Multiple−Endpoint Studies
Population variances
Population means
mu: 0.2, 0.2
mu: 0.2, 0.5
mu: 0.2, 0.8
mu: 0.5, 0.5
mu: 0.5, 0.8
mu: 0.8, 0.8
sigma^2: 1.50, 1.50
sigma^2: 1.25, 1.25
sigma^2: 1.00, 1.00
sigma^2: 0.75, 0.75
sigma^2: 0.50, 0.50
30
6.45 0.28 1.36 0.61 1.67 2.70
3.14 4.23 1.37 0.15 3.93 2.86
−2.92 1.78 1.88 0.26 0.68 1.91
3.12 3.71 0.09 3.55 −0.72 1.93
5.43 −0.35 1.04 1.48 0.64 1.88
50
0.82 3.37 0.74 −0.29 0.49 0.11
−2.96 0.77 0.54 4.14 1.22 0.65
1.15 2.92 1.41 1.11 0.83 −0.13
−3.73 2.31 2.94 0.61 1.31 0.56
3.23 1.83 −0.11 0.07 1.79 1.04
100
1.49 1.87 1.21 −0.06 −0.02 1.36
3.69 0.19 0.70 0.14 0.31 0.30
−0.82 −0.31 0.32 0.61 0.56 0.48
−1.21 0.31 1.42 1.47 0.62 0.06
3.73 −0.06 0.89 0.79 −0.13 0.42
Percentage relative bias
5
4
3
2
1
0
−1
−2
−3
−4
−5

Relative Percentage Bias of the First Estimated Standard Errors
with the Assumption of Homogeneity of Covariance Matrices for Multiple−Endpoint Studies

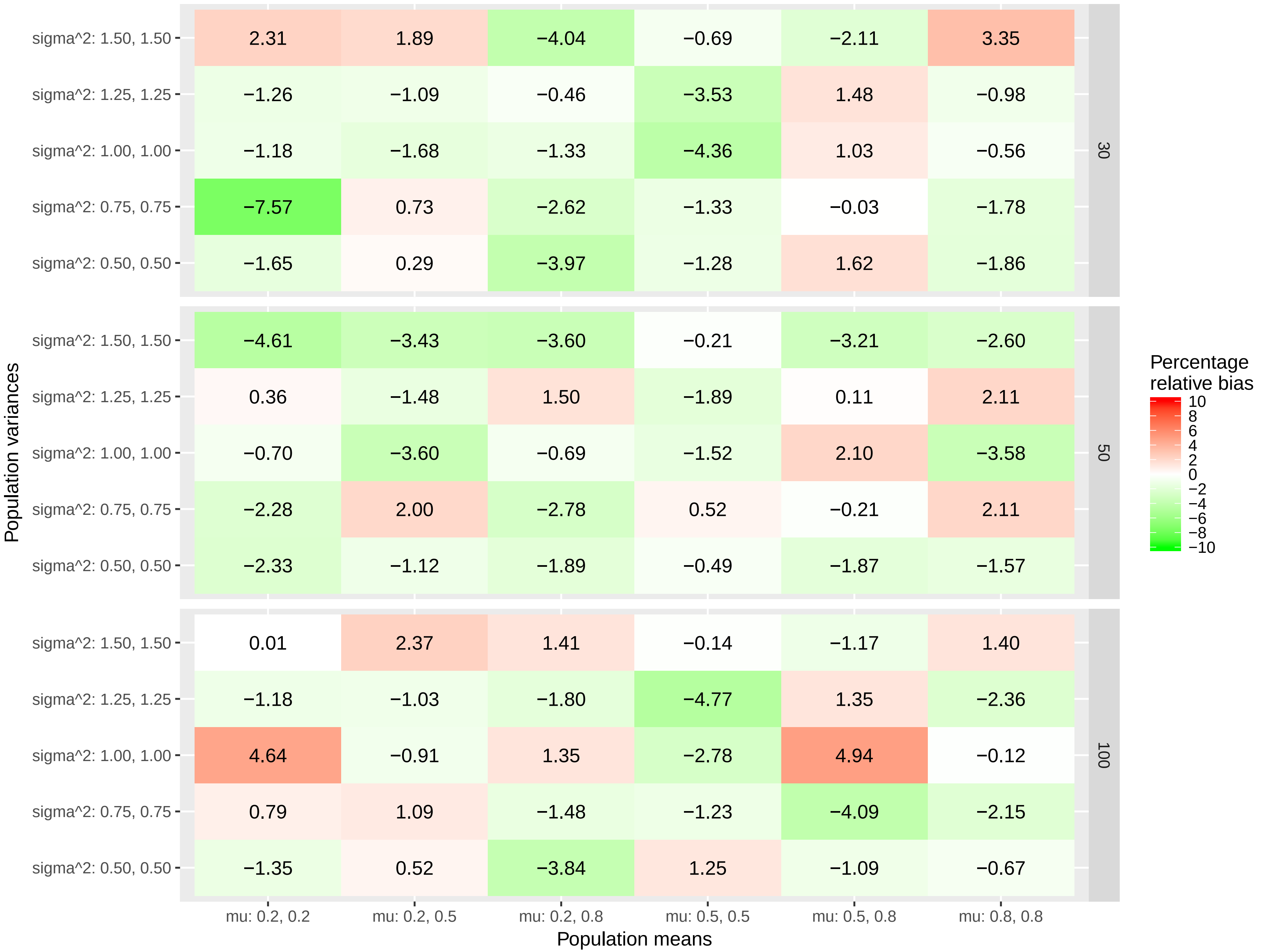

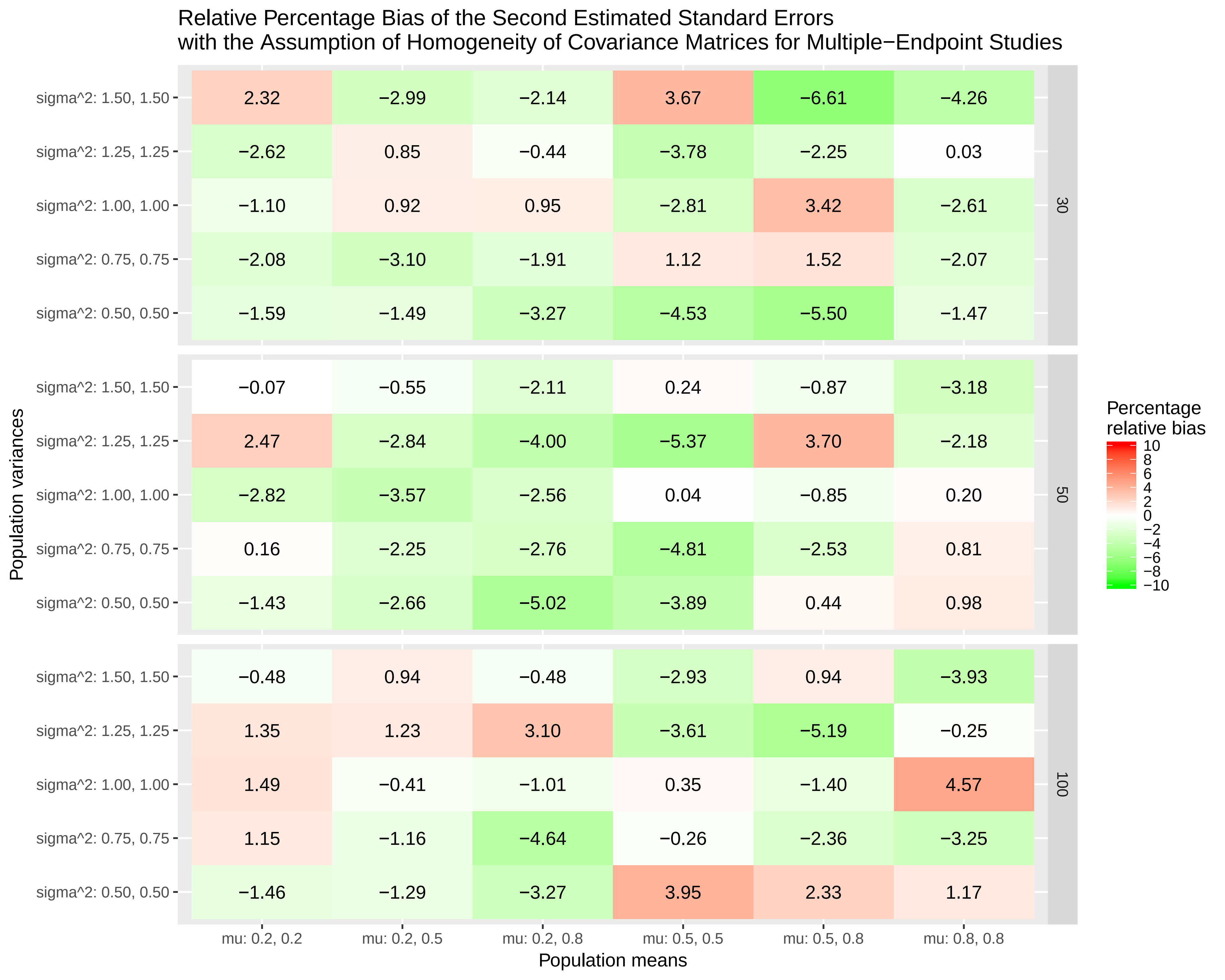
Relative Percentage Bias of the Second Estimated Standard Errors
with the Assumption of Homogeneity of Covariance Matrices for Multiple−Endpoint Studies
30
sigma^2: 1.50, 1.50
2.32 −2.99 −2.14 3.67 −6.61 −4.26
sigma^2: 1.25, 1.25
−2.62 0.85 −0.44 −3.78 −2.25 0.03
sigma^2: 1.00, 1.00
−1.10 0.92 0.95 −2.81 3.42 −2.61
sigma^2: 0.75, 0.75
−2.08 −3.10 −1.91 1.12 1.52 −2.07
sigma^2: 0.50, 0.50
−1.59 −1.49 −3.27 −4.53 −5.50 −1.47
50
sigma^2: 1.50, 1.50
−0.07 −0.55 −2.11 0.24 −0.87 −3.18
sigma^2: 1.25, 1.25
2.47 −2.84 −4.00 −5.37 3.70 −2.18
sigma^2: 1.00, 1.00
−2.82 −3.57 −2.56 0.04 −0.85 0.20
sigma^2: 0.75, 0.75
0.16 −2.25 −2.76 −4.81 −2.53 0.81
sigma^2: 0.50, 0.50
−1.43 −2.66 −5.02 −3.89 0.44 0.98
100
sigma^2: 1.50, 1.50
−0.48 0.94 −0.48 −2.93 0.94 −3.93
sigma^2: 1.25, 1.25
1.35 1.23 3.10 −3.61 −5.19 −0.25
sigma^2: 1.00, 1.00
1.49 −0.41 −1.01 0.35 −1.40 4.57
sigma^2: 0.75, 0.75
1.15 −1.16 −4.64 −0.26 −2.36 −3.25
sigma^2: 0.50, 0.50
−1.46 −1.29 −3.27 3.95 2.33 1.17
Population variances
mu: 0.2, 0.2
mu: 0.2, 0.5
mu: 0.2, 0.8
mu: 0.5, 0.5
mu: 0.5, 0.8
mu: 0.8, 0.8
Population means
Percentage
relative bias
10
8
6
4
2
0
−2
−4
−6
−8
−10

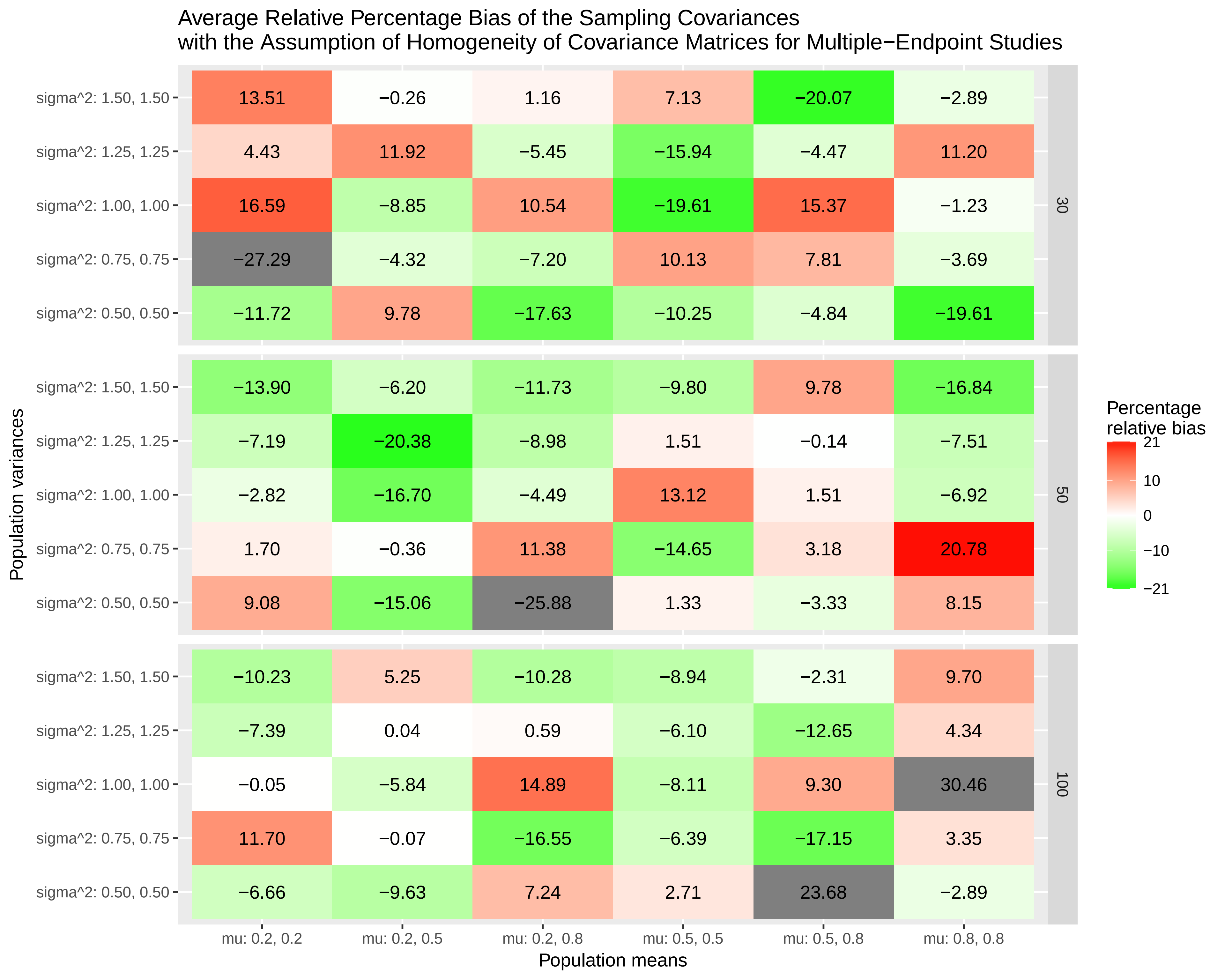

Average Relative Percentage Bias of the Sampling Covariances
with the Assumption of Homogeneity of Covariance Matrices for Multiple−Endpoint Studies
30
sigma^2: 1.50, 1.50
13.51 −0.26 1.16 7.13 −20.07 −2.89
sigma^2: 1.25, 1.25
4.43 11.92 −5.45 −15.94 −4.47 11.20
sigma^2: 1.00, 1.00
16.59 −8.85 10.54 −19.61 15.37 −1.23
sigma^2: 0.75, 0.75
−27.29 −4.32 −7.20 10.13 7.81 −3.69
sigma^2: 0.50, 0.50
−11.72 9.78 −17.63 −10.25 −4.84 −19.61
50
sigma^2: 1.50, 1.50
−13.90 −6.20 −11.73 −9.80 9.78 −16.84
sigma^2: 1.25, 1.25
−7.19 −20.38 −8.98 1.51 −0.14 −7.51
sigma^2: 1.00, 1.00
−2.82 −16.70 −4.49 13.12 1.51 −6.92
sigma^2: 0.75, 0.75
1.70 −0.36 11.38 −14.65 3.18 20.78
sigma^2: 0.50, 0.50
9.08 −15.06 −25.88 1.33 −3.33 8.15
100
sigma^2: 1.50, 1.50
−10.23 5.25 −10.28 −8.94 −2.31 9.70
sigma^2: 1.25, 1.25
−7.39 0.04 0.59 −6.10 −12.65 4.34
sigma^2: 1.00, 1.00
−0.05 −5.84 14.89 −8.11 9.30 30.46
sigma^2: 0.75, 0.75
11.70 −0.07 −16.55 −6.39 −17.15 3.35
sigma^2: 0.50, 0.50
−6.66 −9.63 7.24 2.71 23.68 −2.89
Population variances
mu: 0.2, 0.2
mu: 0.2, 0.5
mu: 0.2, 0.8
mu: 0.5, 0.5
mu: 0.5, 0.8
mu: 0.8, 0.8
Population means
Percentage
relative bias
21
10
0
−10
−21

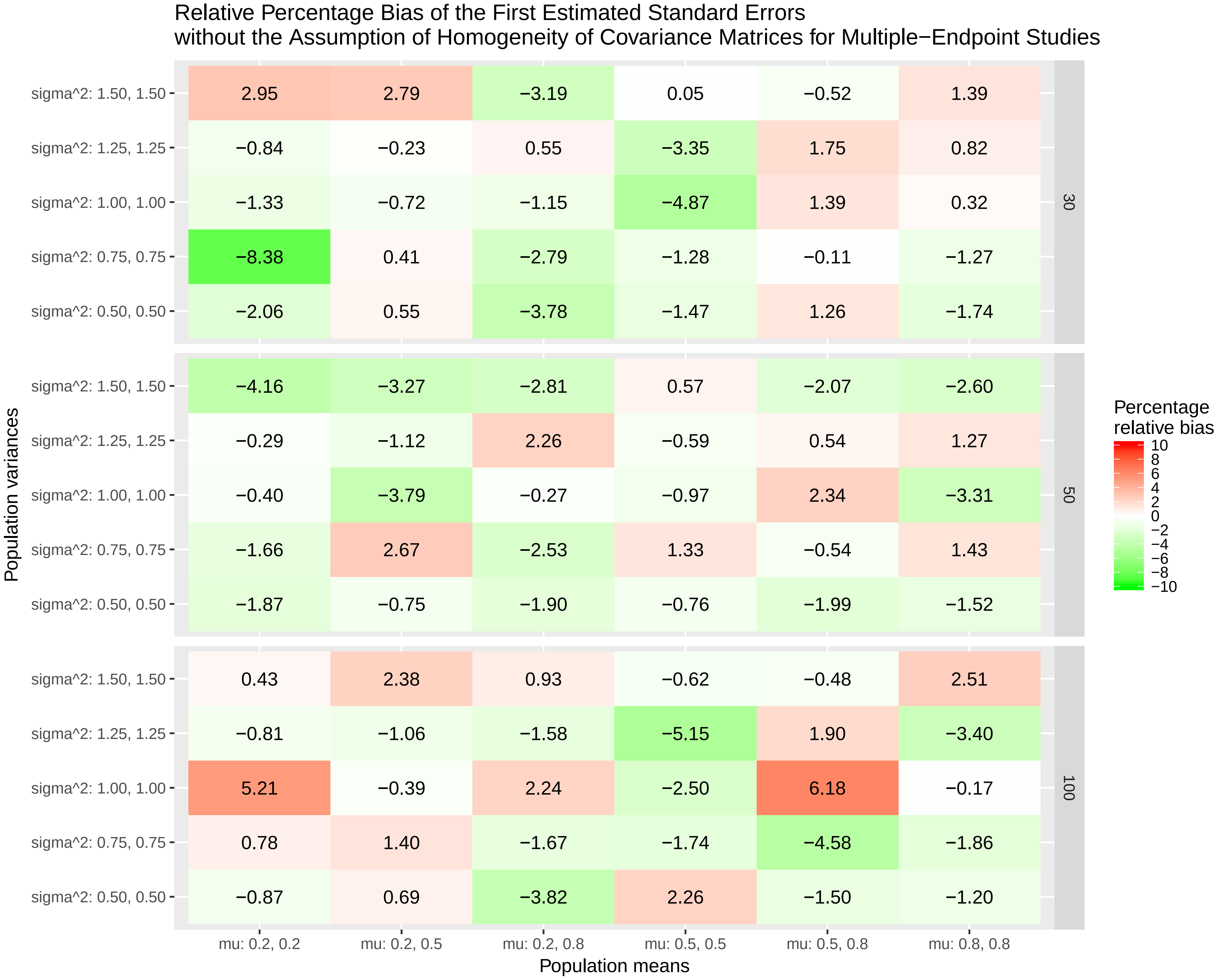
Relative Percentage Bias of the First Estimated Standard Errors
without the Assumption of Homogeneity of Covariance Matrices for Multiple−Endpoint Studies
Population variances
Population means
mu: 0.2, 0.2
mu: 0.2, 0.5
mu: 0.2, 0.8
mu: 0.5, 0.5
mu: 0.5, 0.8
mu: 0.8, 0.8
30
sigma^2: 1.50, 1.50 2.95 2.79 −3.19 0.05 −0.52 1.39
sigma^2: 1.25, 1.25 −0.84 −0.23 0.55 −3.35 1.75 0.82
sigma^2: 1.00, 1.00 −1.33 −0.72 −1.15 −4.87 1.39 0.32
sigma^2: 0.75, 0.75 −8.38 0.41 −2.79 −1.28 −0.11 −1.27
sigma^2: 0.50, 0.50 −2.06 0.55 −3.78 −1.47 1.26 −1.74
50
sigma^2: 1.50, 1.50 −4.16 −3.27 −2.81 0.57 −2.07 −2.60
sigma^2: 1.25, 1.25 −0.29 −1.12 2.26 −0.59 0.54 1.27
sigma^2: 1.00, 1.00 −0.40 −3.79 −0.27 −0.97 2.34 −3.31
sigma^2: 0.75, 0.75 −1.66 2.67 −2.53 1.33 −0.54 1.43
sigma^2: 0.50, 0.50 −1.87 −0.75 −1.90 −0.76 −1.99 −1.52
100
sigma^2: 1.50, 1.50 0.43 2.38 0.93 −0.62 −0.48 2.51
sigma^2: 1.25, 1.25 −0.81 −1.06 −1.58 −5.15 1.90 −3.40
sigma^2: 1.00, 1.00 5.21 −0.39 2.24 −2.50 6.18 −0.17
sigma^2: 0.75, 0.75 0.78 1.40 −1.67 −1.74 −4.58 −1.86
sigma^2: 0.50, 0.50 −0.87 0.69 −3.82 2.26 −1.50 −1.20
Percentage
relative bias
10
8
6
4
2
0
−2
−4
−6
−8
−10

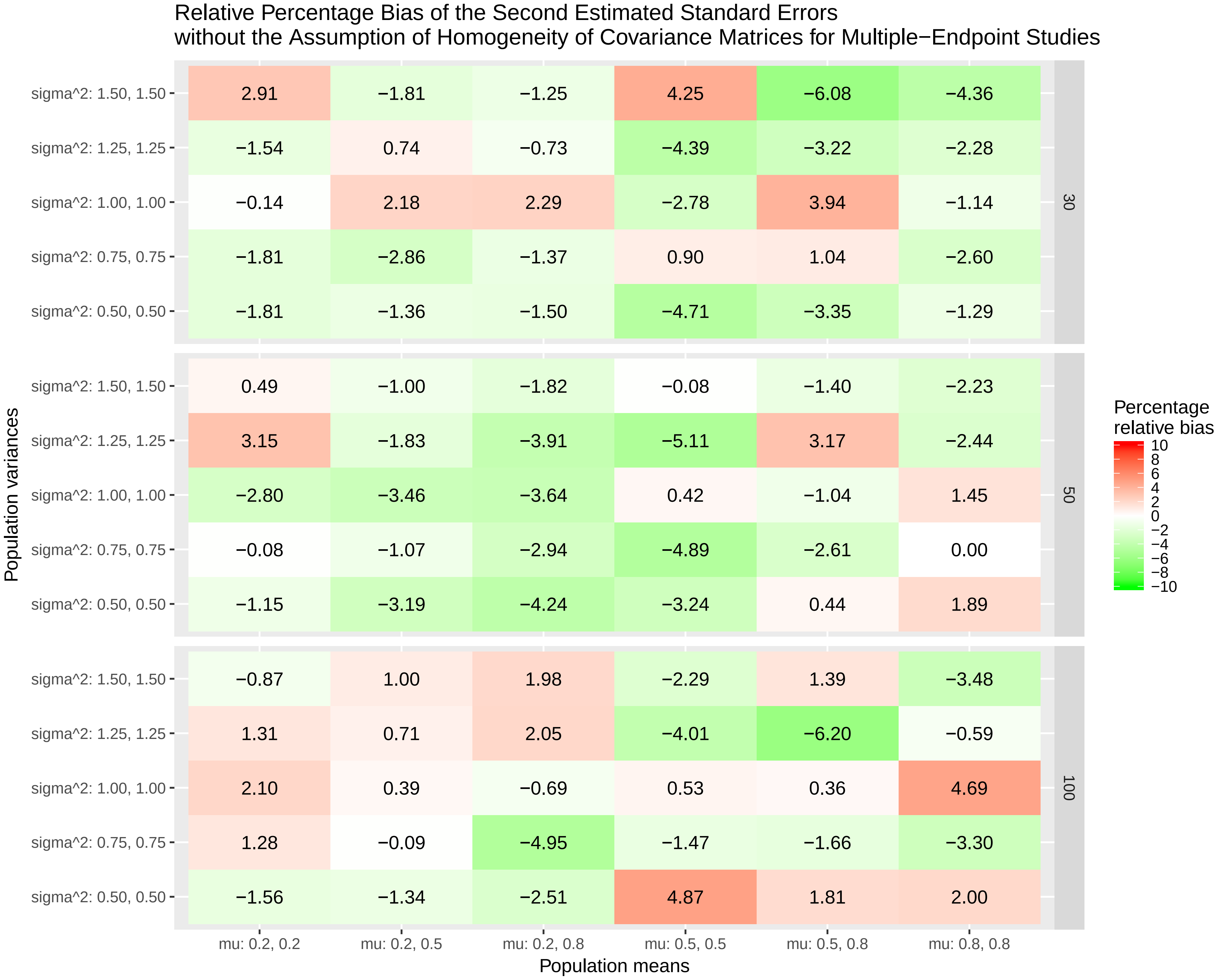
Relative Percentage Bias of the Second Estimated Standard Errors
without the Assumption of Homogeneity of Covariance Matrices for Multiple−Endpoint Studies
Population variances
Population means
mu: 0.2, 0.2
mu: 0.2, 0.5
mu: 0.2, 0.8
mu: 0.5, 0.5
mu: 0.5, 0.8
mu: 0.8, 0.8
sigma^2: 1.50, 1.50
sigma^2: 1.25, 1.25
sigma^2: 1.00, 1.00
sigma^2: 0.75, 0.75
sigma^2: 0.50, 0.50
30
50
100
Percentage
relative bias
10
8
6
4
2
0
−2
−4
−6
−8
−10
2.91 −1.81 −1.25 4.25 −6.08 −4.36
−1.54 0.74 −0.73 −4.39 −3.22 −2.28
−0.14 2.18 2.29 −2.78 3.94 −1.14
−1.81 −2.86 −1.37 0.90 1.04 −2.60
−1.81 −1.36 −1.50 −4.71 −3.35 −1.29
0.49 −1.00 −1.82 −0.08 −1.40 −2.23
3.15 −1.83 −3.91 −5.11 3.17 −2.44
−2.80 −3.46 −3.64 0.42 −1.04 1.45
−0.08 −1.07 −2.94 −4.89 −2.61 0.00
−1.15 −3.19 −4.24 −3.24 0.44 1.89
−0.87 1.00 1.98 −2.29 1.39 −3.48
1.31 0.71 2.05 −4.01 −6.20 −0.59
2.10 0.39 −0.69 0.53 0.36 4.69
1.28 −0.09 −4.95 −1.47 −1.66 −3.30
−1.56 −1.34 −2.51 4.87 1.81 2.00

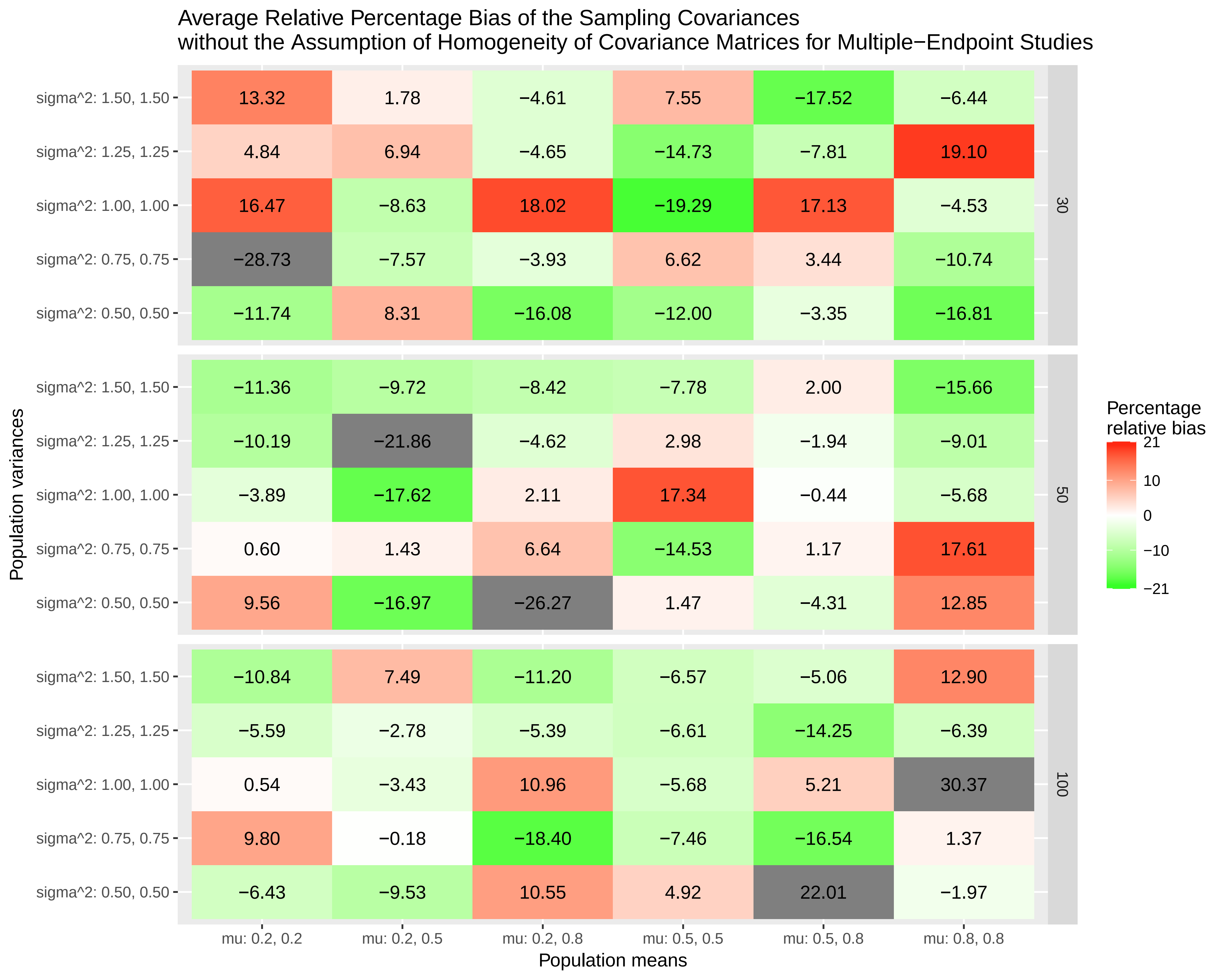
Average Relative Percentage Bias of the Sampling Covariances
without the Assumption of Homogeneity of Covariance Matrices for Multiple−Endpoint Studies
sigma^2: 1.50, 1.50
sigma^2: 1.25, 1.25
sigma^2: 1.00, 1.00
sigma^2: 0.75, 0.75
sigma^2: 0.50, 0.50
13.32
1.78
−4.61
7.55
−17.52
−6.44
4.84
6.94
−4.65
−14.73
−7.81
19.10
16.47
−8.63
18.02
−19.29
17.13
−4.53
−28.73
−7.57
−3.93
6.62
3.44
−10.74
−11.74
8.31
−16.08
−12.00
−3.35
−16.81
30
−11.36
−9.72
−8.42
−7.78
2.00
−15.66
−10.19
−21.86
−4.62
2.98
−1.94
−9.01
−3.89
−17.62
2.11
17.34
−0.44
−5.68
0.60
1.43
6.64
−14.53
1.17
17.61
9.56
−16.97
−26.27
1.47
−4.31
12.85
50
−10.84
7.49
−11.20
−6.57
−5.06
12.90
−5.59
−2.78
−5.39
−6.61
−14.25
−6.39
0.54
−3.43
10.96
−5.68
5.21
30.37
9.80
−0.18
−18.40
−7.46
−16.54
1.37
−6.43
−9.53
10.55
4.92
22.01
−1.97
100
Population variances
mu: 0.2, 0.2
mu: 0.2, 0.5
mu: 0.2, 0.8
mu: 0.5, 0.5
mu: 0.5, 0.8
mu: 0.8, 0.8
Population means
Percentage
relative bias
21
10
0
−10
−21
