# Supplementary material for: Computing Multivariate Effect Sizes and Their Sampling Covariance Matrices With Structural Equation Modeling: Theory, Examples, and Computer Simulations
Source: Front Psychol. 2018 Aug 17;9:1387. doi: 10.3389/fpsyg.2018.01387 (PMC6107852; doi:10.3389/fpsyg.2018.01387)
Supplement: Supplementary file 4 [file Data_Sheet_4.PDF]

Average Relative Percentage Bias of the Parameter Estimates with the Assumption of Homogeneity of Covariance Matrices for Multiple−Endpoint Studies

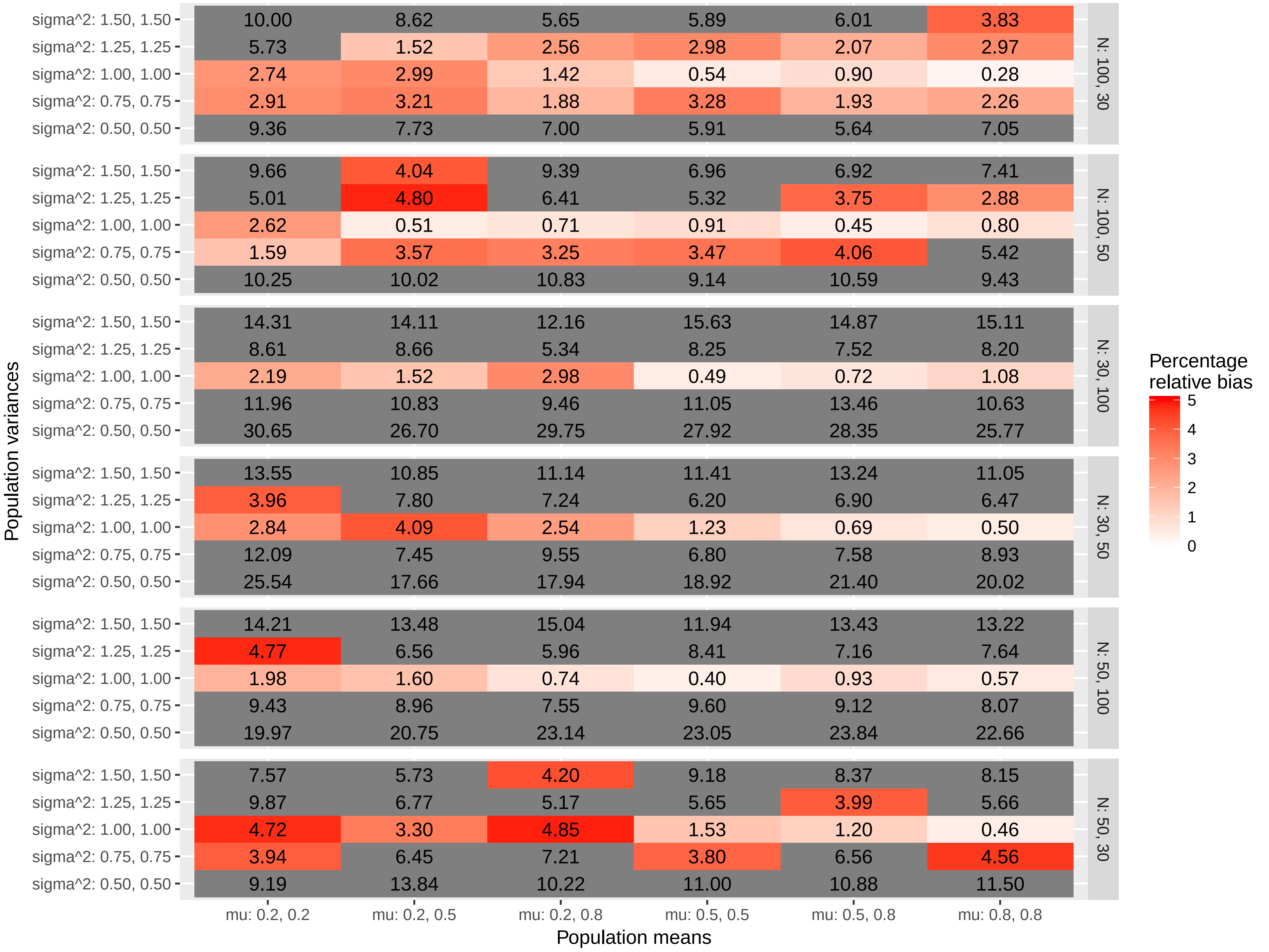

Average Relative Percentage Bias of the Parameter Estimates without the Assumption of Homogeneity of Covariance Matrices for Multiple−Endpoint Studies

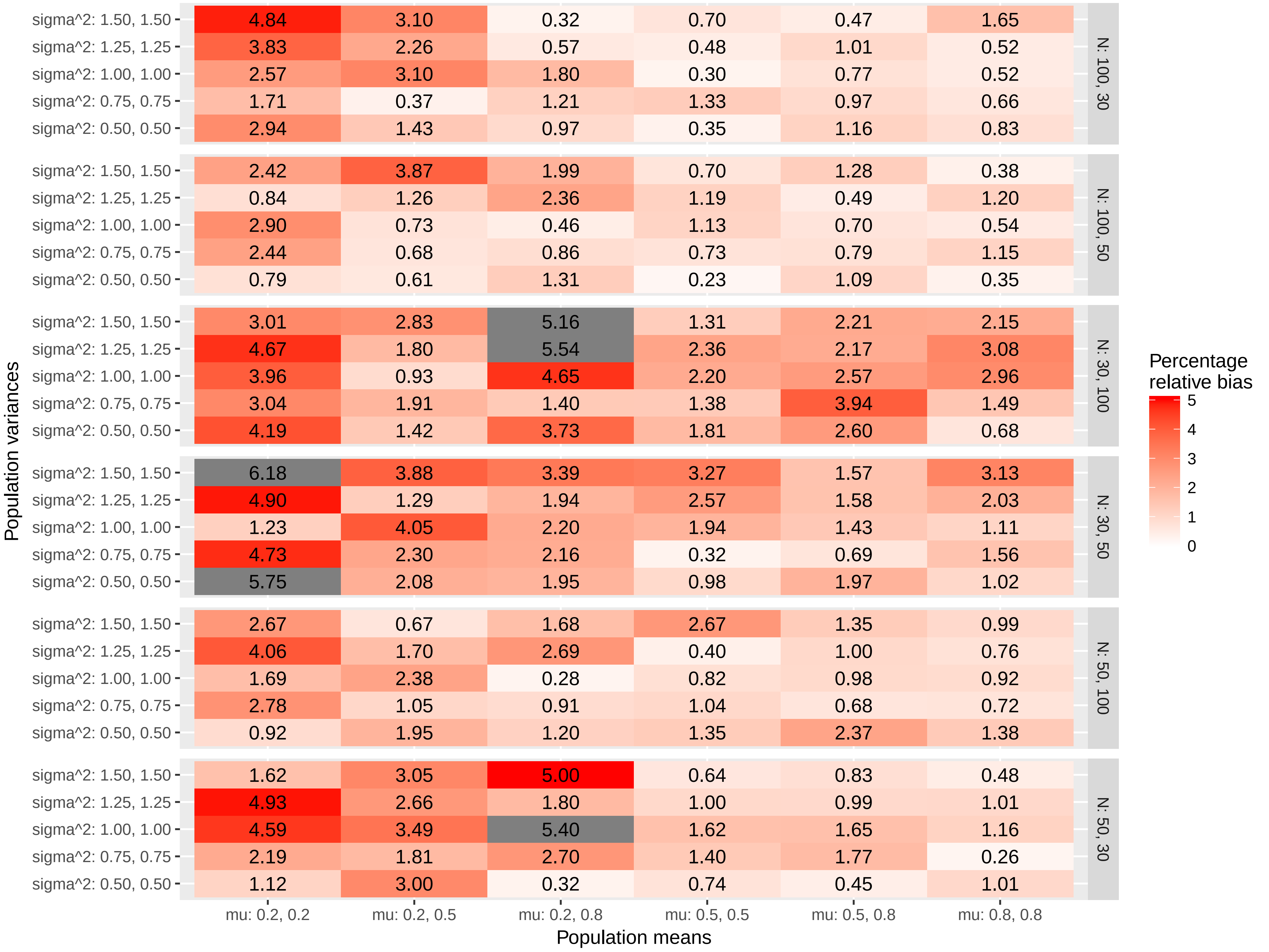

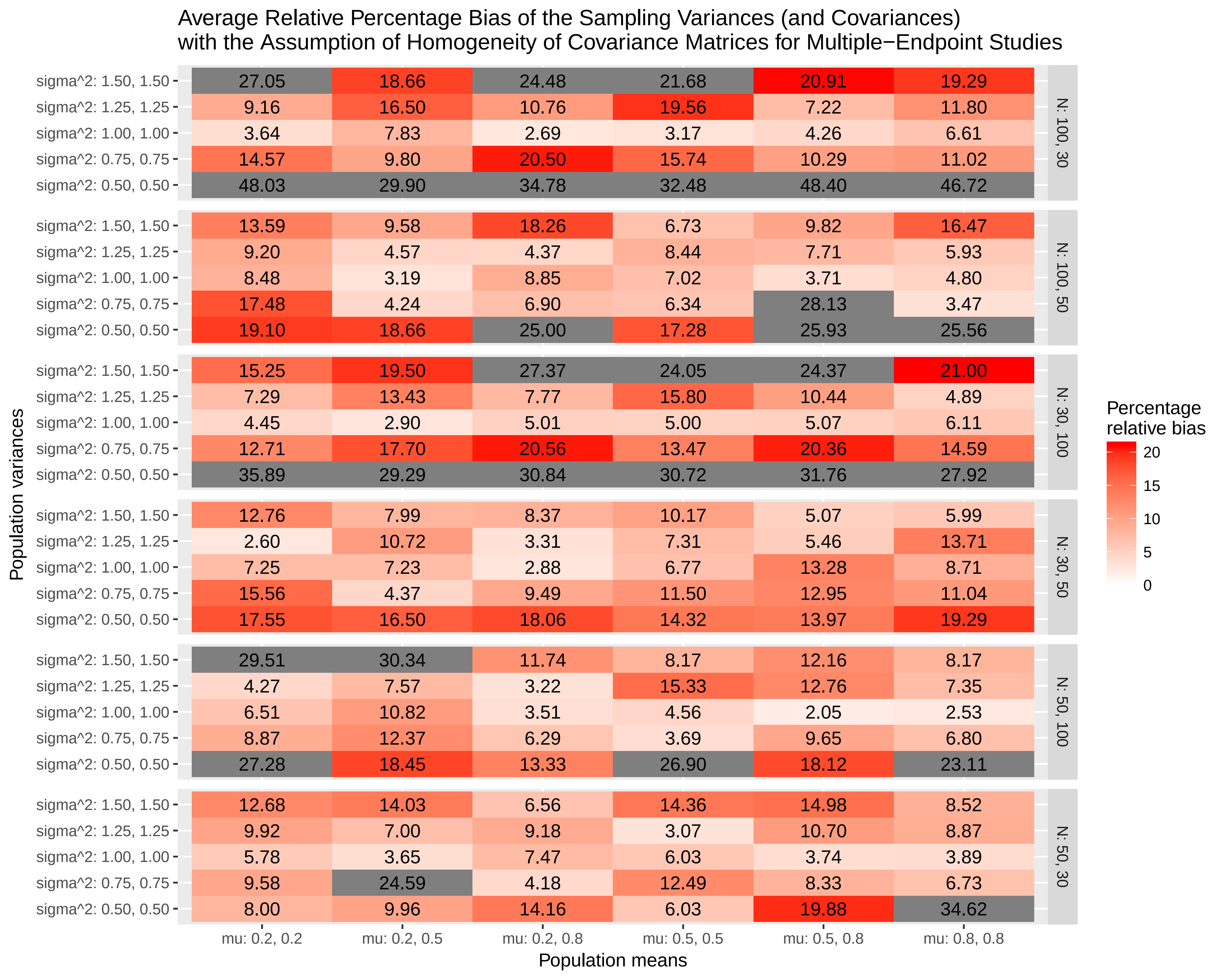

Average Relative Percentage Bias of the Sampling Variances (and Covariances)
with the Assumption of Homogeneity of Covariance Matrices for Multiple−Endpoint Studies
Population variances
Population means
mu: 0.2, 0.2
mu: 0.2, 0.5
mu: 0.2, 0.8
mu: 0.5, 0.5
mu: 0.5, 0.8
mu: 0.8, 0.8
Percentage relative bias
0
5
10
15
20
N: 100, 30
sigma^2: 1.50, 1.50 27.05 18.66 24.48 21.68 20.91 19.29
sigma^2: 1.25, 1.25 9.16 16.50 10.76 19.56 7.22 11.80
sigma^2: 1.00, 1.00 3.64 7.83 2.69 3.17 4.26 6.61
sigma^2: 0.75, 0.75 14.57 9.80 20.50 15.74 10.29 11.02
sigma^2: 0.50, 0.50 48.03 29.90 34.78 32.48 48.40 46.72
N: 100, 50
sigma^2: 1.50, 1.50 13.59 9.58 18.26 6.73 9.82 16.47
sigma^2: 1.25, 1.25 9.20 4.57 4.37 8.44 7.71 5.93
sigma^2: 1.00, 1.00 8.48 3.19 8.85 7.02 3.71 4.80
sigma^2: 0.75, 0.75 17.48 4.24 6.90 6.34 28.13 3.47
sigma^2: 0.50, 0.50 19.10 18.66 25.00 17.28 25.93 25.56
N: 30, 100
sigma^2: 1.50, 1.50 15.25 19.50 27.37 24.05 24.37 21.00
sigma^2: 1.25, 1.25 7.29 13.43 7.77 15.80 10.44 4.89
sigma^2: 1.00, 1.00 4.45 2.90 5.01 5.00 5.07 6.11
sigma^2: 0.75, 0.75 12.71 17.70 20.56 13.47 20.36 14.59
sigma^2: 0.50, 0.50 35.89 29.29 30.84 30.72 31.76 27.92
N: 30, 50
sigma^2: 1.50, 1.50 12.76 7.99 8.37 10.17 5.07 5.99
sigma^2: 1.25, 1.25 2.60 10.72 3.31 7.31 5.46 13.71
sigma^2: 1.00, 1.00 7.25 7.23 2.88 6.77 13.28 8.71
sigma^2: 0.75, 0.75 15.56 4.37 9.49 11.50 12.95 11.04
sigma^2: 0.50, 0.50 17.55 16.50 18.06 14.32 13.97 19.29
N: 50, 100
sigma^2: 1.50, 1.50 29.51 30.34 11.74 8.17 12.16 8.17
sigma^2: 1.25, 1.25 4.27 7.57 3.22 15.33 12.76 7.35
sigma^2: 1.00, 1.00 6.51 10.82 3.51 4.56 2.05 2.53
sigma^2: 0.75, 0.75 8.87 12.37 6.29 3.69 9.65 6.80
sigma^2: 0.50, 0.50 27.28 18.45 13.33 26.90 18.12 23.11
N: 50, 30
sigma^2: 1.50, 1.50 12.68 14.03 6.56 14.36 14.98 8.52
sigma^2: 1.25, 1.25 9.92 7.00 9.18 3.07 10.70 8.87
sigma^2: 1.00, 1.00 5.78 3.65 7.47 6.03 3.74 3.89
sigma^2: 0.75, 0.75 9.58 24.59 4.18 12.49 8.33 6.73
sigma^2: 0.50, 0.50 8.00 9.96 14.16 6.03 19.88 34.62

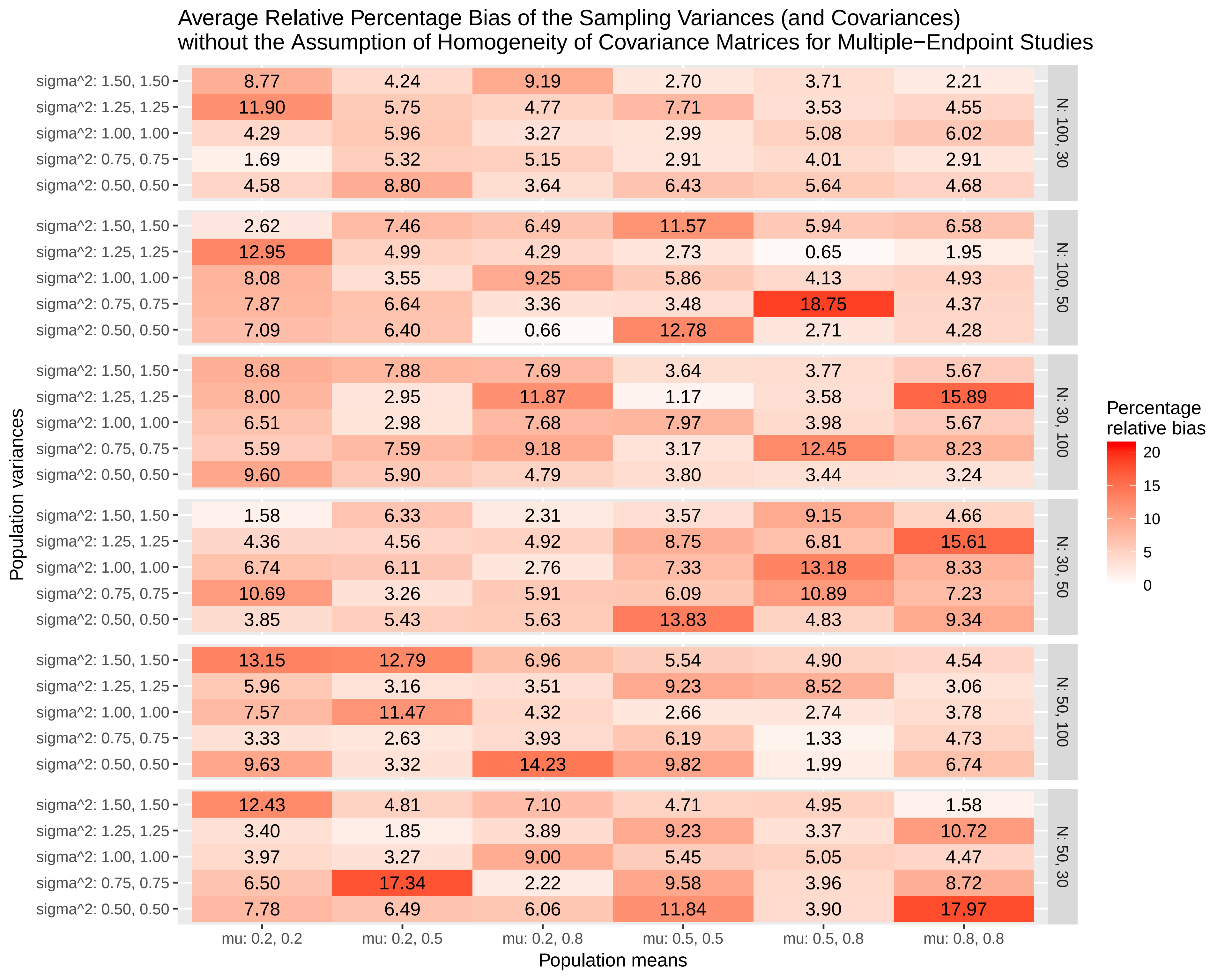
Average Relative Percentage Bias of the Sampling Variances (and Covariances)
without the Assumption of Homogeneity of Covariance Matrices for Multiple−Endpoint Studies
N: 100, 30
sigma^2: 1.50, 1.50 8.77 4.24 9.19 2.70 3.71 2.21
sigma^2: 1.25, 1.25 11.90 5.75 4.77 7.71 3.53 4.55
sigma^2: 1.00, 1.00 4.29 5.96 3.27 2.99 5.08 6.02
sigma^2: 0.75, 0.75 1.69 5.32 5.15 2.91 4.01 2.91
sigma^2: 0.50, 0.50 4.58 8.80 3.64 6.43 5.64 4.68
N: 100, 50
sigma^2: 1.50, 1.50 2.62 7.46 6.49 11.57 5.94 6.58
sigma^2: 1.25, 1.25 12.95 4.99 4.29 2.73 0.65 1.95
sigma^2: 1.00, 1.00 8.08 3.55 9.25 5.86 4.13 4.93
sigma^2: 0.75, 0.75 7.87 6.64 3.36 3.48 18.75 4.37
sigma^2: 0.50, 0.50 7.09 6.40 0.66 12.78 2.71 4.28
N: 30, 100
sigma^2: 1.50, 1.50 8.68 7.88 7.69 3.64 3.77 5.67
sigma^2: 1.25, 1.25 8.00 2.95 11.87 1.17 3.58 15.89
sigma^2: 1.00, 1.00 6.51 2.98 7.68 7.97 3.98 5.67
sigma^2: 0.75, 0.75 5.59 7.59 9.18 3.17 12.45 8.23
sigma^2: 0.50, 0.50 9.60 5.90 4.79 3.80 3.44 3.24
N: 30, 50
sigma^2: 1.50, 1.50 1.58 6.33 2.31 3.57 9.15 4.66
sigma^2: 1.25, 1.25 4.36 4.56 4.92 8.75 6.81 15.61
sigma^2: 1.00, 1.00 6.74 6.11 2.76 7.33 13.18 8.33
sigma^2: 0.75, 0.75 10.69 3.26 5.91 6.09 10.89 7.23
sigma^2: 0.50, 0.50 3.85 5.43 5.63 13.83 4.83 9.34
N: 50, 100
sigma^2: 1.50, 1.50 13.15 12.79 6.96 5.54 4.90 4.54
sigma^2: 1.25, 1.25 5.96 3.16 3.51 9.23 8.52 3.06
sigma^2: 1.00, 1.00 7.57 11.47 4.32 2.66 2.74 3.78
sigma^2: 0.75, 0.75 3.33 2.63 3.93 6.19 1.33 4.73
sigma^2: 0.50, 0.50 9.63 3.32 14.23 9.82 1.99 6.74
N: 50, 30
sigma^2: 1.50, 1.50 12.43 4.81 7.10 4.71 4.95 1.58
sigma^2: 1.25, 1.25 3.40 1.85 3.89 9.23 3.37 10.72
sigma^2: 1.00, 1.00 3.97 3.27 9.00 5.45 5.05 4.47
sigma^2: 0.75, 0.75 6.50 17.34 2.22 9.58 3.96 8.72
sigma^2: 0.50, 0.50 7.78 6.49 6.06 11.84 3.90 17.97
mu: 0.2, 0.2
mu: 0.2, 0.5
mu: 0.2, 0.8
mu: 0.5, 0.5
mu: 0.5, 0.8
mu: 0.8, 0.8
Population means
Population variances
Percentage relative bias
20
15
10
5
0
