# Supplementary material for: Computing Multivariate Effect Sizes and Their Sampling Covariance Matrices With Structural Equation Modeling: Theory, Examples, and Computer Simulations
Source: Front Psychol. 2018 Aug 17;9:1387. doi: 10.3389/fpsyg.2018.01387 (PMC6107852; doi:10.3389/fpsyg.2018.01387)
Supplement: Supplementary file 5 [file Data_Sheet_5.docx]

**Appendix**

**Computing the Effect Sizes and Their Sampling Covariance Matrices with R**

# **Setup**

## You may need to install the required libraries with the following R code.
install.packages(c(“metaSEM”, “lavaan”))

# **Multiple-Treatment Studies**

## Load the libraries for use

library(lavaan)
library(metaSEM)

## First study of the multiple-treatment studies in Gleser and Olkin (1994)

(mts <- Gleser94$MTS[1, ])

## Study N.C N.E1 N.E2 N.E3 N.E4 N.E5 Mean.C Mean.E1 Mean.E2 Mean.E3

## 1 1 25 22 25 23 NA NA 150.96 144.14 139.92 139.32

## Mean.E4 Mean.E5 SD.C SD.E1 SD.E2 SD.E3 SD.E4 SD.E5

## 1 NA NA 8.44 4.25 5.06 3.6 NA NA

## MTS assuming the homogeneity of variances

with(mts, smdMTS(m=c(Mean.C, Mean.E1, Mean.E2, Mean.E3),

v=c(SD.C, SD.E1, SD.E2, SD.E3)^2,

n=c(N.C, N.E1, N.E2, N.E3),

homogeneity="variance"))

## $y

## y2_1 y3_1 y4_1

## -1.170793 -1.897273 -1.998990

##

## $V

## y2_1 y3_1 y4_1

## y2_1 0.08982866 0.05040300 0.05100278

## y3_1 0.05040300 0.09645214 0.05868750

## y4_1 0.05100278 0.05868750 0.10179508

## Testing the assumption of homogeneity of variances

lavaan.1 <- with(mts, smdMTS(m=c(Mean.C, Mean.E1, Mean.E2, Mean.E3),

v=c(SD.C, SD.E1, SD.E2, SD.E3)^2,

n=c(N.C, N.E1, N.E2, N.E3),

homogeneity="variance", lavaan.output = TRUE))

summary(lavaan.1, fit.measures=TRUE)

## lavaan (0.5-23.1097) converged normally after 9 iterations

##

## Number of observations per group

## Group 1 25

## Group 2 22

## Group 3 25

## Group 4 23

##

## Estimator ML

## Minimum Function Test Statistic 21.298

## Degrees of freedom 3

## P-value (Chi-square) 0.000

##

## Chi-square for each group:

##

## Group 1 9.902

## Group 2 3.241

## Group 3 0.708

## Group 4 7.446

##

## Model test baseline model:

##

## Minimum Function Test Statistic 0.000

## Degrees of freedom 0

## P-value NA

##

## User model versus baseline model:

##

## Comparative Fit Index (CFI) 0.000

## Tucker-Lewis Index (TLI) NaN

##

## Loglikelihood and Information Criteria:

##

## Loglikelihood user model (H0) -300.600

## Loglikelihood unrestricted model (H1) -289.952

##

## Number of free parameters 5

## Akaike (AIC) 611.201

## Bayesian (BIC) 623.970

## Sample-size adjusted Bayesian (BIC) 608.184

##

## Root Mean Square Error of Approximation:

##

## RMSEA 0.507

## 90 Percent Confidence Interval 0.318 0.719

## P-value RMSEA <= 0.05 0.000

##

## Standardized Root Mean Square Residual:

##

## SRMR 0.548

##

## Parameter Estimates:

##

## Information Expected

## Standard Errors Standard

##

##

## Group 1 [Group 1]:

##

## Latent Variables:

## Estimate Std.Err z-value P(>|z|)

## lat =~

## x (s1) 5.727 0.416 13.784 0.000

##

## Intercepts:

## Estimate Std.Err z-value P(>|z|)

## .x (m1) 150.960 1.145 131.786 0.000

## lat 0.000

##

## Variances:

## Estimate Std.Err z-value P(>|z|)

## .x 0.000

## lat 1.000

##

##

## Group 2 [Group 2]:

##

## Latent Variables:

## Estimate Std.Err z-value P(>|z|)

## lat =~

## x (s1) 5.727 0.416 13.784 0.000

##

## Intercepts:

## Estimate Std.Err z-value P(>|z|)

## .x (m2) 144.140 1.221 118.041 0.000

## lat 0.000

##

## Variances:

## Estimate Std.Err z-value P(>|z|)

## .x 0.000

## lat 1.000

##

##

## Group 3 [Group 3]:

##

## Latent Variables:

## Estimate Std.Err z-value P(>|z|)

## lat =~

## x (s1) 5.727 0.416 13.784 0.000

##

## Intercepts:

## Estimate Std.Err z-value P(>|z|)

## .x (m3) 139.920 1.145 122.148 0.000

## lat 0.000

##

## Variances:

## Estimate Std.Err z-value P(>|z|)

## .x 0.000

## lat 1.000

##

##

## Group 4 [Group 4]:

##

## Latent Variables:

## Estimate Std.Err z-value P(>|z|)

## lat =~

## x (s1) 5.727 0.416 13.784 0.000

##

## Intercepts:

## Estimate Std.Err z-value P(>|z|)

## .x (m4) 139.320 1.194 116.658 0.000

## lat 0.000

##

## Variances:

## Estimate Std.Err z-value P(>|z|)

## .x 0.000

## lat 1.000

##

## Defined Parameters:

## Estimate Std.Err z-value P(>|z|)

## y2_1 -1.171 0.300 -3.906 0.000

## y3_1 -1.897 0.311 -6.109 0.000

## y4_1 -1.999 0.319 -6.265 0.000

## MTS without the assuming homogeneity of variances

with(mts, smdMTS(m=c(Mean.C, Mean.E1, Mean.E2, Mean.E3),

v=c(SD.C, SD.E1, SD.E2, SD.E3)^2,

n=c(N.C, N.E1, N.E2, N.E3),

homogeneity="none"))

## $y

## y2_1 y3_1 y4_1

## -0.794514 -1.287511 -1.356538

##

## $V

## y2_1 y3_1 y4_1

## y2_1 0.06243818 0.05917078 0.06024063

## y3_1 0.05917078 0.08583620 0.07365745

## y4_1 0.06024063 0.07365745 0.08315624

#

# **Multiple-Endpoint Studies**

## First study of the multiple-endpoint studies in Gleser and Olkin (1994)

(mes <- Gleser94$MES[1, ])

## Study N.Uncoached N.Coached Mean.Uncoached.Math Mean.Uncoached.Verbal

## 1 1 34 21 510 503

## Mean.Coached.Math Mean.Coached.Verbal SD.Uncoached.Math

## 1 620 561 83.6

## SD.Uncoached.Verbal SD.Coached.Math SD.Coached.Verbal Cor.Math.Verbal

## 1 102.4 102.5 78.5 0.66

## Calculate the sampling variances and covariance matrices

Uncoached.V <- with(mes, vec2symMat(c(SD.Uncoached.Math^2,

SD.Uncoached.Math*Cor.Math.Verbal*SD.Uncoached.Verbal,

SD.Uncoached.Verbal^2)))

Uncoached.V

## [,1] [,2]

## [1,] 6988.960 5650.022

## [2,] 5650.022 10485.760

Coached.V <- with(mes, vec2symMat(c(SD.Coached.Math^2,

SD.Coached.Math*Cor.Math.Verbal*SD.Coached.Verbal,

SD.Coached.Verbal^2)))

Coached.V

## [,1] [,2]

## [1,] 10506.250 5310.525

## [2,] 5310.525 6162.250

## MES assuming the homogeneity of covariance matrices

with(mes, smdMES(m1=c(Mean.Uncoached.Math, Mean.Uncoached.Verbal),

m2=c(Mean.Coached.Math, Mean.Coached.Verbal),

V1=Uncoached.V, V2=Coached.V,

n1=N.Uncoached, n2=N.Coached,

homogeneity="covariance", list.output=TRUE))

## $y

## y1 y2

## 1.1879574 0.6082841

##

## $V

## y1 y2

## y1 0.08768541 0.05088347

## y2 0.05088347 0.07821966

## Testing the assumption of homogeneity of variances

lavaan.3 <- with(mes, smdMES(m1=c(Mean.Uncoached.Math,Mean.Uncoached.Verbal),

m2=c(Mean.Coached.Math, Mean.Coached.Verbal),

V1=Uncoached.V,

V2=Coached.V,

n1=N.Uncoached,

n2=N.Coached,

homogeneity="covariance",

lavaan.output=TRUE))

summary(lavaan.3, fit.measures=TRUE)

## lavaan (0.5-23.1097) converged normally after 16 iterations

##

## Number of observations per group

## Group 1 34

## Group 2 21

##

## Estimator ML

## Minimum Function Test Statistic 4.915

## Degrees of freedom 3

## P-value (Chi-square) 0.178

##

## Chi-square for each group:

##

## Group 1 1.790

## Group 2 3.124

##

## Model test baseline model:

##

## Minimum Function Test Statistic 31.460

## Degrees of freedom 2

## P-value 0.000

##

## User model versus baseline model:

##

## Comparative Fit Index (CFI) 0.935

## Tucker-Lewis Index (TLI) 0.957

##

## Loglikelihood and Information Criteria:

##

## Loglikelihood user model (H0) -639.531

## Loglikelihood unrestricted model (H1) -637.073

##

## Number of free parameters 7

## Akaike (AIC) 1293.061

## Bayesian (BIC) 1307.112

## Sample-size adjusted Bayesian (BIC) 1285.116

##

## Root Mean Square Error of Approximation:

##

## RMSEA 0.152

## 90 Percent Confidence Interval 0.000 0.385

## P-value RMSEA <= 0.05 0.204

##

## Standardized Root Mean Square Residual:

##

## SRMR 0.151

##

## Parameter Estimates:

##

## Information Expected

## Standard Errors Standard

##

##

## Group 1 [Group 1]:

##

## Latent Variables:

## Estimate Std.Err z-value P(>|z|)

## lat1 =~

## x1 (s1_1) 91.279 8.703 10.488 0.000

## lat2 =~

## x2 (s2_1) 93.994 8.962 10.488 0.000

##

## Covariances:

## Estimate Std.Err z-value P(>|z|)

## lat1 ~~

## lat2 (.p9.) 0.643 0.079 8.143 0.000

##

## Intercepts:

## Estimate Std.Err z-value P(>|z|)

## .x1 (m1_1) 510.000 15.654 32.579 0.000

## .x2 (m2_1) 503.000 16.120 31.204 0.000

## lat1 0.000

## lat2 0.000

##

## Variances:

## Estimate Std.Err z-value P(>|z|)

## .x1 0.000

## .x2 0.000

## lat1 1.000

## lat2 1.000

##

##

## Group 2 [Group 2]:

##

## Latent Variables:

## Estimate Std.Err z-value P(>|z|)

## lat1 =~

## x1 (s1_1) 91.279 8.703 10.488 0.000

## lat2 =~

## x2 (s2_1) 93.994 8.962 10.488 0.000

##

## Covariances:

## Estimate Std.Err z-value P(>|z|)

## lat1 ~~

## lat2 (.p9.) 0.643 0.079 8.143 0.000

##

## Intercepts:

## Estimate Std.Err z-value P(>|z|)

## .x1 (m1_2) 620.000 19.919 31.126 0.000

## .x2 (m2_2) 561.000 20.511 27.351 0.000

## lat1 0.000

## lat2 0.000

##

## Variances:

## Estimate Std.Err z-value P(>|z|)

## .x1 0.000

## .x2 0.000

## lat1 1.000

## lat2 1.000

##

## Defined Parameters:

## Estimate Std.Err z-value P(>|z|)

## y1 1.188 0.296 4.012 0.000

## y2 0.608 0.280 2.175 0.030

## MES without the assuming homogeneity of variances

with(mes, smdMES(m1=c(Mean.Uncoached.Math, Mean.Uncoached.Verbal),

m2=c(Mean.Coached.Math, Mean.Coached.Verbal),

V1=Uncoached.V, V2=Coached.V,

n1=N.Uncoached, n2=N.Coached,

homogeneity="none", list.output=TRUE))

## $y

## y1 y2

## 1.2970816 0.5583531

##

## $V

## y1 y2

## y1 0.12288573 0.05220909

## y2 0.05220909 0.06036059
